# RNA interference screens discover proteases as synthetic lethal partners of PI3K inhibition in breast cancer cells

**DOI:** 10.7150/thno.68299

**Published:** 2022-05-16

**Authors:** Lena Hölzen, Jan Mitschke, Claudia Schönichen, Maria Elena Hess, Sophia Ehrenfeld, Melanie Boerries, Cornelius Miething, Tilman Brummer, Thomas Reinheckel

**Affiliations:** 1 Institute of Molecular Medicine and Cell Research, Faculty of Medicine, University of Freiburg, 79104 Freiburg, Germany; 2 German Cancer Consortium (DKTK) Partner Site Freiburg, DKFZ, 79104 Freiburg, Germany; 3 German Cancer Research Center (DKFZ), 69120 Heidelberg, Germany; 4 Faculty of Biology, Albert-Ludwigs-University of Freiburg, 79104 Freiburg, Germany; 5 Center for Translational Cell Research, Department of Internal Medicine I, Department of Hematology, Oncology and Stem Cell Transplantation, Faculty of Medicine, University of Freiburg, 79106 Freiburg, Germany; 6 Institute of Medical Bioinformatics and Systems Medicine, Medical Center - University of Freiburg, Faculty of Medicine, University of Freiburg, 79106 Freiburg, Germany; 7 Center for Biological Signaling Studies BIOSS, University of Freiburg, 79104 Freiburg, Germany; 8 Comprehensive Cancer Center Freiburg (CCCF), University Medical Center, University of Freiburg, 79106 Freiburg, Germany

**Keywords:** Proteases, Cancer Degradome, Breast Cancer, RNA Interference, Phosphatidylinositol-3-kinase (PI3K)-Pathway

## Abstract

Rationale: PI3K/mTOR signaling is frequently upregulated in breast cancer making inhibitors of this pathway highly promising anticancer drugs. However, PI3K-inhibitors have a low therapeutic index. Therefore, finding novel combinatory treatment options represents an important step towards clinical implementation of PI3K pathway inhibition in breast cancer therapy. Here, we propose proteases as potential synergistic partners with simultaneous PI3K inhibition in breast cancer cells.

Methods: We performed mRNA expression studies and unbiased functional genetic synthetic lethality screens by a miR-E based knockdown system targeting all genome-encoded proteases, i.e. the degradome of breast cancer cells. Importantly theses RNA interference screens were done in combination with two PI3K pathway inhibitors. Protease hits were validated in human and murine breast cancer cell lines as well as in non-cancerous cells by viability and growth assays.

Results: The degradome-wide genetic screens identified 181 proteases that influenced susceptibility of murine breast cancer cells to low dose PI3K inhibition. Employing independently generated inducible knockdown cell lines we validated 12 protease hits in breast cancer cells. In line with the known tumor promoting function of these proteases we demonstrated Usp7 and Metap2 to be important for murine and human breast cancer cell growth and discovered a role for Metap1 in this context. Most importantly, we demonstrated that Usp7, Metap1 or Metap2 knockdown combined with simultaneous PI3K inhibition resulted in synergistic impairment of murine and human breast cancer cell growth

Conclusion: We successfully established proteases as combinatory targets with PI3K inhibition in human and murine breast cancer cells. Usp7, Metap1 and Metap2 are synthetic lethal partners of simultaneous protease/PI3K inhibition, which may refine future breast cancer therapy.

## Introduction

Breast cancer is a malignant disease that affects one out of eight women during their lifetime (12.9% lifetime risk 1). Due to its cellular and molecular heterogeneity, it is crucial to understand the molecular pathways driving breast cancer in order to improve therapeutic approaches 2-5. The Phosphatidylinositol-3-kinase/mammalian Target of Rapamycin (PI3K/mTOR) signaling pathway regulates essential physiological processes 6-8 and is activated in 70% of breast cancer cases 9-12. Genetic alteration of the *PIK3CA* gene, encoding the catalytic subunit of the PI3Kα isoform, occur in 36% of breast cancer patients 10. Additionally, genetic amplifications of the *PIK3CA* locus and mutations of the tumor suppressor Phosphatase and Tensin homolog (*PTEN*; 4%) drive tumor progression and therapy resistance 5,13,14. Although PI3K-inhibitors are vital anticancer drugs 4,5,7,12,13,15-17, only the PI3Kα isoform-inhibitor Piqray (Alpelisib, Novartis) is FDA-approved for breast cancer so far 18. Multiple inhibitors have been under clinical investigation 5,13,15,16,19 including the reversible pan-class I PI3K-inhibitor NVP-BKM-120 (Buparlisib [further abbreviated as BKM], Novartis 20) and the next-generation selective dual pan-class I PI3K-mTOR-inhibitor NVP‑BEZ‑235 (Dactosilib [further abbreviated as BEZ]; Novartis 21). As the PI3K pathway is also regulating survival and metabolism of healthy cells the therapeutic index of PI3K-inhibitors is quite low and monotherapy is not sufficient to kill breast cancer cells 3,5,12-15. Hence, combinatory therapy is key to improve survival rates, reduce toxic side effects and to fight recurring, therapy resistant breast cancer 17. Combinations of PI3K-inhibitors and standard breast cancer therapy were conducted with some success 16,22-25, but combination with new drug targets leading to synergistic effects might provide alternative therapeutic options.

In the present study, we set out to explore proteases as a fairly neglected class of enzymes in connection to PI3K signaling. Proteases often promote cancer progression and metastasis due to their connection to many signaling pathways 26. In this respect a regulatory crosstalk of proteases and kinases has been postulated 27. However, only few proteases are known to be directly linked to the PI3K/mTOR pathway. For example, the Ubiquitin carboxyl-terminal hydrolase 7 (Usp7) regulates the subcellular localization of PTEN 28 and Usp1 deubiquitinates Akt 29. Proteases might be stronger connected to PI3K signaling than previously anticipated, therefore raising the possibility to engage them as synergistic or synthetic lethal partners of simultaneously applied PI3K-inhibitors in breast cancer therapy. In the concept of synthetic lethality, originally only applied to genetic perturbations, application of one drug or interference with one target has no significant effects on cell viability, while the combination has significant synergistic effects, i.e. the target is synthetically lethal with the compound 30-32. Conceptually, in such screens, cells are treated with a minimal dose of a drug while performing a dropout genetic screen to identify genes whose loss-of-function is lethal only in the presence of the drug. The depletion/drop out of the respective cells from the drug treated population identifies candidate genes as “hits” of the screen.

The goal of the present study was to identify synthetic lethal interactions between proteases and the PI3K/mTOR pathway inhibitors BKM and BEZ in murine breast cancer cells by employing synthetic lethality screens. For our purpose and to allow for an unbiased analysis of the complete set of genome encoded proteases, frequently defined as the degradome 33, a degradome-wide small hairpin RNA (shRNA) library was introduced into a Doxycycline (Dox)-inducible vector system that also expresses constitutive and inducible fluorescent reporter proteins. For the shRNA library we took advantage of a third generation vector‑based RNA interference (RNAi) system, i.e. the enhanced microRNA (miR-E) developed by optimization of the native human miR-30a scaffold 34. The vector optimization introduced mutations in the 5' stem and repositioned the *EcoRI* cleavage site 3´ of the basal stem that restored a conserved ACNNC motive containing element. This resulted in boosted knockdown potency due to increased miR-E processing leading to 10- to 30-fold higher mature shRNA levels. Hence, miR-E constructs are better suited for single-copy integration that is essential for high-throughput pooled RNAi screens 35. By using this miR-E system for our unbiased screening approach, we assessed the effects of protease targeting on the sensitivity of murine breast cancer cells to the two PI3K pathway inhibitors. We further employed inducible protease knockdown breast cancer cell lines to functionally validate screen hits and identified Usp7, Methionine aminopeptidases 1 and 2 (Metap1; Metap2) to be required for the growth of murine and human breast cancer cells and as synthetic lethal partners with PI3K inhibition.

## Results

### Expression of many protease genes is regulated by the PI3K pathway

**Figure 1:**
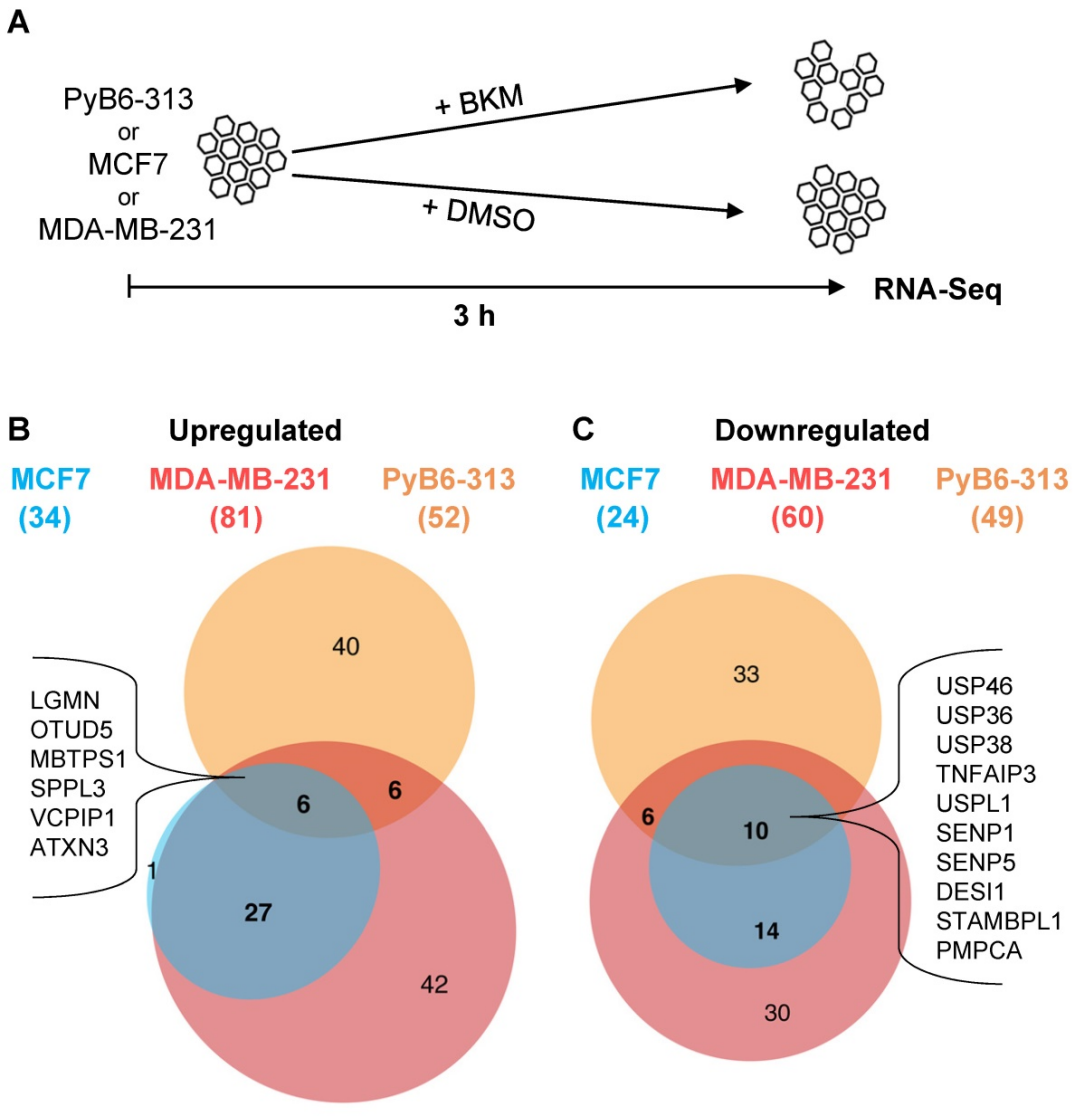
Impact of short-term PI3K inhibition on protease mRNA expression. **A**: Schematic setup of the RNA-Sequencing (RNA-Seq) experiment in human MCF7, MDA-MB-231 and murine PyB6-313 breast cancer cell lines treated for 3 h with BKM EC50 or DMSO. **B**: Upregulation: DMSO- versus BKM treated conditions filtered for adjusted p value ≤ 0.05; expression > 1; LogFC > 0. ( ): Number of significantly upregulated proteases per cell line. Significantly upregulated proteases in all three cell lines: LGMN: Legumain; OTUD5: OTU domain-containing protein 5; MBTPS1: Membrane-bound transcription factor site-1 protease; SPPL3: Signal peptide peptidase-like 3; VCPIP1: Deubiquitinating protein VCPIP1; ATXN3: Ataxin-3. **C**: Downregulation: see B but LogFC < 0. Significantly downregulated proteases in all three cell lines: USP46/36/38: Ubiquitin carboxyl-terminal hydrolase 46/36/38; TNFAIP3: Tumor necrosis factor alpha-induced protein 3; USPL1: SUMO-specific isopeptidase USPL1; SENP1/5: Sentrin-specific protease 1/5; DESI1: Desumoylating isopeptidase 1; STAMBPL1: STAM Binding Protein Like 1; PMPCA: Mitochondrial-processing peptidase subunit alpha. A list of all significantly up- or downregulated proteases can be found in Supplementary Table S1.

Hypothesizing that proteases might be strongly linked to the PI3K pathway, we set out to investigate the connection of proteases and PI3K signaling by mRNA expression analysis. For simplification, the term “protease” used in this publication refers to all degradome-encoded proteins including *bona fide* proteases encoded by a single transcript, protease subunits, and also proteolytically inactive protease-like proteins (pseudoproteases). An RNA-sequencing (RNA-Seq) experiment addressed changes in protease mRNA expression upon short-term PI3K inhibition by the pan-class I PI3K-inhibitor BKM at EC50 in human MCF7 and MDA-MB-231 as well as in murine PyB6-313 breast cancer cells (Figure 1A). The latter were previously generated from transgenic MMTV-PyMT mice in which mammary epithelium directed expression of the polyoma middle T antigen (PyMT) drives breast carcinogenesis by activating receptor tyrosine kinase pathways involved in human breast carcinogenesis, including the PI3K axis 36,37. The mRNA expression of 34 (in MCF7), 81 (in MDA-MB-231), and 52 (in PyB6-313) proteases was significantly upregulated by PI3K inhibition in comparison to DMSO treatment (Figure 1B). Expression of 24 (in MCF7), 60 (in MDA-MB-231), and 49 (in PyB6-313) proteases was significantly downregulated (Figure 1C). Interestingly, the cell lines shared sets of 6 significantly upregulated and 10 downregulated proteases as shown in figure 1B/C. In summary, the data demonstrated a link between PI3K signaling and protease expression, which affects multiple classes and families of proteolytic enzymes.

### Generation of murine breast cancer cells for degradome-focused RNAi experiments

**Figure 2:**
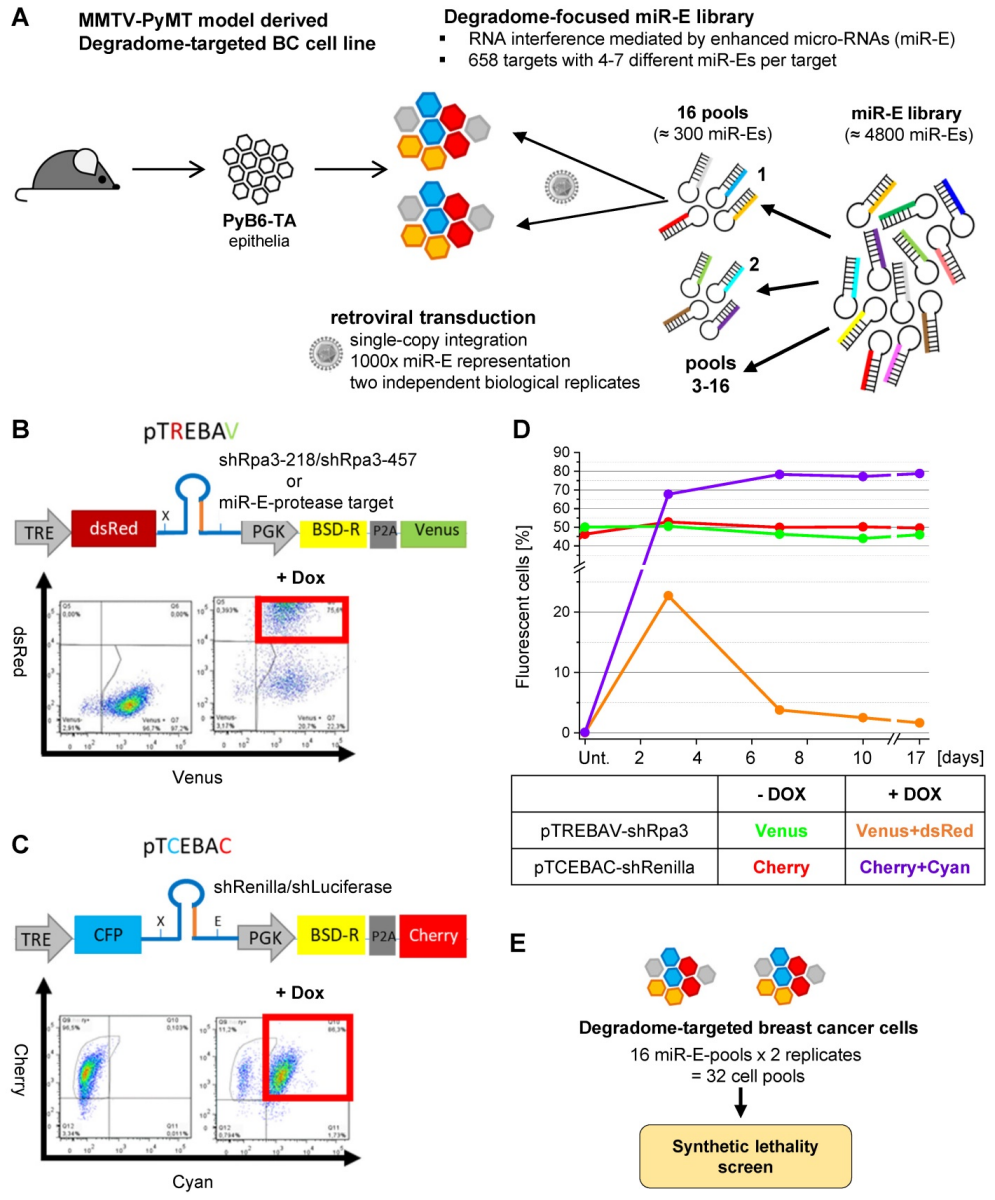
Degradome-targeted murine breast cancer cell pools and controls. **A**: Pipeline for integration of a degradome-focused miR-E-library into MMTV-PyMT mouse model derived breast cancer cells (PyB6-TA). BC: Breast cancer. **B/C**: Schematic representation of retroviral double fluorescent Dox inducible vectors (pTREBAV; pTCEBAC) with exemplary flow cytometry data displaying the percentage of constitutive fluorescent cells (pTREBAV: Venus; pTCEBAC: Cherry) without Dox addition and miR-E-induced fluorescent cells (pTREBAV: dsRed; pTCEBAC: Cyan) after 3 days of Dox treatment (+ Dox). Cells: pTREBAV-shRpa3-457 or pTCEBAC-shLuciferase-transduced PyB6-TA cells. MiR-E backbone in dark blue, guide sequence in orange. TRE: Tetracycline response element; PGK: phosphoglycerate kinase promotor; BSD-R: Blasticidin resistance; P2A: porcine teschovirus-1 2A co-translational cleavage sequence; X/E: XhoI/EcoRI: restriction enzyme cleavage sites. **D**: Flow cytometry-based percentage of fluorescent pTREBAV-shRpa3-218 or pTCEBAC-shRenilla transduced PyB6-TA cells over 17 days ± Dox treatment. Vectors used for transfection in equal ratios; antibiotic selection for 9 days prior experiment maintained during Dox treatment. Unt: untreated. **E**: Summary of established degradome-targeted cell pools for the synthetic lethality screen.

In follow up of our expression studies, the ultimate aim was to identify proteases whose depletion confers synthetic lethality upon simultaneous PI3K inhibition by a functional and unbiased approach. Thus, we generated inducible protease knockdown breast cancer cells by incorporating a customized degradome-focused miR-E library targeting 658 murine protease and protease-like transcripts with 4-7 miR-Es per target (Figure 2A). The murine PyB6-TA cells carry the reverse Tetracycline transactivator 3 (rtTA3) and were originally generated from primary breast cancers of the transgenic MMTV-PyMT metastatic breast cancer model 36,37. This model recapitulates the hyperactivation of the PI3K/mTOR axis observed in the majority of human breast cancer patients, making it especially suitable for investigating oncogenic signaling in breast cancer 10,37,38.

The degradome-focused miR-E library was subdivided into 16 pools, each of about 300 miR-Es, and was retrovirally transduced into PyB6-TA cells as two independent biological replicates. The transduction was optimized to yield a single-miR-E-copy per cell and to ensure integration of one miR-E into 1000 individual cells (1000-fold representation; Figure 2A). The miR-Es were cloned into a double-fluorescent vector system to allow for an inducible and traceable protease knockdown (Figure 2B). The pTREBAV vector constitutively expresses a Blasticidin resistance cassette together with a Venus fluorescent reporter, which allowed selection and monitoring of transduced cells. Further, the vector contains a Tetracycline responsible element (TRE) enabling for Dox-dependent expression of the miR-Es simultaneous with a fluorescent dsRed reporter. Constitutive Venus fluorescence was detectable in PyB6-TA cells by flow cytometry and treatment with Dox for three days clearly induced dsRed expression (Figure 2B). Since cell proliferation is dependent on the presence of replication protein A3 (Rpa3; 39), two shRNAs targeting Rpa3 (shRpa3-218/shRpa3-457) were used as positive “depletion” controls to proof correct function of the screen. A similar vector, pTCEBAC, with inducible Cyan and constitutive Cherry fluorescences was used to express shRNAs targeting Renilla Luciferase (shRenilla) or Firefly Luciferase (shLuciferase) transcripts which are not present in mammalian cells (Figure 2C). These shRNAs were used as negative “stability” controls to assess the quality of the screen and exclude negative effects of artificially expressed shRNAs on cell viability or proliferation due to a general interference with the RNAi pathway.

The controls were validated by using equal amounts of pTREBAV-shRpa3-218 and pTCEBAC-shRenilla DNA for transduction of PyB6-TA cells, following measurement of the percentage of constitutive and inducible fluorescent cells for 17 days (Figure 2D). As expected, PyB6-TA cells transduced with any of the vectors showed stable expression of the constitutive fluorescences. Upon Dox treatment the inducible dsRed fluorescence was expressed and the pTREBAV-shRpa3-218-transduced cells got subsequently depleted from the population. Because murine cells do not carry a *Renilla Luciferase* gene, its knockdown showed no effect and shRNA-induced pTCEBAC-transduced cells enriched in the cell culture. In the subsequent screens these internal stability and depletion controls were spiked into the miR-E library before transduction. As each of the 16 miR-E pools was transduced twice in independent experiments, this resulted in 32 degradome-targeted cell pools that were subjected to negative selection synthetic lethality screens (Figure 2E).

### Negative selection synthetic lethality screen

**Figure 3:**
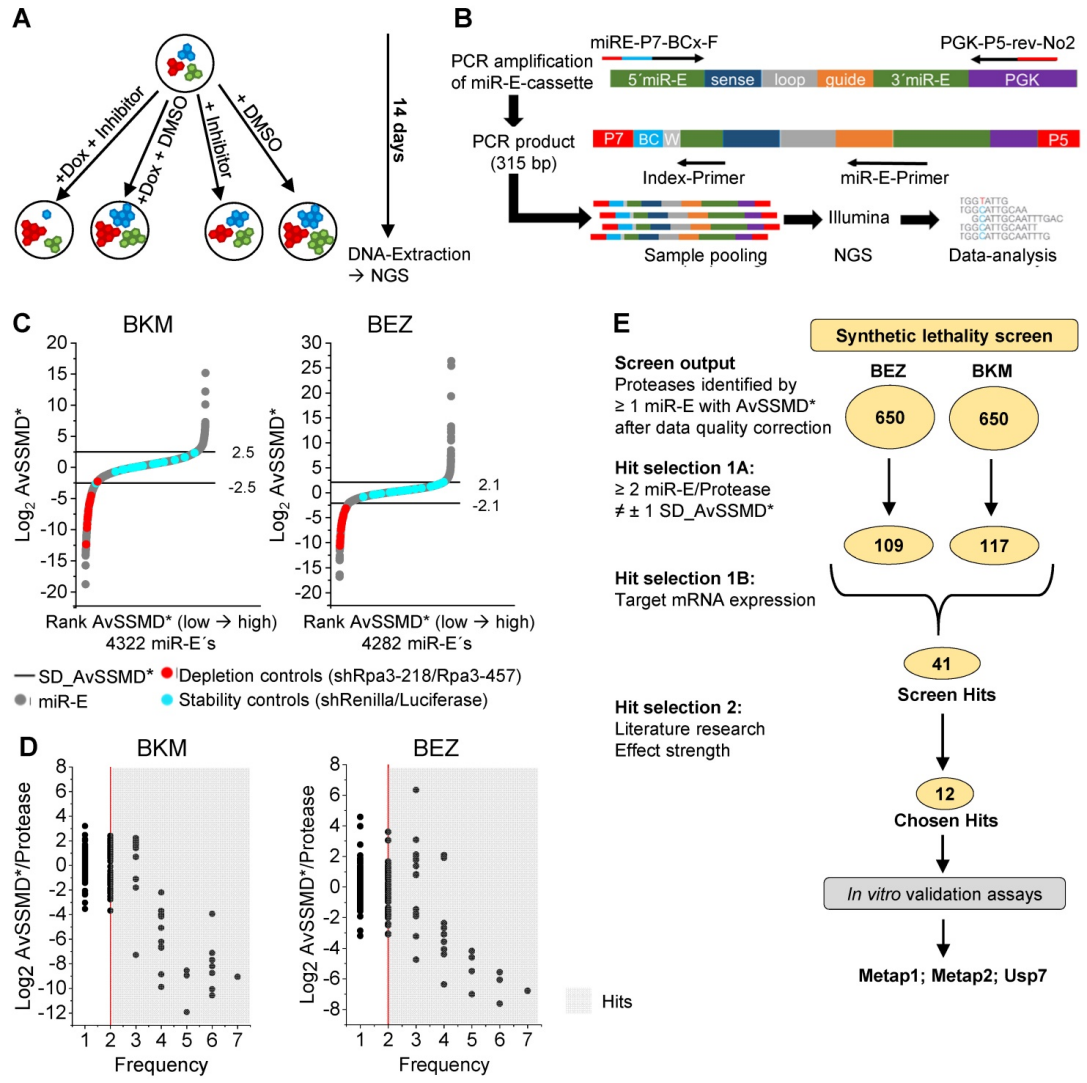
Negative selection synthetic lethality screen. **A**: Schematic setup of the synthetic lethality screen. Cells carrying different miR-E constructs in different colors. PI3K-inhibitor: BKM or BEZ at EC20. DMSO: solvent control. NGS: Next-generation sequencing. **B**: Schematic representation of miR-E-cassette PCR-amplification with flanking primers introducing P7/P5 adaptors (red) for Illumina sequencing and a sample specific barcodes (BCx; blue) following sample pooling and Illumina NGS with indicated primers. W: wobble. **C**: Distribution of Log2 AvSSMD* effect scores for all miR-E´s in the synthetic lethality screens. Rank AvSSMD*: values ranked by size. Grey: Log2 AvSSMD* score of one miR-E. Blue: shRenilla/shLuciferase stability controls. Red: shRpa3-218/shRpa3-457 depletion controls. Horizontal line: ± 1 SD_AvSSMD* of all miR-E´s in the screen. **D**: Dual flashlight plots. Black dot: Protease identified by minimum one corresponding miR-E with successfully calculated AvSSMD* after complete processing of sequencing reads. The term protease refers to all degradome-encoded proteins including proteases, protease-like proteins and protease subunits. Number of identified proteases per plot: 650. Hits highlighted in grey (≥ 2 miR-E/protease outside ± 1 SD_AvSSMD* [Frequency]). **E**: Summarized selection procedure with number of identified proteases in circles according to the criteria indicated. ≠: outside. In vitro validation assays: effect of knockdown induction on cell sensitivity to BKM. Searchable excel file with the screen output and hit selection 1A hits (screen hits and chosen hits highlighted) in Supplementary Table S2.

For screening of synthetic lethal combinations created by simultaneous PI3K inhibition and genetic protease depletion, the 32 degradome-targeted PyB6-TA cell pools were treated with two PI3K pathway inhibitors (BEZ; BKM), or DMSO as solvent control, in the presence or absence of Dox (Figure 3A). PI3K-inhibitors were used at EC20 concentrations to cause only minor perturbation of cell growth. Genomic DNA was extracted after 14 days in culture and the miR-E cassette was amplified using flanking primers, which introduced standard Illumina adapters and a sample specific 10 nucleotide barcode sequence into the amplicon (Figure 3B). Samples were subjected to Illumina NextSeq500 DNA sequencing resulting in the number of reads for each miR-E per condition. The normalized Log2 transformed number of reads was used for calculation of the robust strictly standardized median difference (AvSSMD*) as measure of effect size 40. In our setting, the AvSSMD* represented the difference in abundance of a specific miR-E construct in double treated (BEZ + Dox or BKM + Dox) cells to PI3K-inhibitor-only and DMSO-only treated samples. Hence, proteases targeted by miR-Es with negative AvSSMD* scores dropped out of the inhibitor + Dox treated population indicating synthetic lethality with the PI3K-inhibitor. The quality of the screen data was evaluated by the overall distribution of AvSSMD* scores for all miR-Es recovered in the screen (Figure 3C).

The majority of miR-Es scored around zero, thereby indicating no significant effect of Dox-mediated protease targeting with combined PI3K-inhibitor treatment on relative miR-E representation and hence breast cancer cell viability or growth. The intrinsic variability of the screen was defined as one standard deviation from the AvSSMD* of all miR-E constructs contained in the screen (SD_AvSSMD*) and was used as cutoff for effect strength (Figure 3C). As expected, shRNAs targeting Renilla or Luciferase (blue circles; Figure 3C) scored inside ± 1 SD_AvSSMD* of the data. Substantial interaction of protease-targeting and PI3K inhibition was indicated by an effect outside the boundaries of ± 1 SD_AvSSMD*. Notably, the depletion controls (red circles, Figure 3C) targeting the essential Rpa3 were strongly depleted in both screens, thereby indicating correct screen performance. Because each protease transcript was targeted by multiple miR-Es, the Log2 AvSSMD* per protease was plotted against the number of miR-Es targeting this transcript that scored outside ± 1 SD_AvSSMD* (Figure 3D). Only proteases targeted by a minimum of two independent miR-Es (frequency ≥ 2) were considered as hits (grey background; Figure 3D); a pre-selection procedure previously applied by others 41-43. For these hits combined protease targeting and PI3K inhibition led to negative AvSSMD*/protease scores that indicate synthetic lethality. Positive scores indicate possible growth promoting effects caused by the combination of PI3K-inhibitors and depletion of the indicated protease. Of the 658 proteases targeted by the miR-E-library, miR-Es corresponding to an equal number of 650 proteases could be identified in the sequencing output of either screen (Figure 3E). Because hit selection was restricted to targets with at least two miR-Es outside ± 1 SD_AvSSMD*, 109 hits were identified in the BEZ synthetic lethality screen and 117 hits in the BKM screen. Combination of the hits from both screens (181 hits) and subsequent STRING-based protein association network analysis revealed that the biggest hit cluster contained proteasomal subunits and Deubiquitinases ([DUBs]; Supplementary Figure S1). This highlights the well-established importance of a functioning ubiquitin-proteasome system (UPS) for general cell survival and proliferation 44-47. The detection of several subunits of the proteasome, including all 7 Psma and Psmb subunits of the 20S catalytic core particle, as hits with strongly negative AvSSMD* scores was a proof-of-principle finding that further validates our screening approach.

As low target mRNA expression increases the likelihood for false positive hits 48, filtering the screen hits for reasonably high miR-E target mRNA expression (data on PyB6-313 cells; fragments per kilo base per million mapped reads [FPKM] ≥ 0.5) led to 41 remaining protease candidates (screen hits; Supplementary Table S2C/D highlighted in blue). Those were further subjected to literature review as well as to comparison of effect strength and outcome between the two screens. We also took our RNA-Seq data into consideration, including proteases like OTUD5, which was significantly upregulated in all three breast cancer cell lines, or Cathepsin A that was significantly upregulated in MCF7 and MDA-MB-231 cells. However, effect strength and target mRNA expression were most important for our selection process. For 12 of those hits (chosen hits; Supplementary Table S2C/D highlighted by *) inducible knockdown constructs were introduced into PyB6-TA breast cancer cells; each with two miR-Es per protease. Final candidate selection was based on literature data as well as on effect strength and data variability of *in vitro* validation assays, which tested the effect of knockdown induction on the sensitivity to BKM treatment in MTT assays (Supplementary Figure S2). By these means we identified the Methionine aminopeptidases 1 and 2 (Metap1; Metap2) as well as the Ubiquitin carboxyl-terminal hydrolase 7 (Usp7) as top candidates for more detailed exploration. Interestingly, significantly reduced Metap1 mRNA expression was observed upon PI3K-inhibitor treatment of PyB6-TA cells with BEZ (EC20/EC50) or BKM (EC20) for 4 days (Supplementary Figure S3A) in line with our previous RNA-Seq experiment upon short term (3 h) BKM treatment (Figure 1). Additionally, comparable to the RNA-Seq data, significant downregulation of USP7 expression was observed in human rtTA3-transduced MDA-MB-231 (MDAMB-TA) cells upon BEZ EC20 treatment as well as in PyB6-TA cells upon BEZ EC50 treatment (Supplementary Figure S3C). Furthermore, a significant downregulation of Metap2 was observed upon BKM EC20 treatment in PyB6-TA cells (Supplementary Figure S3B). These small but significant changes in Metap1, Metap2 and Usp7 mRNA expression upon PI3K inhibition were further reason to study these three proteases as potential synthetic lethal partners of PI3K inhibition.

### Knockdown of Metap1, Metap2 or Usp7 sensitizes murine breast cancer cells to PI3K inhibition

**Figure 4:**
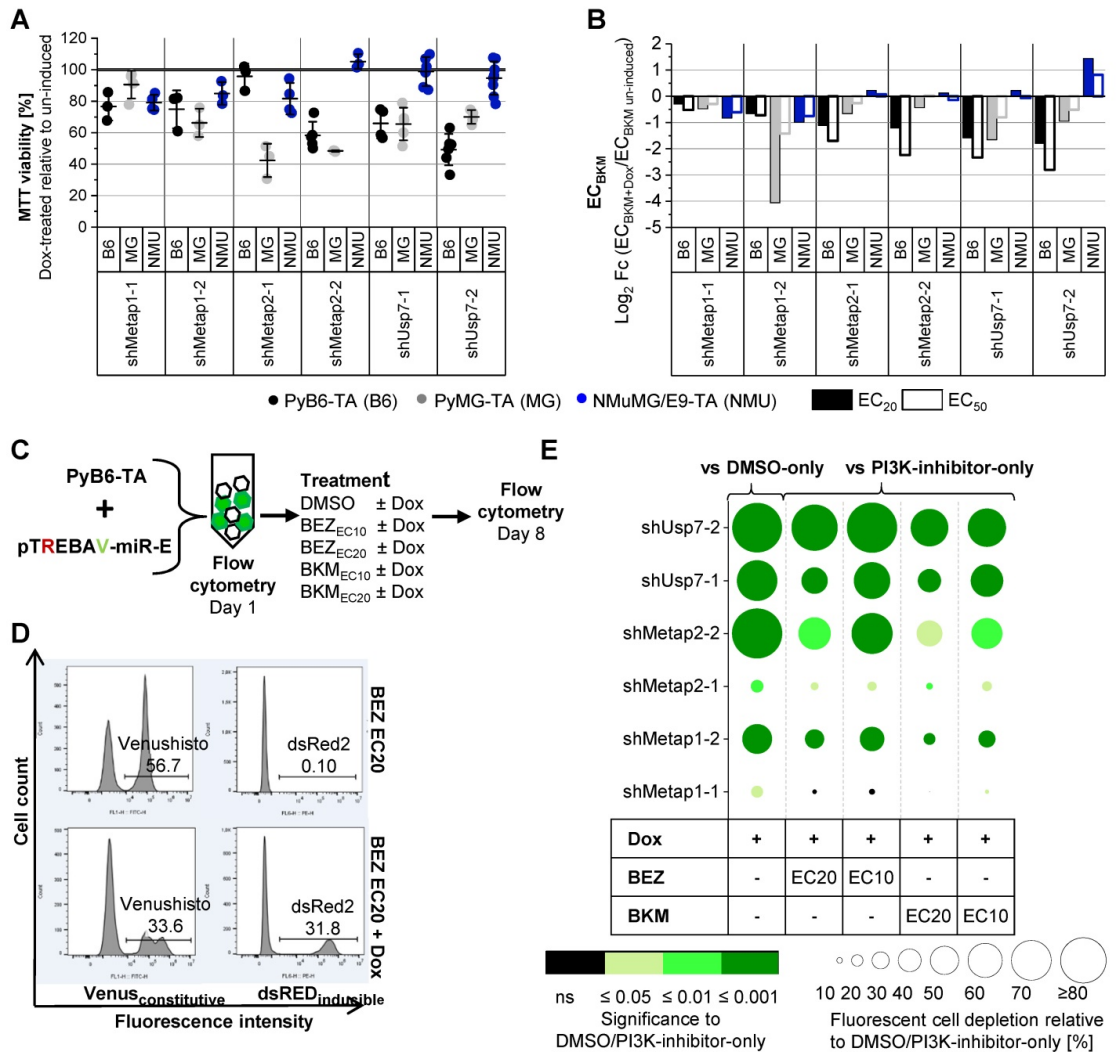
Metap1, Metap2 or Usp7 knockdown sensitize murine breast cancer cells to PI3K inhibition. **A**: MTT viability as mean ± SD (n ≥ 3) of Dox treated (total 5 days) to uninduced, untreated controls of the BKM MTT viability assays in B. **B**: Log2 fold change (Fc) of BKM EC20 and EC50 values of protease targeted (Dox treated) PyB6-TA (B6), PyMG-TA (MG) or NMuMG/E9-TA (NMU) cells relative to uninduced cells based on MTT viability assay dose-response curves. Data presented as fold change of EC means (n ≥ 3). MiR-E expression was pre-induced for 3 days prior inhibitor treatment for 48 h. EC: effective concentration. **C-E**: Competitive growth flow cytometry assays. **C**: General setup. Flow cytometry-based analysis of competitive cell growth upon combined protease targeting and PI3K inhibition in miR-E-transduced PyB6-TA breast cancer cells mixed with non-fluorescent PyB6-TA controls cells. Cells cultured for 8 days treated as indicated. **D**: Representative flow cytometry histograms for miR-E-Usp7-2-transduced PyB6-TA cells mixed with non-fluorescent PyB6-TA cells treated with BEZ EC20 ± Dox. Fluorescent cells from living, single cells. Gates in %. **E**: Effect of combined protease targeting and PI3K inhibition on competitive cell growth. Circle radius: Mean depletion of fluorescent cells relative to DMSO-only or PI3K-inhibitor-only treated cells in %. Circle color: significance of circle radius; one-sample t-test to 0 (n = 5-6). Table with all mean values ± SD in Supplementary Table S3. For simplification miR-Es are indicated by sh. Note that the EC values were determined in 48 h MTT assays.

A potent knockdown of Metap1, Metap2 and Usp7 was achieved after 4 and 8 days of Dox treatment in PyB6-TA cells (Supplementary Figure S4). To further address the cooperation of protease targeting and PI3K inhibition, two inducible Metap1, Metap2 or Usp7 knockdown breast cancer cell lines (PyB6-TA, PyMG-TA) and one non-cancerous normal murine mammary gland cell line (NMuMG/E9-TA) were used. Knockdown of Usp7, Metap1 or Metap2 impaired MTT viability to different extent, with both breast cancer cell lines being more sensitive than non-cancerous cells (Figure 4A). Knockdown of Metap2 in PyMG-TA cells showed the strongest effect, reducing MTT viability by more than 50%. Additionally, Metap1, Metap2 or Usp7 knockdown sensitized PyMG-TA and PyB6-TA breast cancer cells to BKM treatment in comparison to cells with unaltered protease expression (Figure 4B). Importantly, little sensitization was observed in NMuMG/E9-TA cells. This difference in susceptibility between breast cancer cells and non-cancerous cells was most prominent upon knockdown of Metap2 and Usp7. Before functional analysis of human protease knockdown cell lines, competitive growth assays were performed with PyB6-TA cells (Figure 4C-E). Knockdown of any protease in fluorescent pTREBAV-miR-E-PyB6-TA cells seeded together with non-targeted, non-fluorescent PyB6-TA cells for 8 days significantly depleted the knockdown cells in the population relative to DMSO-only treated cells (Figure 4E). Depletion of knockdown cells was most prominent upon induction of miR-Es Usp7-2 and Metap2-2 with over 70% fluorescent cell depletion. Combined protease targeting with BEZ or BKM (EC10 or EC20) treatment further reduced the percentage of fluorescent knockdown PyB6-TA cells in the population in comparison to PI3K-inhibitor-only treated cells, with most significant effects for combined induction of miR-E-Usp7-2 and BEZ EC10 treatment. These data highlight the advantage of simultaneous protease/kinase targeting over the single use of the PI3K-inhibitors.

### Knockdown of Metap1 or Metap2 mediates combinatory and partly synergistic effects with PI3K inhibition in human and murine cancer cells

**Figure 5:**
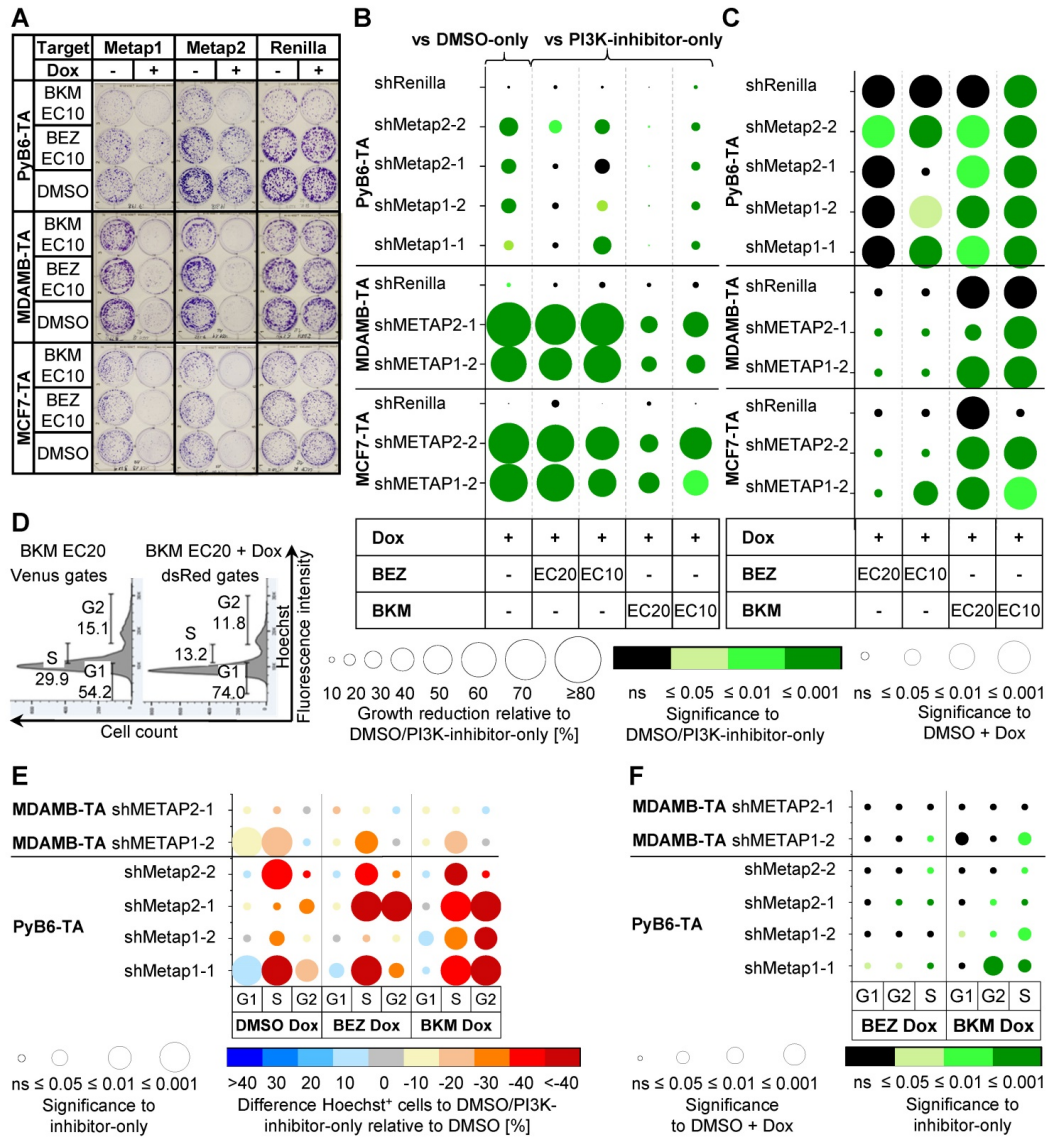
Effects of Metap1/2 knockdown and PI3K inhibition in human and murine breast cancer cells. **A-C**: Crystal violet plate colony formation assays. Cell lines and treatment as indicated for 8-10 days (murine) or 10-12 days (human). ShRenilla cells were used as control. **A**: Representative pictures, picture contrast +20% in power point. Renilla same pictures as in Figure 6. **B**: Relative changes in colony growth between double treatment and single treatment (n = 5-10; n = 10-19 [DMSO + Dox pooled from EC10 and EC20]). Circle radius: Mean colony growth reduction relative to DMSO-only or PI3K-inhibitor-only treated cells normalized to DMSO in %. Circle color: Significance of circle radius; two-sample t-test; non-equal variances assumed. In case of DMSO-Dox treated cells one-sample t-test to 0. **C**: Significance of growth reduction upon double treatment compared to single PI3K-inhibitor treatment or knockdown alone to detect synergism. Circle radius: Significance in growth reduction between PI3K-inhibitor-Dox treated and DMSO-Dox treated cells relative to DMSO; two-sample t-test; non-equal variances assumed. Circle color: see B. Table with all mean values ± SD in Supplementary Table S4. **D-F**: Flow cytometry-based analysis of cell cycle phase distribution in cells treated with PI3K pathway-inhibitors ± Dox as indicated. Inhibitor treatment at EC20 for 12 h (PyB6-TA) or 24 h (MDAMB-TA) on serum starved cells (n = 4-8). **D**: Representative histograms for miR-E-Metap1-1-transduced PyB6-TA cells treated with BKM EC20 ± Dox. Hoechst+ cells from living, single cells first gated for inducible (Venus-dsRed; Dox treated cells) or constitutive (Venus; non-Dox treated cells) fluorescent cells. Gates in %. **E**: Difference in cell cycle phase distribution between double treated and PI3K-inhibitor-only treated cells relative to DMSO. Circle color: Mean difference in Hoechst positive (Hoechst+) cells relative to DMSO-only or PI3K-inhibitor-only treated cells normalized to DMSO in %. Circle radius: Significance of circle color; two-sample t-test; non-equal variances assumed. In case of DMSO-Dox treated cells one-sample t-test to 0. **F**: Significance of double treatment compared to single PI3K-inhibitor treatment or knockdown alone. Circle radius: Significance of difference in Hoechst+ cells between PI3K-inhibitor-Dox treated and DMSO-Dox treated cells relative to DMSO; two-sample t-test; non-equal variances assumed. Circle color: Significance between PI3K-inhibitor-Dox treated to PI3K-inhibitor-only treated cell; two-sample t-test; non-equal variances assumed. Table with all mean values in Supplementary Table S5 G1/S/G2: respective cell cycle phase. For simplification miR-E´s are indicated by sh. Note that the EC values were determined in 48 h MTT assays.

Next, we tested whether knockdown of Metap1 or Metap2 might provide synergistic effects to PI3K inhibition not only in murine breast cancer cells, but also in human MCF7-TA and MDAMB-TA cells representing luminal A-type and triple-negative breast cancers, respectively. For human breast cancer cells, experiments were performed with the miR-E that generated the most potent protein reduction after 4 and 8 days of Dox treatment (Supplementary Figures S5-S6). In colony formation assays, miR-E-transduced human and murine breast cancer cells were treated with BEZ or BKM (EC10 or EC20) alone or in combination with protease targeting (Figure 5A-C). Knockdown of Metap1 or Metap2 alone already significantly impaired breast cancer cell growth in comparison to DMSO-only treated cells (Figure 5B). Importantly, combined protease knockdown and PI3K-inhibitor treatment additionally impaired cell growth compared to the use of the PI3K-inhibitors alone, especially in human breast cancer cells and upon use of EC10. In contrast, ShRenilla-transduced control cells showed no considerable effect. Applying an effect-based definition 49, synergism was considered when the effect of combinatory treatment (PI3K-inhibitor-Dox treatment) was significantly better than the effects of the individual components (PI3K-inhibitor-only and DMSO-Dox treatment). Hence, in Figure 5C synergism is indicated by significantly stronger growth reduction in PI3K-inhibitor-Dox treated cells compared to PI3K-inhibitor-only treated cells (green circle color) and DMSO-Dox treated cells (circle radius p ≤ 0.05; Figure 5C). Synergistic effects on breast cancer cell growth were observed upon knockdown of Metap1 or Metap2 in combination with BKM EC10 and EC20 treatment in all three cell lines. In murine breast cancer cells synergisms was also detected for combined Metap1 or Metap2 knockdown and BEZ treatment, despite for shMetap2-1. In human breast cancer cells METAP1 or METAP2 knockdown in combination with BEZ treatment reduced cell growth significantly better than knockdown alone but not significantly better than PI3K-inhibitor treatment alone, comparable with shRenilla control cells (Figure 5C). The statistical significance, however, does not directly indicate effect size, which is depicted in Supplementary Table S4.

We further set out to investigate whether the synergistic effect of PI3K inhibition and Metap1 or Metap2 knockdown is transferrable to cancer cells not originating from breast malignancies. Introduction of inducible knockdown constructs into human colorectal adenocarcinoma ([CRC]; Caco2-TA, LoVo-TA) as well as human hepatocellular carcinoma ([HCC]; HuH7-TA, HEP-3B-TA) cell lines yielded in a potent knockdown of METAP1 or METAP2 after 4 and 8 days of Dox treatment (Supplementary Figures S7A/B and S8A/B). Cancer cells transduced with the more potent of the two miR-E construct per protease were subjected to flow cytometry based competitive growth assays (Supplementary Figure S9). Relative to DMSO-only treated cells, knockdown of METAP1 or METAP2 in fluorescent pTREBAV-miR-E-transduced CRC and HCC cells depleted the knockdown cells in the population only to minor extent (mostly < 20%). The strongest effect (27% reduction) was observed upon knockdown of METAP1 in HuH7-TA cells. Combined protease targeting and PI3K inhibition by BEZ or BKM further reduced the percentage of fluorescent knockdown cells in the population for many combinations (Supplementary Figure S9, indicated by a green circle color). These combinatory effects were statistically significant but rather small (mostly < 20%), especially when compared to the impressive combinatory effects observed in PyB6-TA breast cancer cells (Figure 4E). Combinatory effects of METAP1 or METAP2 knockdown and PI3K inhibition in HCC and CRC cells were more evident in colony formation assays (Supplementary Figures S10-S11). Knockdown of METAP1 or METAP2 with simultaneous PI3K inhibition by BEZ or BKM (EC10/EC20) led to an additional significant reduction in colony formation compared to PI3K inhibition alone. The strength of this additional combinatorial effect (Supplementary Figures S10-S11; red bars) was thereby comparable to human breast cancer cells and was strongest for HEP-3B-TA cells. Although the protease knockdown itself already affected HCC and CRC cells (Supplementary Table S4), synergistic effects of co-treatment were discovered in many instances. This was for example evident upon METAP1 knockdown and simultaneous BKM EC10 or EC20 treatment in Caco2-TA cells (Supplementary Figures S10). Synergistic effects were defined by a statistically significant growth reduction of simultaneous protease/PI3K targeting compared to the PI3K-inhibitor alone (black *) and the knockdown alone (grey *; Supplementary Figures S10-S11). Notably, in human breast cancer cells the overall effect of the combinatory treatment was strongest leading to over 90% reduction in colony growth. Surprisingly, upon BEZ single treatment in HCC and CRC cells an increased colony growth was observed. This was reversed upon combined METAP knockdown leading to an overestimation of the relative effect of the co-treatment.

### Effect of combinatory Metap/PI3K interference on PI3K signal transduction and cell cycle

To investigate possible mechanisms causing the combinatory effects of simultaneous Metap/PI3K inhibition in breast cancer cells, cell cycle phase distribution was investigated by DNA content determination (Figure 5D-F). In comparison to DMSO-only treated cells, knockdown of Metap1 or Metap2 stalled murine PyB6-TA cells in G1-phase and human MDAMB-TA cells in G2-phase, thus leading to an impaired cell cycle progression to S-phase in murine and human breast cancer cells (Figure 5E). Combined PI3K inhibition and protease knockdown additionally impaired cell cycle progression compared to PI3K-inhibitor-only treated cells relative to DMSO. Thereby, cell cycle impairment was in most cases significantly stronger upon double treatment compared to using the PI3K-inhibitors alone (Figure 5F, green circles). Consequently, quantitative RT-PCR based analysis of three transcriptionally regulated cell cycle inhibitors (p21, p27, p57) and one apoptosis mediator (Puma) was performed in PyB6-TA cells, but revealed no significant treatment-dependent change in mRNA expression (Supplementary Figure S12). In human MCF7-TA and MDAMB-TA cells analysis of the cell cycle regulators p21 and cyclin D1 mRNA expression also showed no consistent differences between combinatory and single treatments (Supplementary Figure S13). Further, no significant changes in the phosphorylation status of 17 proteins related to mitogen activated protein kinase (MAPK) signaling were detected in human MCF7-TA or MDAMB-TA cells between BKM treatments alone or in combination with protease knockdown (Supplementary Figure S14). However, upregulated inhibitory phosphorylation of eukaryotic initiation factor 2 subunit alpha (eIF2α), important for global protein and DNA synthesis 50,51, was observed upon knockdown of Metap1 and Metap2 in PyB6-TA cells (Supplementary Figure S15).

Because the synergistic effects might be due to a direct link of METAPs to PI3K signaling, we analyzed the activity of the PI3K pathway by means of the phosphorylation status of three players (Akt, mTOR, S6) upon METAP1 or METAP2 knockdown in human breast cancer cells (Supplementary Figure S16). Knockdown of METAP1 significantly reduced the ratio of Serine(Ser)2448 phosphorylated mTOR (pmTOR) to total mTOR by 30% as well as the ratio of phosphorylated S6 (pS6; Ser235/236) to S6 by 18% in MDAMB-TA cells. In contrast, no significant changes in PI3K pathway activation were observed in MCF7-TA cells. Upon knockdown of METAP2 significantly reduced levels of Ser473 phosphorylated Akt (pAkt; 16% reduction) were observed in MDAMB-TA cells whereby in MCF7-TA cells significantly reduced pmTOR (17% reduction) and pS6 (13% reduction) levels were detected.

In summary, knockdown of Metap1 or Metap2 sensitized murine and human breast cancer cells as well as human hepatocellular carcinoma and colorectal cancer to PI3K inhibition showing partly synergistic effects. Furthermore, an impaired cell cycle progression in murine PyB6-TA and human MDAMB-TA cells was observed connected to increased inhibitory phosphorylation of eIF2α in murine PyB6-TA cells. In addition, small but significant changes in PI3K pathway activation were observed upon knockdown of METAP1 or METAP2 in human breast cancer cells.

### Usp7 depletion results in combinatory and partly synergistic effects with PI3K inhibition in human and murine cancer cells

**Figure 6:**
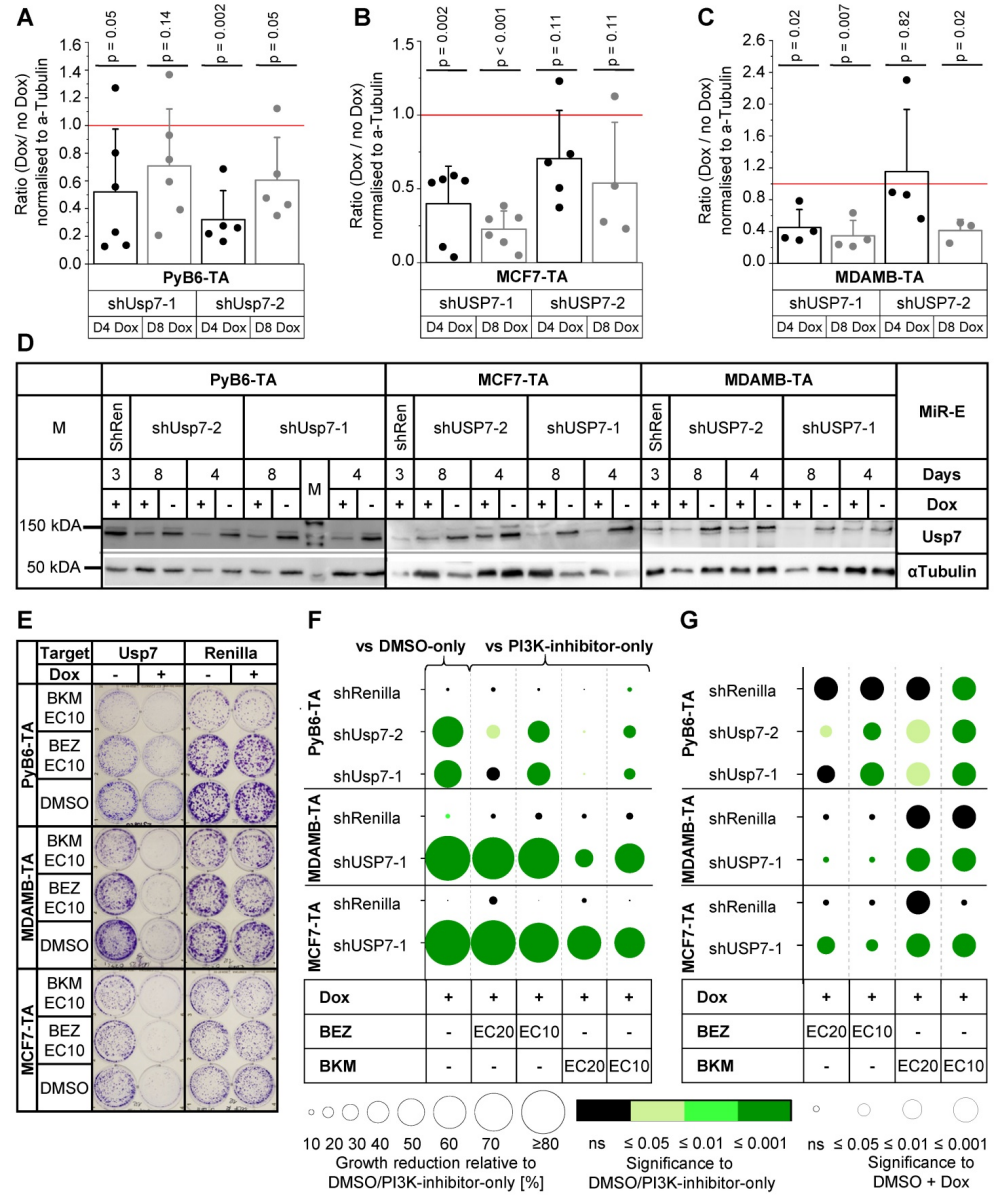
Effects of Usp7 knockdown and PI3K inhibition in human and murine breast cancer cells. **A-D**: Analysis of Usp7 protein expression by Western blot in miR-E-transduced PyB6-TA, MCF7-TA and MDAMB-TA cells cultured for 4 days (D4) or 8 days (D8) ± Dox treatment. **A-C**: Quantification of Western blot data; protein level as mean ratio + SD between Dox-treated miR-E-induced and uninduced cells normalized to α-Tubulin. Significance (p): one-sample t-test to 1 (A: n = 5-6, B: n = 4-6, C: n = 3-4). Red line: no change in protein level between miR-E induced and uninduced. **D**: Representative Western blots; α-Tubulin as loading control; 25 µg (PyB6-TA) or 50 µg protein loaded. M: marker. shRen: ShRenilla cells as control. **E-G**: Crystal violet plate colony formation assays. Cell lines and treatment as indicated for 8-10 days. ShRenilla cells were used as control. **E**: Representative pictures, picture contrast +20% in power point. Renilla same pictures as in Figure 5. **F**: Relative changes in colony growth between double treatment and single treatment (n = 5-8; n = 10-15 [DMSO + Dox pooled from EC10 and EC20]). Circle radius: Mean colony growth reduction relative to DMSO-only or PI3K-inhibitor-only treated cells normalized to DMSO in %. Circle color: Significance of circle radius; two-sample t-test; non-equal variances assumed. In case of DMSO-Dox treated cells one-sample t-test to 0. **G**: Significance of growth reduction upon double treatment compared to single PI3K-inhibitor treatment or knockdown alone to detect synergism. Circle radius: Significance in growth reduction between PI3K-inhibitor-Dox treated and DMSO-Dox treated cells relative to DMSO; two-sample t-test; non-equal variances assumed. Circle color: see B. Table all mean values ± SD in Supplementary Table S4. For or simplification miR-Es are indicated by sh. Note that the EC values were determined in 48 h MTT assays.

Synergistic effects of combined Usp7 knockdown and PI3K inhibition were analyzed in murine PyB6-TA as well as human MCF7-TA and MDAMB-TA breast cancer cells, using the miR-E-transduced cell lines that generated the most potent protein reduction after 4 and 8 days of Dox treatment (Figures 6A-D). Colony formation assays revealed potent, significantly impaired cell growth of DMSO-Dox treated Usp7 knockdown cells in comparison to DMSO-only treated cells in all three cell lines (Figure 6E-F). Combination of Usp7 knockdown and PI3K inhibition showed an additional cell growth impairment compared to PI3K-inhibitor-only treated cells; an effect that was most prominent in human breast cancer cells and upon use of PI3K-inhibitors at EC10 (Figure 6F). Synergistic effects on breast cancer cell growth were observed upon combined Usp7 knockdown and BKM EC10 and EC20 treatment in all three cell lines, indicated by a significantly stronger growth reduction in PI3K-inhibitor-Dox treated cells compared to PI3K-inhibitor-only treated cells (green circles) and DMSO-Dox treated cells (circle radius p ≤ 0.05; Figure 6G). Combination of Usp7 knockdown and BEZ treatment reduced colony growth significantly better than the PI3K-inhibitor alone, which was synergistic in many cases. The actual effect sizes are presented in Supplementary Table S4.

We further investigated whether the synthetic lethal effect of combined PI3K inhibition and genetic Usp7 targeting could be reproduced using Usp7-inhibitors. MTT based dose-response curves were performed with two different non-covalent Usp7-inhibitors (GNE6776 52, P005091 53) in miR-E-Renilla transduced MDAMB-TA, MCF7-TA and PyB6-TA cells (Supplementary Figures S17-S18). Due to a higher potency of P005091 further experiments were performed with this compound. Combinatory treatment of human and murine breast cancer cells with P005091 and the PI3K-inhibitors BEZ and BKM showed synergistic effects in colony formation assays (Supplementary Figures S19-S20). For most concentrations, combinatory treatment reduced colony growth significantly better than at least one of the inhibitors (Supplementary Figures S19C-S20C/E). Synergistic effects, defined by significantly stronger reduction of colony growth upon double treatment compared to single use of any of the inhibitors, were observed especially at higher EC values (EC50) and the use of BKM (Supplementary Figures S19-S20; green squares).

To investigate whether USP7 knockdown could also sensitize cells from other cancer entities to PI3K pathway inhibition, we utilized the human CRC (Caco2-TA, LoVo-TA) and HCC (HEP-3B; HuH7-TA) cell lines harboring the constructs with strongest USP7 knockdown after 4 and 8 days of Dox treatment (Supplementary Figures S7C-S8C). FACS-based competitive growth assays showed significant reduction of fluorescent USP7 knockdown cells compared to DMSO-only treated cells, which was strongest for HuH7-TA cells (43% reduction; Supplementary Figure S9C). In HuH7-TA and Caco2-TA cells combined USP7 knockdown and PI3K inhibition by BEZ or BKM (EC10/EC20) led to significantly less fluorescent cells compared to the use of PI3K-inhibitors only, but the effect size was rather small (< 30%) with the exception of BEZ EC10 treatment in HuH7-TA cells (45% reduction). Significant combinatory effects of USP7 knockdown and PI3K inhibition were also observed in colony formation assays with all HCC and CRC cell lines (Supplementary Figure S21). Although the overall effect of the combinatory treatment was strongest in human breast cancer cells with more than 90% reduction in colony growth, the strength of the additional growth reduction between combinatory targeting and single PI3K-inhibitor treatment in HCC and CRC cells was comparable to human breast cancer cells (red bars; Supplementary Figure S21). Synergistic impairment of colony growth was observed upon various PI3K-inhibitor/USP7 knockdown combinations compared to the PI3K-inhibitor alone (black *) and the USP7 knockdown alone (grey *, Supplementary Figure S21), while the effect of USP7 knockdown can be found in Supplementary Table S4. As already observed with the miR-E METAP transduced cells, BEZ single treatment increases colony growth of HCC and CRC cells leading to a relative overestimation of colony growth impairment upon combinatory treatment (Supplementary Figure S21).

### Effect of combinatory Usp7/PI3K interference on PI3K signal transduction and cell cycle

**Figure 7:**
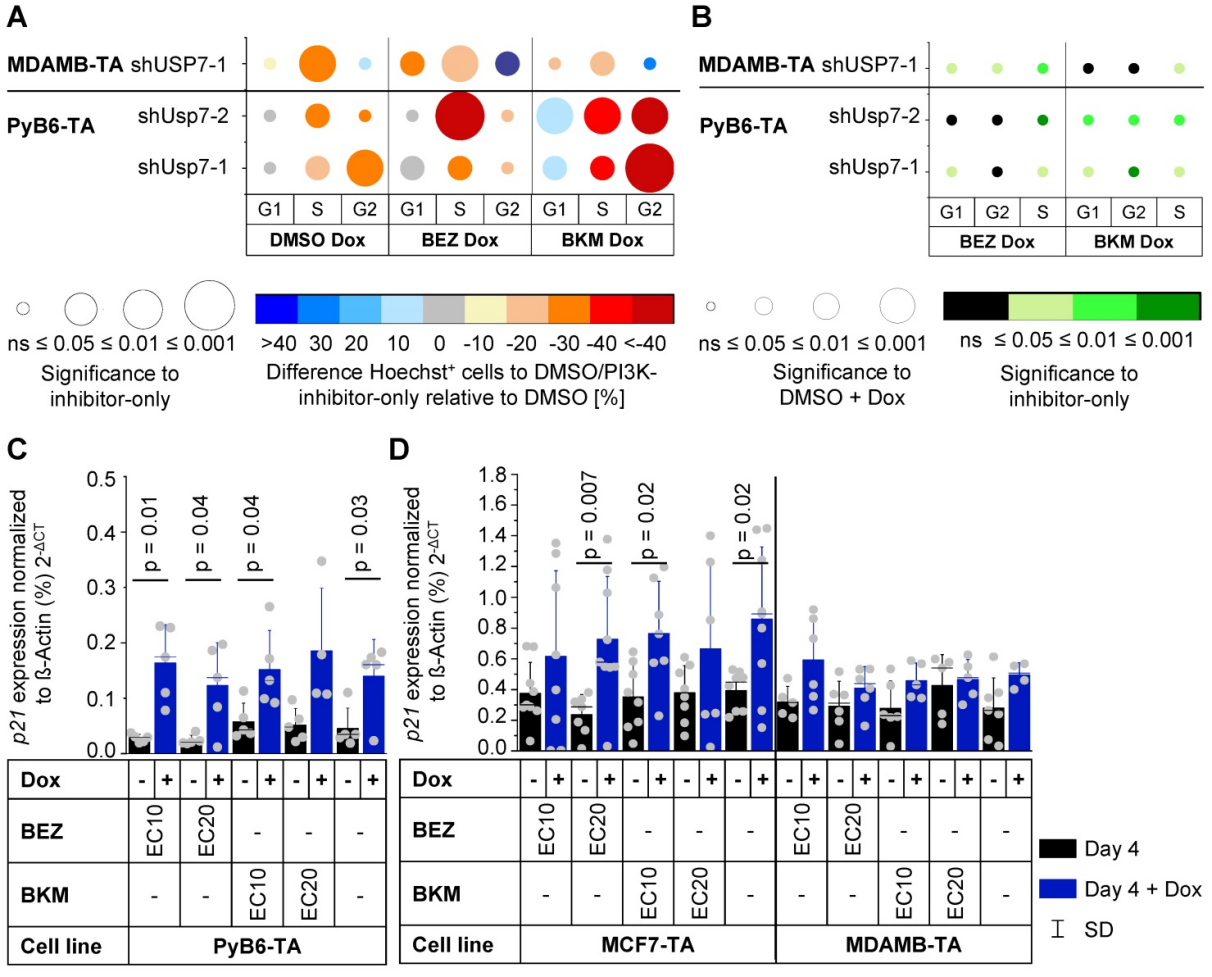
Cell cycle impairment upon Usp7 knockdown and PI3K inhibition in human and murine breast cancer cells. **A-B**: Hoechst 33342 staining-based cell cycle phase distribution analysis of cells treated with PI3K pathway-inhibitors ± Dox analyzed by flow cytometry. Inhibitors and cell lines as indicated. Inhibitor treatment at EC20 for 12 h (PyB6-TA) or 24 h (MDAMB-TA) on serum starved cells (n = 4-8). Representative flow cytometry histograms see Figure 5D. For simplification miR-Es are indicated by sh. **A**: Difference in cell cycle phase distribution between double treated and PI3K-inhibitor-only treated cells relative to DMSO. Circle color: Mean difference in Hoechst positive (Hoechst +) cells relative to DMSO-only or PI3K-inhibitor-only treated cells normalized to DMSO in %. Circle radius: Significance of circle color; two-sample t-test; non-equal variances assumed. In case of DMSO-Dox treated cells one-sample t-test to 0. **B**: Significance of double treatment compared to single PI3K-inhibitor treatment or knockdown alone on cell cycle distribution. Circle radius: Significance of difference in Hoechst+ cells between PI3K-inhibitor-Dox treated and DMSO-Dox treated cells relative to DMSO; two-sample t-test; non-equal variances assumed. Circle color: Significance between PI3K-inhibitor Dox treated to PI3K-inhibitor-only treated cell; two-sample t-test; non-equal variances assumed. Table with all mean values in Supplementary Tables S4. G1/S/G2: respective cell cycle phase. **C-D**: RT-PCR based p21 mRNA expression in cells cultured for 4 days ± Dox in combination with PI3K pathway-inhibitors BEZ or BKM at EC10 or EC20. DMSO as solvent control. Cell lines as indicated. MRNA expression as mean ± SD percentage of ß-Actin; Significance (p): two-sample t-test of PI3K-inhibitor-Dox treated to PI3K-inhibitor-only treated; non-equal variances assumed; or two-sample t-test of PI3K-inhibitor-Dox treated conditions to DMSO-Dox treated samples (n = 4-5 [PyB6-TA]; n = 6-9 [MDMAB-TA]; n = 5-7 [MCF7-TA]).

Cell cycle analysis revealed that knockdown of Usp7 alone stalled murine PyB6-TA cells in G1 cell cycle phase leading to a reduced number of cells in G2- and S-phase (Figure 7A). USP7 knockdown in human MDAMB-TA cells stalled cell cycle in G2-phase, reducing cells in G1- and S-phases. Combination of Usp7 knockdown and PI3K-inhibitor treatment showed a similar pattern of cell cycle impairment. Interestingly, depletion of human and murine breast cancer cells from S-phase was significantly better in combinatory targeted cells than when using the respective PI3K-inhibitors alone (Figure 7B). Additionally, Usp7 knockdown significantly increased mRNA expression of the cell cycle inhibitor p21 in murine PyB6-TA and human MCF7-TA breast cancer cells (Figure 7C/D). In contrast, in MDAMB-TA cells no p21 mRNA induction was observed, which is likely explained by their p53 mutant status (see discussion). Messenger RNA expression of the cell cycle inhibitors p27 and p57 as well as the apoptosis mediator Puma were unaffected by Usp7 knockdown alone or in combination with PI3K inhibition in PyB6-TA cells (Supplementary Figure S12). Furthermore, cyclin D1 expression showed no changes in MDAMB-TA and MCF7-TA cells (Supplementary Figure S13). To study a possible connection of Usp7 to PI3K signaling, we examined the activity of the PI3K pathway by phosphorylation status of three players (Akt, mTOR, S6) upon USP7 knockdown in human breast cancer cells (Supplementary Figure S16). USP7 knockdown significantly reduced pAkt levels in MDMAB-TA cells by 22% as well as pmTOR levels in MCF-TA cells by 19%.

Taken together, Usp7 knockdown or pharmacological inhibition in human and murine breast cancer cells mediated combinatory and partly synergistic effects to PI3K inhibition that were transferrable to human hepatocellular carcinoma and colorectal cancer cell lines. Further, a slightly but significantly reduced PI3K pathway activity was observed upon USP7 knockdown in human breast cancer cells and impaired cell cycle progression was observed in murine and human Usp7 knockdown breast cancer cells. In line, increased p21 mRNA expression was found in PyB6-TA and MCF7-TA but not in MDAMB-TA cells.

## Discussion

The PI3K/mTOR axis is frequently activated in breast cancer 9,10,12. Nevertheless, PI3K-inhibitors have a low therapeutic index in this cancer entity 3,5,13,15, making combinatory therapy essential to fight this disease 17. Here, we propose proteases as potential new combinatory targets and synthetic lethal partners to PI3K inhibition in breast cancer. Initial expression studies revealed significantly altered protease transcript levels upon short-term PI3K inhibition in human and murine breast cancer cells (Figure 1), pointing to a yet ill-defined interplay between proteases and the PI3K pathway. For unbiased identification of proteases that sensitize murine breast cancer cells to PI3K inhibition we used RNAi screens in which protease-targeted murine breast cancer cells were subjected to two PI3K pathway inhibitors (Figure 2). Genetic screens have been repeatedly shown to be suitable for identifying synthetic lethal interactions, for instance by kinome-wide genetic interference (i.e. targeting all kinases; 54-57). The potential for proteases as synthetic lethal partners of PI3K inhibition has been neglected so far, although proteases are involved in all steps of cancer progression 26,58. Therefore, we report here the application of degradome-wide inducible miR-E-libraries in cancer settings in combination with PI3K inhibition.

In those synthetic lethality screens, we identified 181 proteases affecting the susceptibility of breast cancer cells to low dose PI3K pathway inhibitor treatment (Figure 3). The detection of many proteasome subunits as hits was seen as proof-of-principle validating general functionality of the system while highlighting the well-known importance of the ubiquitin-proteasome system (UPS) for general cell survival and proliferation (reviewed 44-46,59; Supplementary Figure S1). In line, proteasome inhibition by Bortezomib (Velcade) is known to have combinatory effects with PI3K inhibition by BEZ treatment in mantel cell lymphomas 60 and diffuse large B-cell lymphoma *in vitro* 61.

By applying additional selection criteria, 12 hits were identified and subjected to validation experiments (Figure 3). For all 12 hits, knockdown of the respective proteases by at least one of the two tested miR-E constructs sensitized murine PyB6-TA breast cancer cells to PI3K inhibition by BKM (Supplementary Figure S2). Finally, we decided to further investigate Usp7, Metap1 and Metap2 due to most distinct effects in the first validation assays and literature postulating tumor-promoting functions of these proteases 62-73. Metap1 and 2 (isoforms) are metalloproteases that co-translationally remove the initiator methionine from newly synthesized proteins 74,75. Metap2-inhibitors are known to act tumor suppressive in various *in vitro* and *in vivo* cancer settings including breast carcinoma 62-64. Metap2-inhibitors have been evaluated in clinical trials of solid cancers, but were discontinued due to several side effects such as neurotoxicity 76,77. In the present study we confirmed Metap2 as critical for breast cancer cells growth, in line with previous reports on its tumor-promoting function 62-64. In contrast to Metap2, Metap1-specific inhibition is relatively rarely investigated but was shown to have anti-tumor effects in cervical cancer and fibrosarcoma cells 65, as well as in human umbilical vein endothelial cells and human lung carcinoma cells 66. Here we discovered a dependency of breast cancer cells on Metap1. Specifically, knockdown of Metap1 or Metap2 impaired MTT viability and reduced competitive cell growth in murine breast cancer cells (Figure 4). Furthermore, reduced colony formation was observed in murine and human breast cancer cell lines (Figure 5). Interestingly, growth impairment was stronger for Metap2 knockdown, compared to Metap1, in line with this isoform being more extensively studied. The observed anti-growth phenotype upon Metap1 or 2 knockdown was linked to impaired cell cycle progression in human and murine cells (Figure 5); probably related to global reduction of protein synthesis mediated by increased inhibitory phosphorylation of eIF2α in PyB6-TA cells (Supplementary Figure S15). The heterotrimeric eIF2 complex is important for global protein synthesis and hence for cell division 78,79. It was previously shown that Metap2 interacts non-catalytically with eIF2α protecting it from inhibitory phosphorylation 51.

In addition, our data demonstrate a pivotal role of Usp7 for viability and competitive growth of breast cancer cells (Figures 4 and 6). In line with our data, the DUB Usp7, also known as Herpesvirus associated protease (HAUSP), is generally known to act tumor-promoting in various cancers 67-69,80, including breast cancer 70-73. Based on our results, we confirmed the tumor-promoting function of Usp7 in breast cancer. The Usp7 knockdown dependent growth reduction was linked to an impaired cell cycle progression that could be explained by significant mRNA upregulation of the cell cycle-inhibitor p21 in PyB6-TA and MCF7-TA cells (Figure 7). P21 is known to trigger cell cycle arrest in G1/S and G2/M phase 81. Because p53 is the major inducer of p21 82, loss of p53 could explain why p21 was not upregulated upon USP7 knockdown in p53 mutant MDAMB-TA cells 83. Indeed, Usp7 is best studied for its paradoxical role in regulating the turnover of the tumor suppressor p53 thereby balancing cell survival and death 69,84-87. Upon cell stress, Usp7-dependent deubiquitination facilitates p53 stability. In contrast, under stress-free conditions Usp7 stabilizes the E3-ubiquitin ligase Mdm2 leading to p53 ubiquitination and degradation by the proteasome 69,84-87. The growth reduction of human MDAMB-TA cells upon USP7 knockdown seems to be independent of p21. In this regard, Usp7 inhibition or depletion has been shown to impair cell cycle progression and proliferation also in a p53- independent manner by regulating genome stability and repair 69,88,89. Accordingly, pharmacological inhibition or knockdown of USP7 in human breast cancer cells with different p53 status is known to reduce cell proliferation, cell migration and colony formation 73.

Importantly, we discovered an advantage for combining Metap1, Metap2 or Usp7 knockdown with PI3K inhibition to target breast cancer cells. Combinatory knockdown of any of these proteases with PI3K inhibition decreased BKM EC20 and EC50 in two murine breast cancer cell lines (Figure 4). These cells were generated from the MMTV-PyMT mouse model harboring an activated PI3K signaling pathway 10,36-38. Interestingly, combined targeting had only small effects on the sensitivity of non-cancerous NMuMG/E9-TA cells. This points towards a specific effect in cancer cells with oncogenic activation of the PI3K pathway. This would be of particular interest because cancer-specific vulnerabilities make for excellent therapeutic options to specifically kill cancer cells 30-32.

Combinatory effects of protease targeting and PI3K inhibition on murine and human breast cancer cell growth was further validated in competitive growth assays and colony formation assays (Figures 4-6). Accordingly, combined protease knockdown and PI3K inhibition by BKM at EC10 or EC20 resulted in a synergistic reduction of colony growth in murine and human breast cancer cells. Those synergistic effects indicate synthetic lethal interactions of proteases and the PI3K pathway because the combinatory impairment led to a significantly stronger growth reduction than knockdown or PI3K inhibition alone.

The combinatory anti-growth effects of METAP1, METAP2 or USP7 knockdown and PI3K inhibition were also evident in other human cancer cells, namely two CRC (LoVo-TA and Caco2-TA) as well as two HCC (HuH7-TA; HEP-3B-TA) cell lines (Supplementary Figures S9-S11and S21). Indeed, there is growing evidence for oncogenic PI3K signaling in CRC and HCC 90-92. Mutations in the p110α coding gene PIK3CA occur in 15% to 18% of CRC cases 93,94 and deregulated mTOR signaling has been identified in 50-60% of HCC cases 90,95 due to frequent mutations in tuberous sclerosis complex-1 and 2 (TSC1/TSC2¸ 96). Although PI3K pathway inhibitors are upcoming in CRC and HCC treatment, monotherapy is not sufficient 92,97. This leads to the investigation of combinatory treatment options. For example, the dual PI3K/mTOR inhibitor PKI-587 was shown to radiosensitize HCC cells *in vivo* 98 and combined BKM and panitumumab (human EGFR antibody) or MEK Inhibitor Binimetinib treatments showed promising results in CRC patients 99,100.

Interestingly, the anti-growth effect of combined protease knockdown and PI3K inhibition were more prominent in breast cancer cells compared to HCC and CRC (Supplementary Figures S9-S11and S21). In competitive growth settings combinatory effects were hardly visible in CRC and HCC cells and in colony formation assays human breast cancer cells showed stronger total effects than HCC and CRC cells. Hence, we hypothesize that combined targeting of specific proteases and the PI3K pathway has a general combinatory effect on cancer cells, whereby effect strength seems to be cancer type dependent.

The molecular mechanism(s) underlying the combinatory anti-growth effect of simultaneous PI3K inhibition and protease targeting are difficult to uncover due to the manifold targets of Usp7 and Metap2 and the limited knowledge on Metap1. The anti-growth effects of Metap2 inhibition were previously linked to many substrates including hypophosphorylation (inactivation) of Retinoblastoma tumor suppressor protein (Rb) 66,101, delayed degradation of cyclin B and reduced cyclin A levels 65, decreased Src phosphorylation 102 and reduced anti-apoptotic Bcl-2 mRNA and protein expression 103. Due to its broad substrate specificity Usp7 is a global regulator of post-translational protein stability and localization of many substrates including Notch1 104, Rb 105, N-myc 106, c-myc 107, FoxP 108, β-catenin 109 as well as the tumor suppressor FOXO4 110.

In our experimental attempts we detected no combinatory treatment dependent changes in the mRNA expression of cyclin D1, the apoptosis mediator Puma or the cell cycle inhibitors p27 and p57 (Supplementary Figure S12-S13). In addition, we excluded involvement of several MAPK pathway related kinases in the combinatory effect of METAP1 or METAP2 knockdown with BKM treatment in human breast cancer cells (Supplementary Figure S14). In p53 wildtype PyB6-TA and MCF7-TA cells we found p21 upregulation upon Usp7 knockdown (Figure 7), which corresponds to the observed cell cycle impairment. However, also the p53 mutant cell lines Caco2-TA, HuH7-TA and MDAMB-TA showed reduced growth upon combinatory targeting of USP7/PI3K (Figure 6; Supplementary Figures S9 and S21). Thus, p53 independent functions of Usp7 must have a role. For knockdown of Metaps, we detected increased phosphorylation of eIF2α, which inhibits protein biosynthesis (Supplementary Figure S15). Based on those data, we suggest that protease depletion sensitized cells to PI3K inhibition, because protease knockdown already impaired cell viability and function. Combined PI3K inhibition would act like a second hit onto the cells already suffering from the protease knockdown, leading to combinatory effects. In addition, our data indicate that Metap1, Metap2 and Usp7 directly or indirectly interfere with the PI3K/AKT/mTOR signaling axis, thereby sensitizing further to PI3K inhibition (Supplementary Figure S16). Especially the significantly reduced phosphorylation of mTOR upon METAP1 knockdown in MDAMB-TA and METAP2 knockdown in MCF-TA cells was interesting since this disturbance is in line with reduced pS6 levels (Supplementary Figure S16). The changed PI3K pathway activity upon protease knockdown together with our RNA-Seq and RT-PCR data (Figure 1 and Supplementary Figure S3) indicate a possible connection of proteases to the PI3K pathway that was neglected so far. At least for Usp7, some direct links to the PI3K pathway are known. In breast cancer, Usp7 was shown to stabilize insulin receptor substrate 1/2, important for insulin/IGF-mediated PI3K signaling 72, and estrogen receptor α (ERα) 111. Furthermore, Usp7 can deubiquitinate PTEN to exclude it from the nucleus 28, facilitating PI3K-mTOR signaling in leukemia cells 112. In melanoma cells Usp7 inhibition was shown to increased expression of protein phosphatase 2 subunitBR3 isoform (PPP2R3A), which is known to dephosphorylate Akt 113 and in lung cancer cells Usp7 inhibition led to decreased levels of phosphorylated S6 (Ser235/236) as well as upregulation of two mTOR inhibitor proteins (FKBP11 and RGS16; 114). Metaps have to our knowledge not been closely linked to the PI3K pathway. Only one study in liver cancer cells that overexpress constitutively active Akt1/PKB-α showed a 1.5-fold higher METAP2 activity compared to wildtype cells indicating that the pathway somehow regulated Metap2 activity 115.

## Conclusions

In summary, based on data quality, internal controls and successful validation of the hits, we established a discovery pipeline to analyze the connection of proteases and PI3K signaling. This approach could be applied to other PI3K mutated cancers 5,9 or combined with other inhibitors 15, like Alpelisib 18, in the future. Our data confirmed the known tumor promoting function of Usp7 and Metap2 in murine and human breast cancer cells, postulating a new importance of Metap1 for breast cancer cell growth. Importantly, we discovered an advantage for combining PI3K inhibition with either Usp7, Metap1 or Metap2 knockdown. These combinations yielded partly synergistic effects on murine and human breast cancer cell growth, transferrable to human colorectal and hepatocellular carcinoma cell lines. To our knowledge such a combination was not suggested in breast cancer before and recent combinatory treatment focused on combining Usp7 inhibition with chemotherapeutics 116, PARP-inhibitors 117,118, Notch1 pathway inhibitors 104 or proteasome-inhibitors 119 in different cancer entities, but not in breast cancer. In line with our data, combination of USP7 and PI3K inhibition was suggested in leukemia cells 50 and more recently in TP53-mutant small-cell lung cancer cells 108. For Metap2 combined inhibition with chemotherapeutic or cytotoxic drugs was successfully tested in many different cancer entities 120-123, also excluding breast cancer. Combination with PI3K pathway inhibitors or focus on Metap1 was neglected so far. Based on our data we propose Usp7, Metap1 and Metap2 as synthetic lethal partners to simultaneous PI3K inhibition. Targeting of these proteases might enhance future combinatory breast cancer therapy by sensitizing breast cancer cells to inhibition of the PI3K pathway.

## Methods

### Plasmids

All used plasmids can be found in Supplementary Figure S22. For the generation of single target knockdown plasmids, 97 nucleotide oligonucleotides (97-mers) encoding the specific shRNAs in a miR-E backbone were ordered from Sigma-Aldrich or Thermo (List in Supplementary Table S6). 97-mers were PCR-amplified adding *XhoI* and *EcoRI* restriction sites by using the forward_5-mirE-Xhol (TACAATACTCGAGAAGGTATATTGCTGTTGACAGTGAGCG) and reverse_3-mirE-EcoRI (5´-TTAGATGAATTCTAGCCCCTTGAAGTCCGAGGCAGTAGGCA) primers. Resulting 116-mers were cloned into the pTREBAV vector double digested by *XhoI/EcoRI*. Sequences were verified by sequencing.

### Cell lines and cell culture

Information on cell line origin and establishment can be found in the Supplementary Methods. PyMG-816 and PyMG-TA stem cell like murine breast cancer cells were cultivated as previously described 124 under low oxygen atmosphere (hypoxia [3% O_2_, 5% CO_2_, 92% N_2_]) at 37°C. All other breast cancer cell lines were cultured in DMEM high glucose, pyruvate supplemented with 10% fetal bovine serum, 1% Penicillin/Streptomycin and 1% L-Glutamine at 37 °C with 5% CO_2_ and 21% O_2_. Colorectal cancer (CRC) Caco2-TA cells were cultured in MEM (with Earle´s salt) supplemented with 10% fetal bovine serum, 1% non-essential amino acids and 1% Penicillin/Streptomycin. LoVo-TA cells were cultured in Ham´s F12 medium supplemented with 10% fetal bovine serum, 1% Penicillin/Streptomycin and 1% L-Glutamine. Hepatocellular carcinoma (HCC; HEP-3B-TA, HuH7-TA) cell lines were cultured in MEM (with Earle´s salt; HEP-3B-TA) or RPMI (HuH7-TA) supplemented with 10% fetal bovine serum, 1% non-essential amino acids, 1% Penicillin/Streptomycin and 1% L-Glutamine.

For generation of miR-E-transduced cells the respective PyB6-TA, PyMG-TA, NMuMG/E9-TA, MCF7-TA, MDAMB-TA, Caco2-TA, LoVo-TA, HuH7-TA or HEP-3B-TA cells were transduced with undiluted ectopically packed single-target knockdown plasmids and were selected with Blasticidin (PyB6-TA/PyMG-TA: 10 µg/ml, MDAMB-TA/MCF7-TA: 26 µg/ml; NMuMG/E9-TA: 6 µg/ml; Caco2-TA/LoVo-TA/HuH7-TA/HEP-3B-TA: 20 µg/ml) whereby Puromycin selection was continued (Supplementary Methods) until cells were frozen. All cell lines were tested for Mycoplasma contaminations prior freezing and transduction in-house.

### The degradome-wide knockdown system

A customized degradome-focused enhanced microRNA (miR-E) library targeting 658 murine protease and protease-like transcripts with ≈ 4800 miR-Es (4-7 miR Es/target) was used. The miR-E library was custom ordered (CustomArray, Inc) and was designed based on the Degradome database 33,125 as of 2014 using the shERWOOD algorithm for scoring and ranking of miR-E potency 126. The library was PCR cloned into the pTREBAV plasmid and subdivided into 16 miR-E-pools (≈ 300 miR-Es/pool).

Degradome-targeted cells were generated via transduction of PyB6-TA cells with 1:6 diluted ectopically packed miR-E-library pool plasmid retrovirus supplemented with Polybrene [8 μg/ml]. PyB6-TA cells were cultured for 24 h in starvation medium (full DMEM 1% FCS) for cell cycle synchronization prior seeding. Infection of 9.75 x 10^6^ cells spread on 13 plates per miR-E-pool was done 16 h after seeding to infect shortly before M-phase. Transduction efficiency was controlled 48-72 h after infection by flow cytometry, following Blasticidin selection (10 µg/ml) until over 80% of fluorescent cells were obtained. Puromycin (4 µg/ml) and Neomycin (500 µg/ml) selection were repeated. Two independent transductions were performed per miR-E-pool using independently generated viral supernatant. Target cells were prepared to guarantee a 1000-fold miR-E representation and the 1:6 dilution was chosen to ensure single-miR-E-copy integration based on prior establishment experiments. Four commonly used miR-30a based shRNA controls were incorporated during ecotropic virus production as one construct per miR-E-pool to serve as internal quality controls. Stability controls: pTCEBAC-shRenilla 127; pTCEBAC-shLuciferase 128; Depletion controls: pTREBAV-shRpa3-218 (termed Rpa3.276 in 127); pTREBAV-shRpa3-457 (termed Rpa3.455 in 128; sequence information Supplementary Table S6).

### Pantropic virus production

Pantropic virus was produced by co-transfection of HEK-293T cells with lentiviral pLNN (5 µg), packaging pCMV (3 µg) and envelope pVSV-G (1.5 µg) plasmids in Opti-MEM using Superfect (301305; Qiagen). Medium was exchanged after 3 h (8 ml) and 48 h later the viral supernatant was harvested by filtration (0.45 µm), aliquoted and frozen at -80°C. Alternatively, Phoenix-GP or HEK-293T cells were co-transfected with retroviral pMSCV-rtTA3-IRES-EcoReceptor-PGK-Puro plasmid (8 µg), pSuper-shDGCR8 (2 µg; only for MCF7 and MDA-MB-231 cells) and envelope pVSV-G (2 µg) plasmids in Opti-MEM using Turbofect (R0532; Thermo Fischer), following incubation for 20 min at RT. The medium was exchanged after 14 h (Phoenix-GP cells) or 24 h (HEK-293T cells). Viral supernatant was harvested 12 h after medium exchange by filtration (0.45 µm). Packaging cells were covered again with 8 ml medium and harvesting was repeated two times (three times in total).

### Ecotropic virus production

Plat-E cells were transfected with 8 μg DNA (6.5 µg miR-E-library DNA, 21.38 ng of each control [shRpa3-218; shRpa3‑457; shRenilla; shLuciferase] and 1.5 µg pSuper-shDGCR8) in Opti-MEM using Polyethylenimine (PEI [3 µl/µg DNA]). For non-library plasmids (pMSCV-rtTA3-PGK-Puro or single-target knockdown plasmids) 4 µg plasmid DNA without pSuper-shDGCR8 or the four controls was used. Cells were incubated (6 h, 37°C) before medium was exchanged to 12 ml DMEM. After 72 h, the Plat-E supernatant was filtered (Ø 0.45 μm) and used fresh.

### Negative selection synthetic lethality screen

Degradome-targeted murine breast cancer cell pools were treated for 14 days as following: DMSO; DMSO + Dox; BKM; BKM + Dox; BEZ; BEZ + Dox using inhibitors at EC20 and DMSO as solvent control. At each passage 1.1 x 10^6^ cells/plate were kept to maintain miR‑E representation. One miR-E-pool was cultured per 15 cm plate. Genomic DNA was isolated after 14 days (2 x 10^6^ cells/plate) using the Gentra Puregene Cell Kit (158767; Qiagen). Deep sequencing template libraries were generated by PCR amplification of the miR-E cassettes using flanking primers that tag the product with standard Illumina P5/P7 adapters as well as a sample specific 10 nucleotide barcode sequence (BC; forward miR-E-P7-BCx-F: CAAGCAGAAGACGGCATACGAGATNNNNNNNNNNGTATATTGCTGTTGACAGTGAGCG; reverse PGK-P5-rev-No2: AATGATACGGCGACCACCGAGATCTACACTACCGGTGGATGTGGAA TGTGTGC). A different barcode primer was used for each sequencing sample whereby 4 screen samples were amplified per barcode. One 50 μl PCR reaction, containing 2 μg template, 1x AmpliTaq Gold buffer, 0.3 mM of each dNTP, 0.3 μM of each primer and 2.5 U AmpliTaq Gold (Applied Biosystems), was performed using the following cycling parameters: 95°C 10 min (1.2 °C/sec heating rate); 35 cycles 95°C (30 sec 1.5 °C/sec heating rate), 53°C 30 sec (1.4 °C/sec heating rate), 72°C 60 sec (1.6°C/sec heating rate); 72°C 10 min. PCR efficiency was analyzed by gel electrophoresis and barcode-tagged PCR products (315 bp) were combined according to their relative abundance in gels to archive equal sequencing reads generating one sample for sequencing. The number of pooled samples per sequencing run was calculated using a theoretical sequencing depth of 1 x 10^6^ bp per sample. The sequencing sample was further column purified (QIAquick PCR purification kit [28104; Qiagen]), the concentration was adjusted to 0.832 nM for optimal cluster generation and libraries were analyzed on an Illumina NextSeq500 in the Department for Pediatric Genetics at the Medical Center Freiburg. 75bp single-end sequencing was utilized to read out the miR-E cassette using the Illumina sequencing primer (5´- TAGCCCCTTGAAGTCCGAGGCAGTAGGCA) and a custom index primer (5'-CGCTCACTGTCAACAGCAATATAC) to sequence the 10 bp barcode as index reads.

### Quantitative analysis of screen hits and hit selection

Sequence processing was performed generating the raw number of reads for each miR-E in the library per sample. The raw number of reads was further cleaned, pool-based normalized, Log2 transformed and trimmed. For effect calculation the robust strictly standardized median difference (AvSSMD*) calculated after a modified version of the method-of-moment estimate of the paired SSMD* by Zhang X.D 40 was chosen being suitable for screens with biological duplicates only. Detailed information on sequence processing and effect calculation can be found in the Supplementary Methods. Hits were defined as proteases with minimum two miR-Es per protease scoring outside of the ± 1 standard deviation from the AvSSMD*s of all constructs in the screen (SD_AvSSMD*). Hits were further filtered for miR-E target mRNA expression (FPKM ≥ 0.5; previously published data on PyB6-313 cells 129).

### Cell treatment

PI3K-inhibitor and Usp7-inhibitor treatments were performed as indicated in Table 1 and Table 2 after letting cells attach overnight. EC values were determined by 48 h MTT assays (Supplementary Figures S17-S18 and S23). DMSO was used as solvent control at the same dilution as the respective inhibitor. Treatment was renewed every 2-3 days ensuring renewal the day prior experiments. Dox was used at 2 µg/ml. BKM (NVP-BKM-120 [Buparlisib], CT-BKM120; Chemitek), BEZ (NVP-BKM-235 [Dactolisib], S1009; selleckchem.com) GNE6776 (HY-107986; MedChem express) and P005091 (HY-15667; MedChem express) were dissolved in DMSO.

### MTT assay

Cells were cultured ± Dox for three days prior seeding onto 96 well plates (0.05 x 10^5^ cells/well [PyB6-TA; NMuMG/E9-TA]; 0.08 x 10^5^ cells/well [PyMG-TA]) in triplicates. PI3K-inhibitor treatment was performed with the following concentration series maintaining Dox treatment. BKM: 200000, 100000, 20000, 10000, 2500, 500, 0 nM; (+ 100 nM in NMuMG/E9-TA); DMSOBKM: 1:250 dilution; BEZ: 10000, 1000, 500, 250, 50, 10, 0 nM (+ 5; 1 nM in NMuMG/E9-TA); DMSOBEZ: 1:1000 dilution. To determine effective concentration (EC) values cells were seeded without Dox pre-treatment onto 96 well plates (0.05 x 10^5^ cells/well [PyB6-TA, MCF7-TA, MDAMB-TA]; 0.1 x 10^5^ cells/well [Caco2-TA, LoVo-TA, HuH7-TA, HEP-3B-TA]) and inhibitor treatment was performed with the concentration series in Table 3. DMSO was used at the same dilution of the highest inhibitor concentration.

After 48 h of treatment cells were incubated for 1-6 h with indicator-free medium containing MTT (0.5 mg/ml). Resulting formazan crystals were dissolved in DMSO and the absorbance was measured at 570 nm (650 nm reference) using an EnSpire multimode plate reader. 570 nm absorbance was normalized to reference readings at 650 nm and MTT viability was calculated relative to untreated (no PI3K-inhibitor, no Dox) cells averaged per triplicate. The mean MTT viability of all biological replicates plotted against Log10 concentrations was used to calculate dose-response curves and corresponding effective concentrations (EC) with standard error (SE) in OriginPro (non-linear fit, category: growth/sigmoidal, function: DoseResp).

### Competitive growth flow cytometry assay

MiR-E-transduced PyB6-TA cells were mixed with non-fluorescent PyB6-TA cells (≈ 30% to 70%) and were treated for 8 days with BKM or BEZ (EC10 or EC20) ± Dox. MiR-E-transduced Caco2-TA, LoVo-TA, HuH7-TA and HEP-3B-TA cells were mixed with miR-E-Renilla-transduced cells (≈ 30% to 70%) and were treated for 8 days with BKM or BEZ (EC10, EC20, EC50) ± Dox. DMSO was used as solvent control. Flow cytometry was performed at day 0 and day 8 using the Cytoflex SFlow cytometer (Beckmann Coulter). For calculation the day 8 samples were used. For each biological replicate the percentage of dsRed+ cells in PI3K‑inhibitor-Dox or DMSO-Dox treated samples was set relative to Venus+ cells in the respective not Dox treated sample, whereby 100 was subtracted generating the relative depletion of fluorescent cells e.g. Depletion [%] = (dsRed_DMSO+DOX_ * 100 / Venus_DMSO_) - 100, followed by calculation of the relative mean depletion for all biological replicates.

### Plate colony formation assay

Cells were separated (70-100 µM cell strainer) and seeded at single-cell conditions onto 6 well plates or 12 well plates. After 24 h, PI3K‑inhibitor (EC10, EC20, EC50) and/or Usp7-inhibitor (P005091; EC10, EC20, EC50) treatment ± Dox was performed. DMSO was used as solvent control. After 7-10 days (PyB6-TA) or 10-12 days (MCF7‑TA/MDAMB‑TA/Caco2-TA/LoVo-TA/HuH7-TA/HEP-3B-TA) cells were stained with 1% crystal violet in 20% methanol (10 min) and photographed in raw format using a light desk and the canon powershot G6 camera. Raw files were converted to 800 dpi tiff files using Adobe Photoshop CS2. The ImageJ plugin Colony Area by Guzmán et al. was used due to the provided manual 130 analyzing colony intensity percent, which was termed cell/colony growth. Reduction in growth was calculated relative to DMSO-only treated cells for each biological replicate e.g. growth reduction BEZ [%] = (Intensity percent BEZ * 100 / Intensity percent DMSO) - 100. The mean growth reduction relative to DMSO was calculated for all biological replicates. The difference in mean growth reduction relative to DMSO between PI3K‑inhibitor-Dox treated and PI3K-inhibitor-only treated cells was calculated by subtraction. For the DMSO-only and DMSO-Dox treated conditions data from the EC10 and EC20 plates were pooled.

### Hoechst cell cycle assay

Cells were cultured for three days ± Dox and seeded onto 24 well plates (0.3 x 10^6^ cells/well). After 5-6 h, medium starvation (full DMEM 0% FCS) with maintained Dox treatment was applied for 24 h following PI3K-inhibitor treatment at EC20 ± Dox for 12 h (PyB6-TA) or 24 h (MDAMB-TA). DMSO was used as solvent control. Cells were stained with 5 µg/ml Hoechst 33342 in DMEM at 37°C for 30 min following flow cytometry analysis using the LSR II. A starved, untreated control was used to set the gates for G1-phase. G2-phase gates were set as double fluorescence intensity of G1. For Dox treated samples Venus+/dsRed+/Hoechst+ cells and for not Dox treated samples Venus+/Hoechst+ cells were used. Difference in Hoechst+ cells per cell cycle gate was calculated relative to DMSO-only treated cells for each biological replicate e.g. Hoechst+ G1 BEZ [%] = (Hoechst+ G1_dsRed_BEZ * 100 / Hoechst+ G1_Venus_DMSO) - 100. Following, the mean was calculated for all biological replicates. The difference in mean Hoechst+ cells per cell cycle phase relative to DMSO was calculated between PI3K-inhibitor-Dox treated and PI3K‑inhibitor-only treated by sample subtraction.

### Human MAPK phosphorylation antibody array

The human MAPK phosphorylation antibody array (ab211061; Abcam) was used due to manufacturer´s instructions. MiR-E-METAP1- or miR-E-METAP2-transduced MDAMB-TA or MCF7-TA cells were treated for 4 days with BKM at EC20 ± Dox. Chemiluminescent signal was detected using the Fusion SL Detection System (Vilber Lourmat) and FusionCapt Advance software (Vilber Lourmat). For analysis the ImageJ plugin Protein Array Analyser by Carpentier and Henault was used 131. The average integrated area from 4 random background spots was subtracted from each antibody spot and then the signal was normalized to the average integrated area of the reference spots.

### Flow cytometry

Cells were resuspended in culture medium or FACS buffer (DPBS, 2% FCS, 5 mM EDTA [competitive growth assay]). Analysis was performed using the LSR II (BD) or the Cytoflex SFlow cytometer (Beckmann Coulter) and the BD FACS Diva 6.1.2 software. Viable cells were gated (forward-scattered area [FSC‑A] versus side-scattered area [SSC-A]) and further restricted to singlets (forward-scattered width [FSC-W] versus height [FSC-H]). Living, single cells were further restricted by individual gates for the fluorescent cells depended on the experiment. All biological replicates were gated using the same defined gates.

### Real-time PCR (RT-PCR)

Total RNA was isolated from cells using the peqGOLD Total RNA Kit (12-6936-01; Peqlab) incorporating a DNA digestion step using the RNase-Free DNase Set (79254; Qiagen). Conversion of 1 µg RNA into complementary DNA (cDNA) was done using the iScript™ cDNA Synthesis Kit (1708891; Bio-Rad). Real-time PCR was carried out in triplicates using the SYBR Green PCR Master Mix (Applied Biosystems) and the CFX384 Touch (384 well plate) Real-Time PCR Detection System (Bio-Rad). Beta-actin served as endogenous normalization control. RT-PCR primers are stated in Supplementary Table S7. For data analysis the BioRad CFX Manager 3.1 was used. The mean Cq of the triplicates was normalization to ß-Actin (ΔCT = CT_Gene_-CT_ßActin_). For relative normalized mRNA expression 2^-ΔCT^ was used.

### Protein Isolation and Immunoblotting (Western Blot)

Cells were harvested on ice by scraping in Phospho-RIPA lysis buffer (Tris-HCL [50 mM, pH 7.5], NaCl [150 mM], Triton X100 [1%], Sodiumdeoxycholate [0.5%], SDS [0.1%], EDTA [1 mM, pH 7], Natriumpyrophosphate [2.5 mM], β-Glycerophosphate [1 mM], Natriumvanadat [1 mM], PhosStop Phosphatase-inhibitor mix [04906845001; Roche], Complete Ultra tablets [5892970001; Sigma-Aldrich] in ddH_2_0) followed by mechanical disruption. Cell lysates containing 25-50 μg protein were subjected to SDS-PAGE and were transferred to a PVDF (GE Healthcare) or Nitrocellulose membrane (Amersham life Science) using a wet blot system (BioRad). PVDF membranes were blocked with 3% BSA in TBS-Tween (0.1%, 1 h). Nitrocellulose membranes were dried overnight and blocked with 3% BSA in TBS (0.1%, 1 h). Following blocking, membranes were incubated with primary antibodies (Table 4) overnight at RT or 4°C. Membranes were washed with TBS-T and incubated with the corresponding goat-anti-mouse-horseradish-peroxidase (A0168; Sigma [1:12000]), respectively goat-anti-rabbit-horseradish-peroxidase (111-035-003; Jackson laboratories [1:12000]), or fluorescence coupled IRDye® 800CW Donkey anti-Mouse IgG (926-32212; LI-COR [1:12000]) or IRDye® 680RD Donkey anti-Rabbit IgG (926-68073; LI-COR [1:12000]) secondary antibodies for 60-120 min at RT, washed again, and developed. Chemiluminescent signals were detected after addition of West Pico/Femto Chemiluminescent Substrate (34080/34095; Thermo Scientific) using a Fusion SL Detection System and FusionCapt Advance software (Vilber Lourmat). For detection of peIF2α and total eIF2α first the phosphorylated protein was detected, the signal was stripped in stripping buffer (10 min, 37°; 21059; Thermo Scientific), following washing steps (15 min TBS; 2 x 15 min TBS-T), blocking and antibody incubation. Fluorescent signals were detected and analyzed using the LI-COR ODYSSEY CLx Imager. Protein quantification of chemiluminescent signals (volume under the signal peak) was done relative to α-tubulin (probed on the same membrane) using the FusionCapt Advance software applying the automatically set rolling ball function for background correction. Fluorescent signals were quantified using Image studio 5.2 (LI-COR). Phosphorylated and reference unphosphorylated signals were first normalized to Tubulin (if plotted on different membranes) and were further normalized between treated and untreated conditions (probed on the same membrane) before the ratio between phosphorylated and unphosphorylated signals was calculated.

### AKT (Total/Phospho) ELISA kit

The AKT (Total/Phospho [Ser473]) Multispecies InstantOne™ ELISA Kit (85-86046-11; Thermo Fischer) was used due to manufacturer's instructions. MCF7-TA and MDAMB-TA cells transduced with the most potent miR-E against METAP1, METAP2 and USP7 were seeded (50.000 cells/well) onto 12 well plates following knockdown induction on half of the wells for 4 days. Cell lysates were used fresh.

### RNA-Sequencing (RNA-Seq)

MCF7, MDA-MB-231 and PyB6‑313 cells were seeded, 16 h later RNA was isolated (T0_Medium) and remaining plates were treated with BKM at EC50 (200 nM [MCF7], 1300 nM [MDA-MB-231], 1500 nM [PyB6-313]), or the same dilution of DMSO. After 3 h of treatment, RNA for the T3 condition was isolated. Conditions were prepared in triplicates. RNA was isolated using the Universal RNA Purification Kit (E3598; Roboklon) incorporating a DNA digestion step using the RNase-Free DNase Set (79254; Qiagen). RNA quantity, integrity, and quality was measured using a Bioanalyser. RNA was diluted in RNAse-free H_2_0 to obtain 40 µl of 40 ng/ml RNA, was prepared according to the DKFZ manual and sequenced with 31-47 Mio reads using the Illumina HISEQ4000 (W190 high Throughput Sequencing Unit; core facility genomics and proteomics; DKFZ Heidelberg). Raw data was trimmed using Trimmomatic 132 and alignment was performed with Kallisto 133. Log fold changes (logFC) were determined using least square fit with Benjamini-Hochberg correction for the following comparisons: DMSO versus BKM; DMSO versus T0_Medium; DMSO_ BKM versus T0_Medium. Differentially expressed genes (adj. p-value ≤ 0.05) in the DMSO versus BKM comparison were filtered for degradome-encoded transcripts only (Merops database 33 as reference for human cell lines; miR‑E library for murine) and average expression ≥ 1. Data was further divided into logFC > 0 (upregulated) and logFC < 0 (downregulated). The R package “eulerr” was utilized to generate Venn diagrams 134.

### General statistical analysis and data presentation

Statistical analyses were carried out with OriginPro 2018/2020 (OriginLab). In general, quantitative data of independent biological replicates (n) were plotted as mean ± standard deviation (SD), if not stated differently. Experiments performed with technical replicates were corrected for SD ≥ 0.1. For calculation of means of biological replicates values outside mean ± 2 SD were excluded. Statistical significance of means was determined by one-sample or two-sided two-sample t-test (p ≤ 0.05 significance level). Graphical depiction was done using OriginPro 2018/2020 (OriginLab). The graphical abstract was created with BioRender.com.

### String analysis

Search Tool for the Retrieval of Interacting Genes/Proteins (STRING; 135) analysis was performed on the synthetic lethality screen hits of the first selection criteria (≥ 2 miR-Es outside ± 1 SD_AvSSMD*). Analysis parameters were used as default with allowing textmining, experiments and databases as active interaction sources only. Confidence was chosen as meaning of network edges. STRING-based networks were further processed in Cytoscape 3.8.2 136 to increase label size and change node colors, whereby un-connected nodes were removed.

## Figures and Tables

**Table 1:** Concentrations of PI3K-inhibitors and Usp7-inhibitors for breast cancer cells

Compound		Concentration		
		PyB6-TA	MCF7-TA	MDAMB-TA
**BKM** (stock 50 mM)	EC10	0.5 µM	0.03 µM	0.34 µM
	EC20	1 µM	0.05 µM	0.67 µM
	EC50	2.1 µM	0.32 µM	1.5 µM
**BEZ** (stock 10 mM)	EC10	20.5 nM	0.85 nM	0.6 nM
	EC20	41 nM	1.7 nM	1.2 nM
	EC50	99.4 nM	42 nM	13.5 nM
**P005091** (stock 100 mM)	EC10	2.5 µM	3.65 µM	3.7 µM
	EC20	5 µM	7.3 µM	7.4 µM
	EC50	7.6 µM	13.3 µM	9.6 µM

PyB6-TA/MCF7‑TA/MDAMB-TA: miR-E-transduced cell lines.

**Table 2:** Concentrations of PI3K-inhibitors for colorectal and hepatocellular cancer cell lines

Compound		Concentration			
		Caco2-TA	LoVo-TA	HuH7-TA	HEP-3B-TA
**BKM** (stock 150 mM)	EC10	0.2µM	0.1 µM	0.4 µM	0.3 µM
	EC20	0.3 µM	0.3 µM	0.7 µM	0.5 µM
	EC50	1.5 µM	1.2 µM	1.1 µM	1.7 µM
**BEZ** (stock 10 mM)	EC10	1.5 nM	0.2 nM	0.4 nM	23.4 nM
	EC20	2.9 nM	0.3 nM	0.7 nM	46.7 nM
	EC50	65.8 nM	39.4 nM	36.0 nM	409.9 nM

Caco2-TA/LoVo‑TA/HuH7-TA/HEP-3B-TA: miR-E-transduced cell lines.

**Table 3:** Concentrations of inhibitors for EC determination

Cell line	Inhibitor	Concentration [nM]
PyB6-TA*	GNE6776	800000; 400000; 200000; 150000; 100000; 50000; 10000; 5000; 1000; 100; 10; 1; 0
	P005091	300000; 100000; 50000; 25000; 10000; 8000; 4000; 1000; 100; 10; 1; 0.1; 0
MCF7-TA*	GNE6776	1000000; 800000; 400000; 200000; 150000; 100000; 50000; 10000; 5000; 1000; 100; 10; 1; 0
	P005091	500000; 300000; 200000; 100000; 50000; 25000; 20000; 15000; 10000; 8000; 4000; 1000; 100; 10; 1; 0.1; 0
MDAMB-TA*	GNE6776	800000; 400000; 200000; 150000; 100000; 50000; 10000; 5000; 1000; 100; 10; 1; 0
	P005091	500000; 300000; 100000; 50000; 25000; 10000; 8000; 4000; 1000; 100; 10; 1; 0.1; 0
**Caco2-TA**	BKM	500000; 200000; 100000; 50000; 20000; 10000; 5000; 2500; 1000; 500; 100; 10; 1; 0.1; 0.01; 0
	BEZ	50000; 30000; 20000; 10000; 5000; 2500; 1000; 100; 10; 1; 0.1; 0.01; 0
**LoVo-TA**	BKM	500000; 200000; 100000; 50000; 10000; 5000; 2500; 1000; 500; 100; 10; 1; 0.1; 0
	BEZ	20000; 10000; 5000; 2500; 1000; 100; 10; 1; 0.1; 0.01; 0
**HuH7-TA**	BKM	500000; 200000; 100000; 50000; 20000; 10000; 5000; 2500; 1000; 500; 100; 10; 1; 0.1; 0.01; 0
	BEZ	50000; 30000; 10000; 5000; 2500; 1000; 100; 10; 1; 0.1; 0.01; 0
**HEP-3B-TA**	BKM	500000; 200000; 100000; 50000; 20000; 10000; 5000; 2500; 1000; 100; 10; 1; 0.1; 0.01; 0
	BEZ	50000; 30000; 10000; 5000; 2500; 1000; 100; 10; 1; 0.1; 0.01; 0

*PyB6-TA/ MCF7-TA/MDAMB-TA/NMUMG-TA BEZ and BKM concentrations as described in the MTT assay description

**Table 4:** Primary antibodies for western blot

Cell line	Dilution	Order number; Company
peIF2α (Ser51); rabbit	1:500	3398S; Cell signaling
eIF2α; mouse	1:500	2103S; Cell signaling
Metap1; rabbit	1:1000	A305584A-M; Thermo fisher
Metap2; rabbit	1:1000	12547; Cell signaling
S6 ribosomal protein; mouse	1:1000	2317S; Cell signaling
pS6 Ribosomal Protein (Ser235/236); rabbit	1:1000	4857S, Cell signaling
mTOR; rabbit	1:250	2983; Cell signaling
pmTOR (Ser2448); rabbit	1:500	5536; Cell signaling
Tubulin-α; mouse	1:1000	T9026; Sigma
Usp7; rabbit	1:660	ab101648; Abcam

## Abbreviations

ATXN3: Ataxin-3
AvSSMD*: Robust strictly standardized median difference
BEZ: NVP‑BEZ‑235 (Dactosilib)
BKM: NVP-BKM-120 (Buparlisib)
cDNA: Complementary DNA
CRC: Colorectal carcinoma
Ctsa: Cathepsin A
DESI1: Desumoylating isopeptidase 1
Dox: Doxycycline
DUB: Deubiquitinase
EC: Effective concentration
eIF2α: Eukaryotic initiation factor 2 subunit alpha
FPKM: Fragments per kilo base per million mapped reads
FSC‑A: Forward-scattered area
FSC-W/-H: Forward-scattered width/ height
HAUSP: Herpesvirus associated protease (aka. Usp7)
incl.: including
HCC: Hepatocellular carcinoma
LGMN: Legumain
LogFC: Log fold change
MAPK: Mitogen activated protein kinase
Metap1/Metap2: Methionine aminopeptidases 1 and 2
MBTPS1: Membrane-bound transcription factor site-1 protease
Mdm2: Mouse Double Minute 2
miR-E: enhanced microRNA
Mmp17: Matrix metallopeptidase 17
MMTV-PyMT: mouse mammary tumor virus Polyoma middle T
Otud5: OTU domain-containing protein 5
pAkt: phosporylated Akt
PEI: Polyethylenimine
PI3K/mTOR: Phosphatidylinositol-3-kinase/mammalian Target of Rapamycin
Pmpcb: Mitochondrial-processing peptidase subunit beta
pmTOR: phosporylated mTOR
pS6: phosporylated S6
Psmd13: 26S proteasome non-ATPase regulatory subunit 13
pTCEBAC: TRE-Cyan-miR-E-PGK-BSDr-2A-Cherry
PTEN: Phosphatase and Tensin homolog
pTREBAV: TRE-dsRed-miR-E-PGK-BSDr-2A-Venus
Rb: Retinoblastoma tumor suppressor
RNAi: RNA interference
Rpa3: Replication protein A3
RT: Room temperature
RT-PCR: Real-time PCR
rtTA3: reverse Tetracycline transactivator 3
SENP1: Sentrin-specific protease 1
Serine: Ser
Stambpl1: STAM Binding Protein Like 1
SE: Standard error
SD: Standard deviation
shRNA: small haiprin RNA
SPPL3: Signal peptide peptidase-like 3
SSC-A: Side-scattered area
Tnip1: TNFAIP3-interacting protein 1
TNFAIP3: Tumor necrosis factor alpha-induced protein 3
TRE: Tetracycline responsible element
Usp7: Ubiquitin carboxyl-terminal hydrolase 7
Usp30/33/38/9x: Ubiquitin carboxyl-terminal hydrolase 30/33/38/9x
Uspl1: SUMO-specific isopeptidase Uspl1
Vcpip1: Deubiquitinating protein Vcpip1
