# Supplementary material for: RNA interference screens discover proteases as synthetic lethal partners of PI3K inhibition in breast cancer cells
Source: Theranostics. 2022 May 16;12(9):4348–73. doi: 10.7150/thno.68299 (PMC9169373; doi:10.7150/thno.68299)
Supplement: Supplementary file 1 — Supplementary figures. [file thnov12p4348s1.pdf]

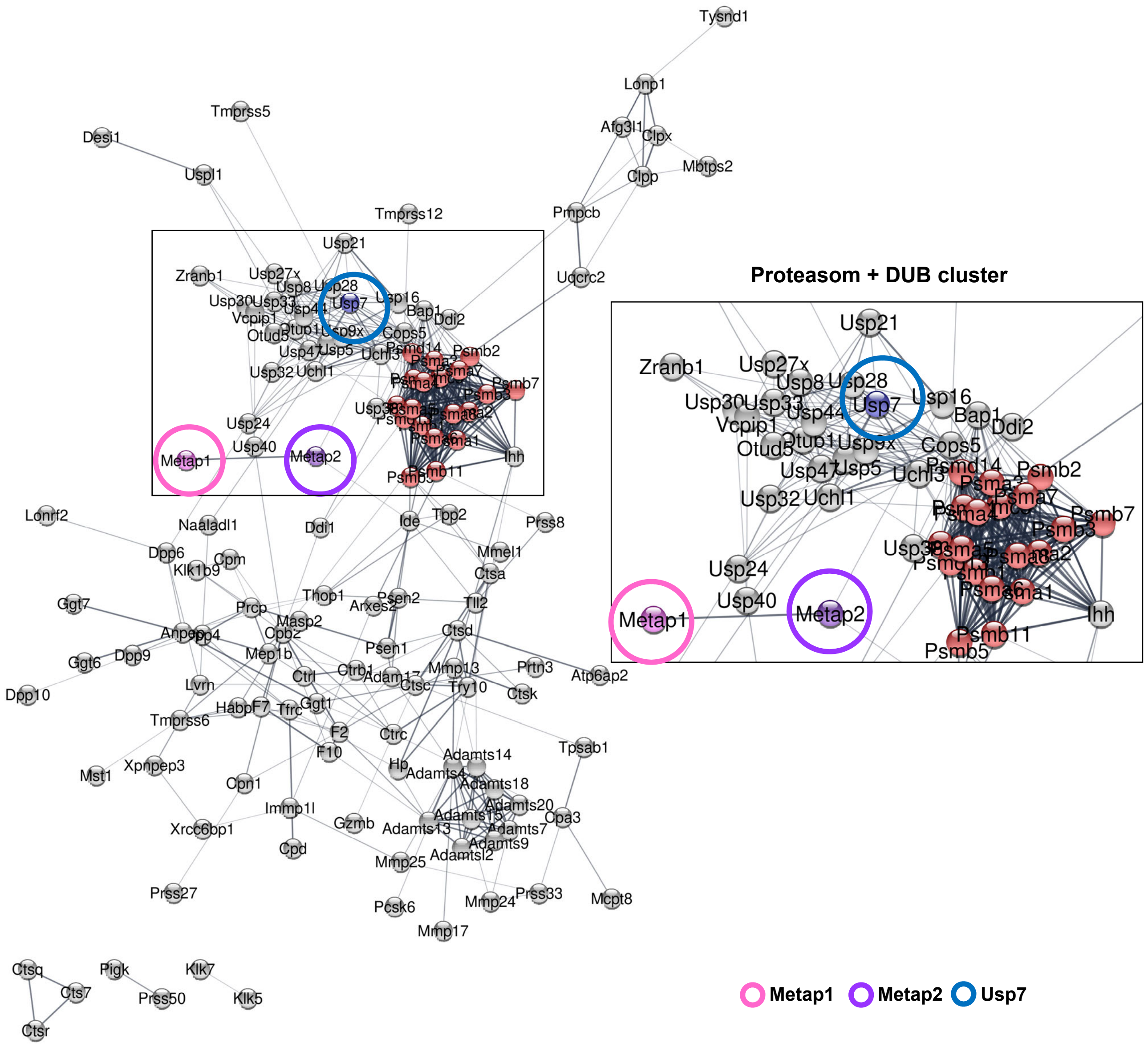

Figure S1: STRING-based cluster analysis of 181 hits from the synthetic lethality screens. Hits selected based on criteria 1A (≥ 2 miR-Es outside ± 1 SD_AvSSMD*; Figure 3E) pooled for the BKM and BEZ screens. Proteasome and Deubiquitinase (DUB) cluster magnified. Red: Proteasomal subunits Psma1-8, Psmb1-7, Psmb11, Psmc5, Psmd13, Psmd14. Proteases chosen for detailed validation in colored circles.

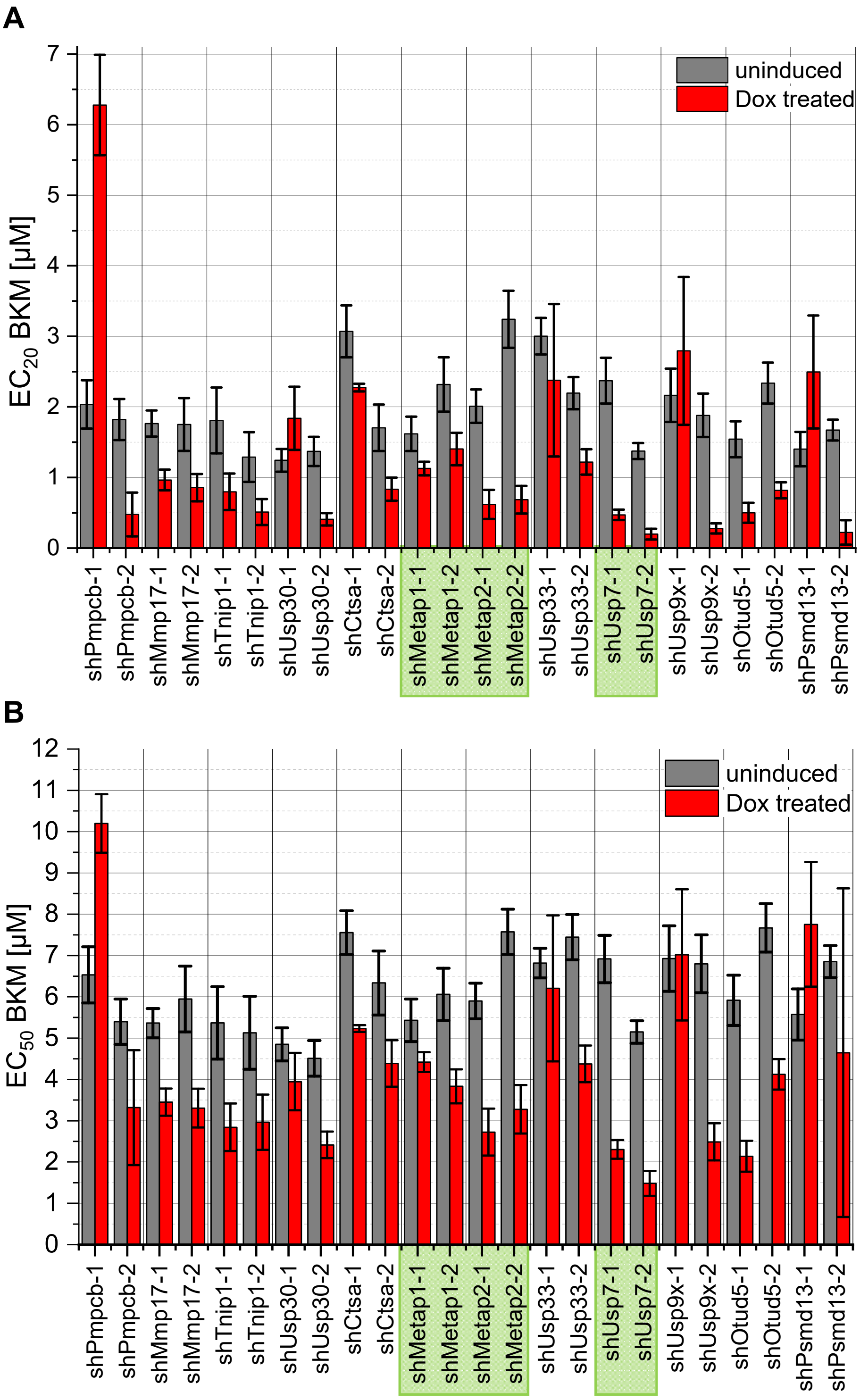

Figure S2: *In vitro* validation assays. BKM EC20 (**A**) and EC50 (**B**) values ± SE of miR-E-induced (Dox treated) and uninduced PyB6-TA cells calculated from MTT viability assay dose-response curves. MTT assay with 48 h BKM (concentration series) ± Dox. MTT viability as mean (n ≥ 3) relative to untreated, uninduced cells used for the dose response curves. Green: chosen hits based on literature research and effect strength. Fold change of Dox treated cells relative to uninduced cells for the 3 hits see Figure 4A/B. For simplification miR-Es are indicated by sh. Ctsa: Cathepsin A; Metap1/2: Methionine-aminopeptidases 1 and 2; Mmp17: Matrix metallopeptidase 17; Otud5: OTU domain-containing protein 5; Pmpcb: Mitochondrial-processing peptidase subunit beta; Psmd13: 26S proteasome non-ATPase regulatory subunit 13; Tnip1: TNFAIP3-interacting protein 1; Usp7: Ubiquitin carboxyl-terminal hydrolase 7; Usp30/33/9x: Ubiquitin carboxyl-terminal hydrolase 30/33/9x.

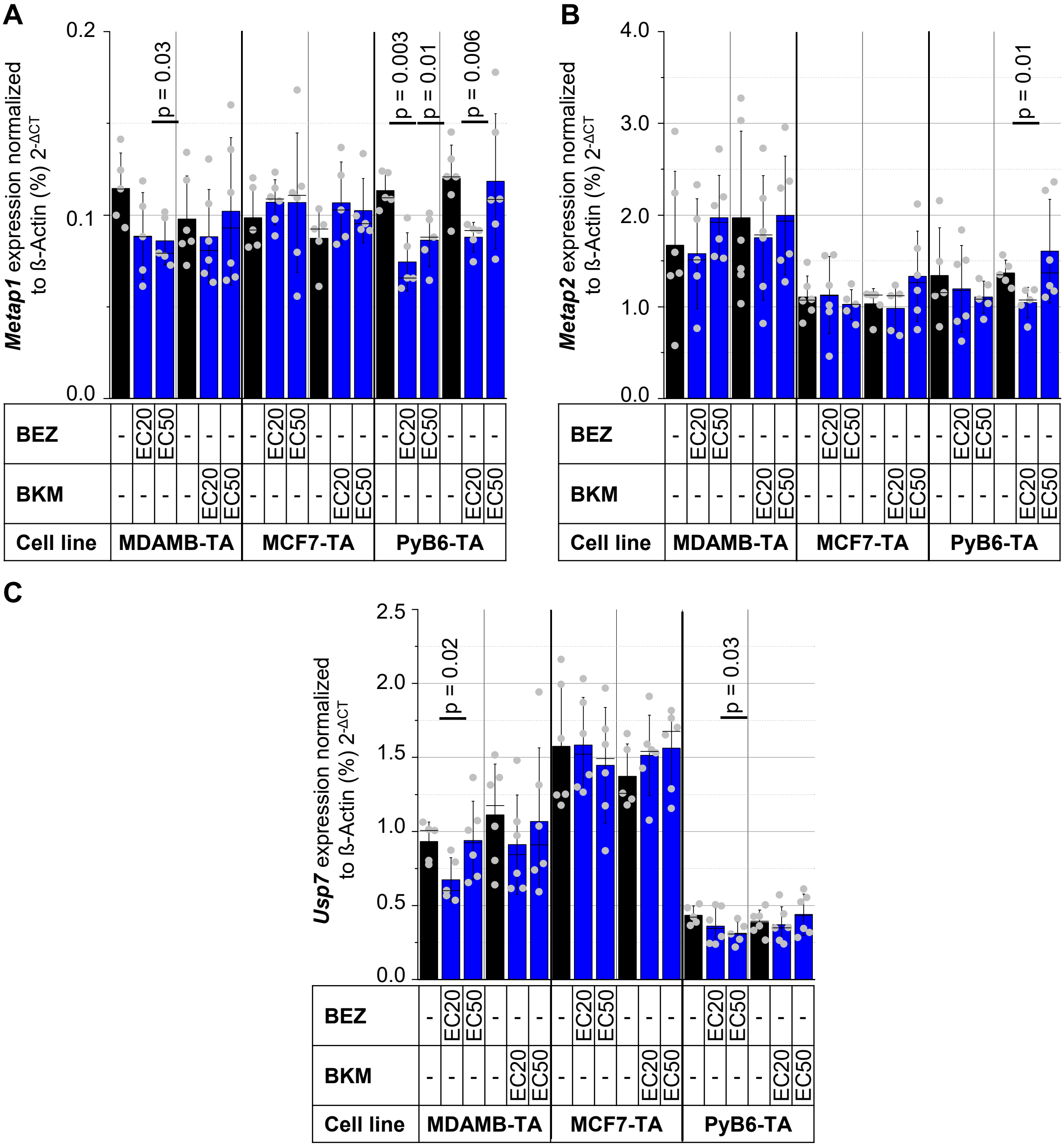

Figure S3: RT-PCR-based analysis of Metap1, Metap2 and Usp7 expression upon PI3K pathway inhibition in human and murine breast cancer cells. Relative mRNA expression levels of human or murine Metap1 (**A**), Metap2 (**B**) or Usp7 (**C**) in miR-E-Renilla-transduced MDAMB-TA, MCF7-TA or PyB6-TA cells cultured for 4 days with PI3K pathway-inhibitors BEZ, BKM or DMSO (control) at EC20 or EC50. MRNA expression as mean + SD percentage of ß-Actin (n = 5-6). Significance (__): two-sample t-test of PI3K-inhibitor treated to DMSO-treated samples; non-equal variances assumed.

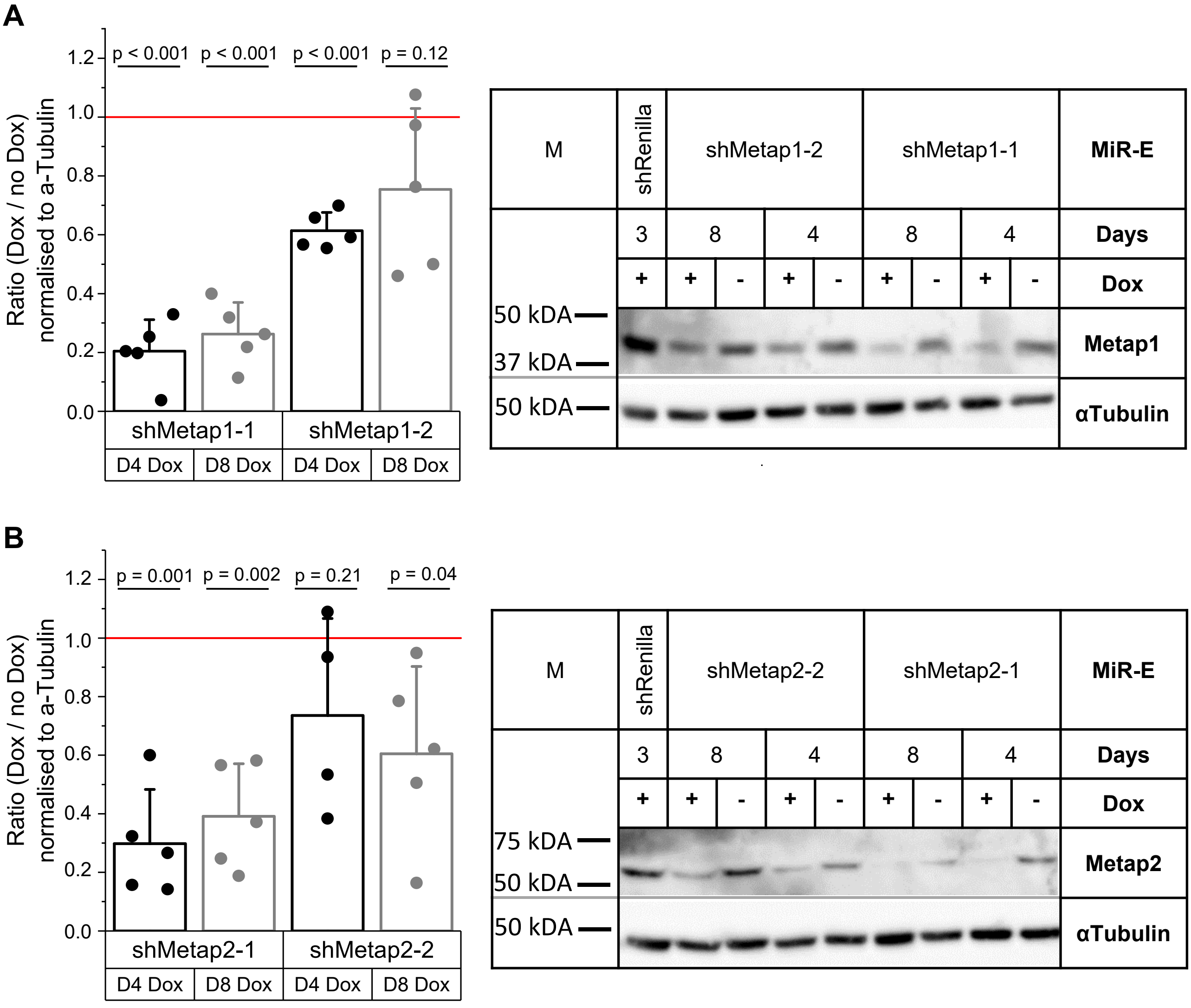

Figure S4: Knockdown of Metap1 and Metap2 in PyB6-TA cells. Analysis of Metap1 (**A**) and Metap2 (**B**) protein expression by Western blot in miR-E-transduced PyB6-TA cells cultured for 4 days (D4) or 8 days (D8) ± Dox treatment. For simplification miR-Es are indicated by sh. Left: Quantification of Western blot data; protein level as mean ratio + SD between Dox-treated miR-E-induced and uninduced cells normalized to α-Tubulin. Significance (p): one-sample t-test to 1 (A: n = 5; B: n = 4-5). Red line: no change in protein level between miR-E induced and uninduced. Right: Representative Western blots; α-Tubulin as loading control; 25 µg protein loaded. M: marker. shRenilla: shRenilla-transduced PyB6-TA control cells.

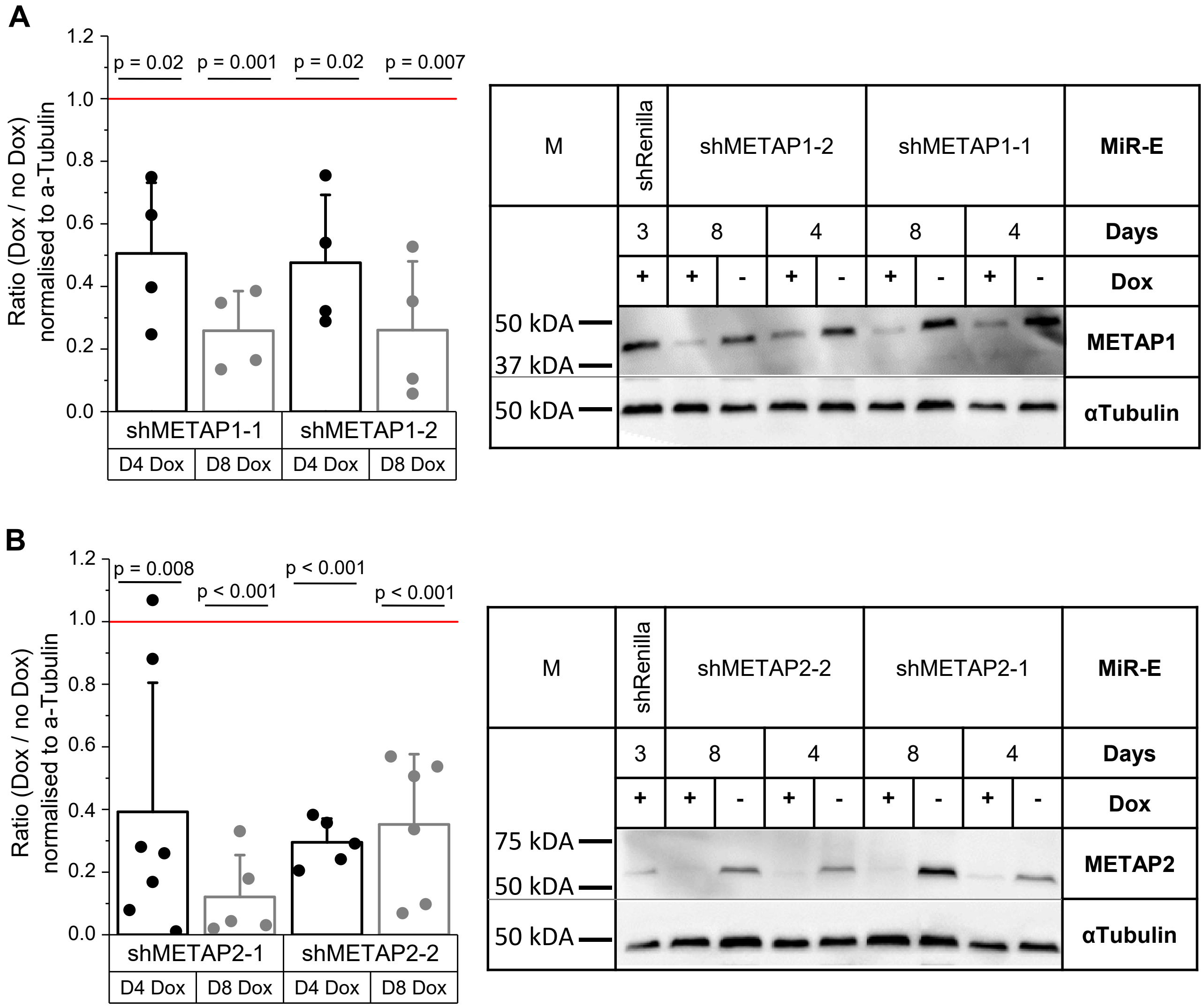

Figure S5: Knockdown of METAP1 and METAP2 in MCF7-TA cells. Analysis of METAP1 (**A**) and METAP2 (**B**) protein expression by Western blot in miR-E-transduced MCF7-TA cells cultured for 4 days (D4) or 8 days (D8) ± Dox treatment. For simplification miR-Es are indicated by sh. Left: Quantification of Western blot data; protein level as mean ratio + SD between Dox-treated miR-E-induced and uninduced cells normalized to α-Tubulin. Significance (p): one-sample t-test to 1 (A: n = 4; B: n = 5-7). Red line: no change in protein level between miR-E induced and uninduced. Right: Representative Western blots; α-Tubulin as loading control; 25 µg protein loaded. M: marker. shRenilla: shRenilla-transduced MCF7-TA control cells. Further experiments were performed with miR-E-METAP1-2 and miR-E-METAP2-2 due to better knockdown potency.

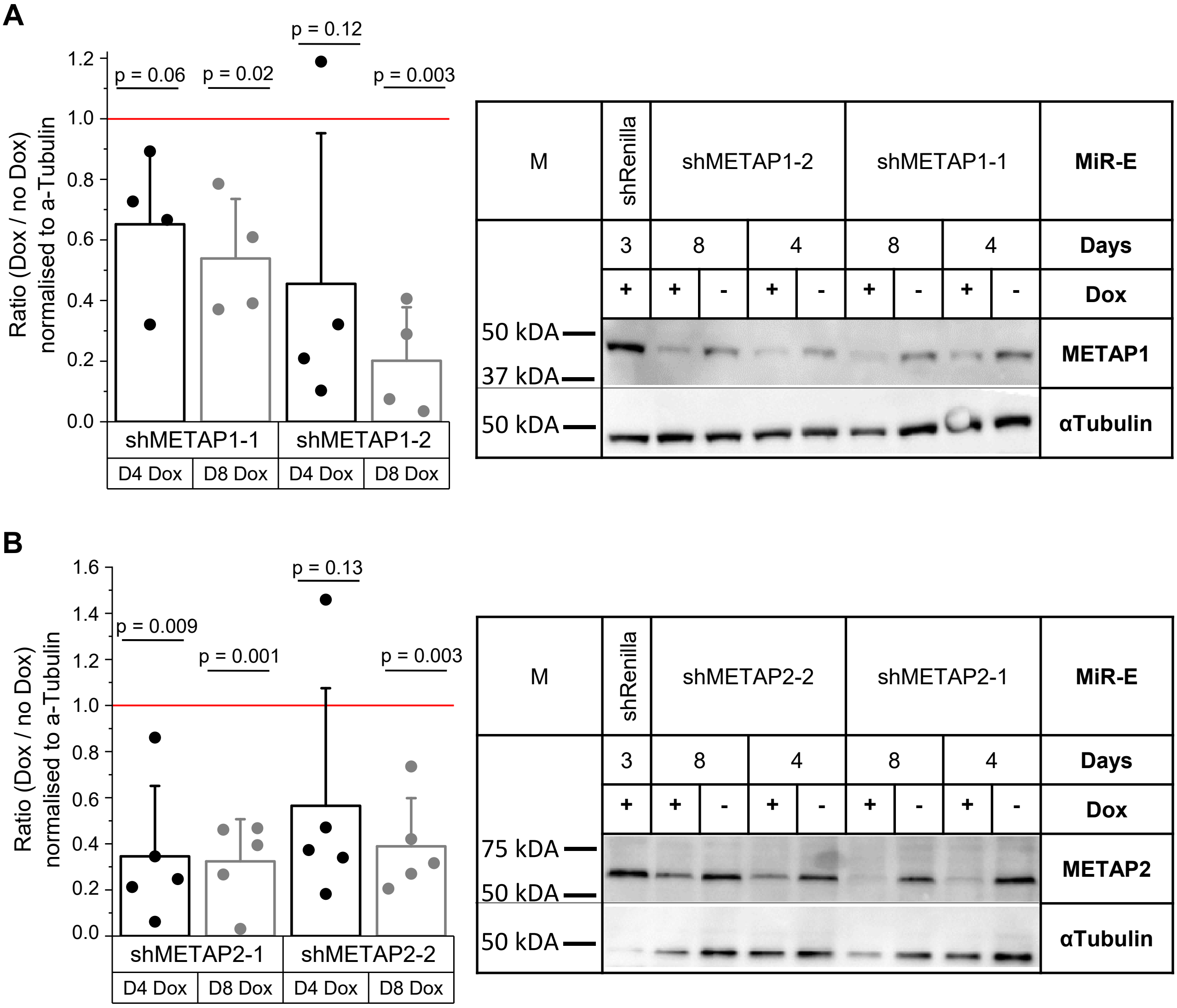

Figure S6: Knockdown of METAP1 and METAP2 in MDAMB-TA cells. Analysis of METAP1 (**A**) and METAP2 (**B**) protein expression by Western blot in miR-E-transduced MDAMB-TA cells cultured for 4 days (D4) or 8 days (D8) ± Dox treatment. For simplification miR-Es are indicated by sh. Left: Quantification of Western blot data; protein level as mean ratio + SD between Dox-treated miR-E-induced and uninduced cells normalized to α-Tubulin. Significance (p): one-sample t-test to 1 (A: n = 4; B: n = 5). Red line: no change in protein level between miR-E induced and uninduced. Right: Representative Western blots; α-Tubulin as loading control; 25 µg protein loaded. M: marker. shRenilla: shRenilla-transduced MDAMB-TA control cells. Further experiments were performed with miR-E-METAP1-2 and miR-E-METAP2-1 due to better knockdown potency.

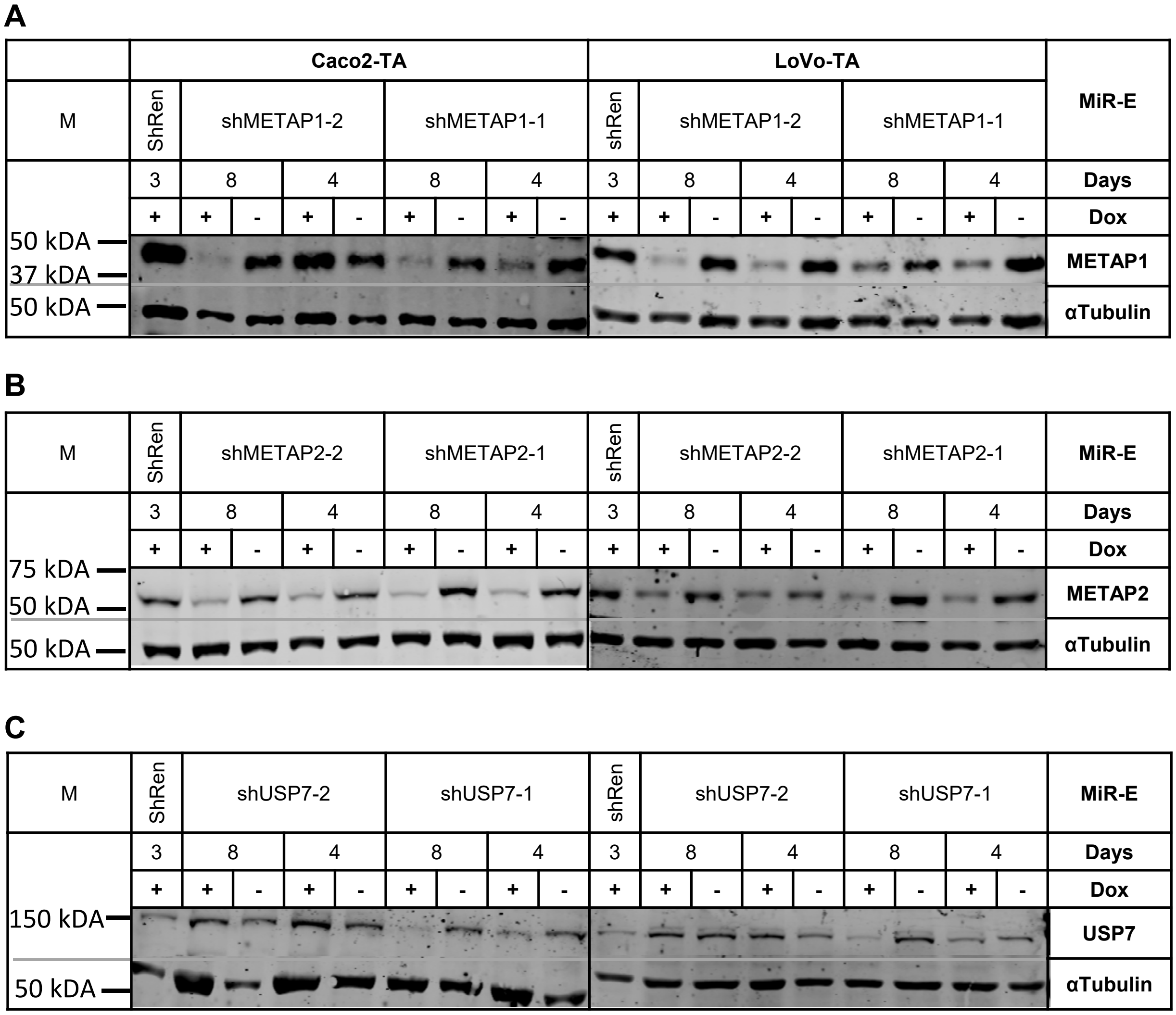

Figure S7: Knockdown of METAP1, METAP2 and USP7 in Caco2-TA and LoVo-TA human colorectal cancer cell lines. Analysis of METAP1 (**A**), METAP2 (**B**) and USP7 (**C**) protein expression by Western blot in miR-E-transduced Caco2-TA and LoVo-TA cells cultured for 4 days or 8 days ± Dox treatment. For simplification miR-Es are indicated by sh. α-Tubulin as loading control; 25 µg or 50 µg (USP7) protein loaded. M: marker. shRen.: shRenilla-transduced control cells. Further experiments in Caco2-TA cells were performed with miR-E-METAP1-1, miR-E-METAP2-1 and miR-E-USP7-1 due to better knockdown potency. In LoVo-TA cells miR-E-METAP1-2, miR-E-METAP2-1 and miR-E-USP7-1 were chosen

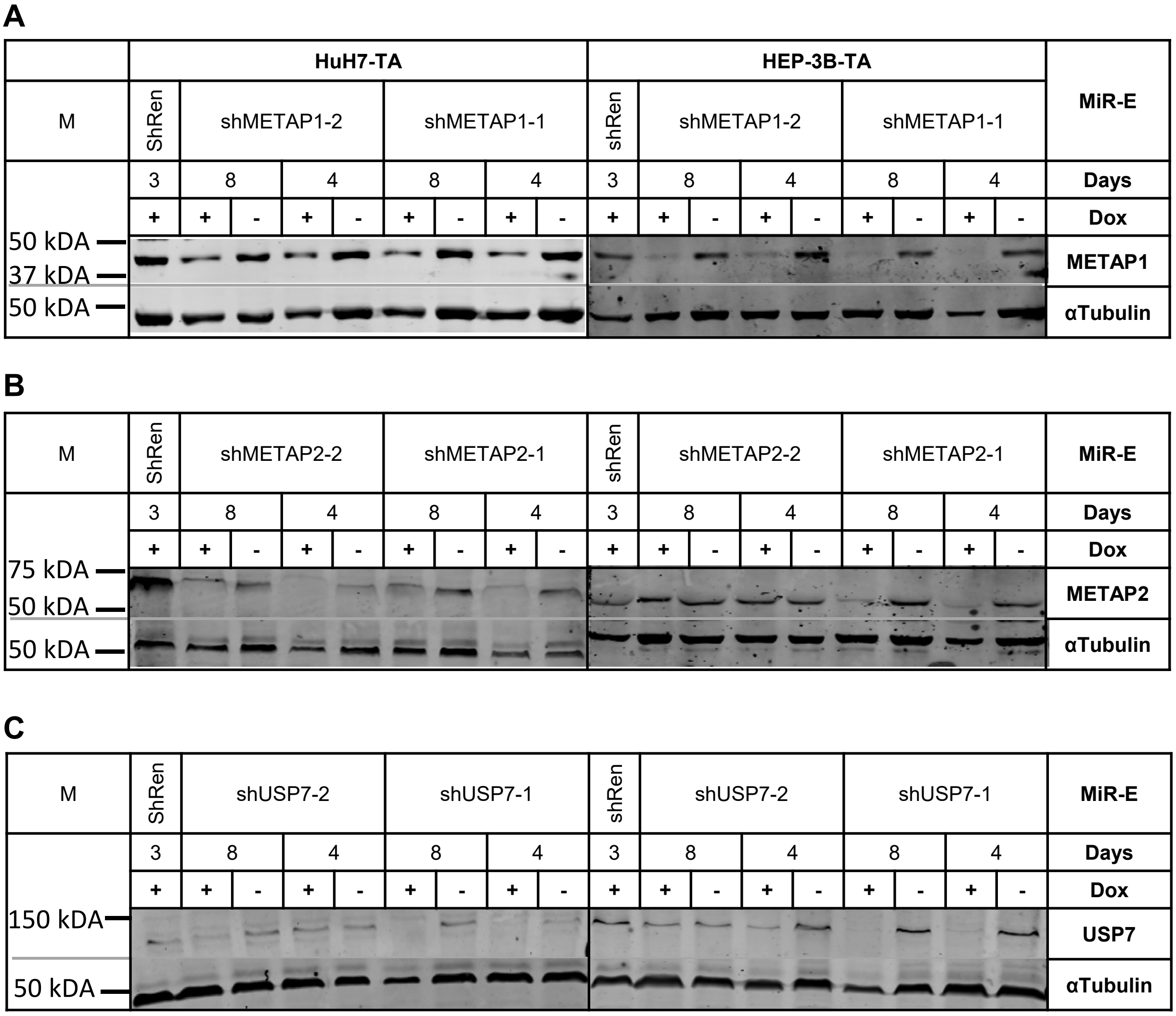

Figure S8: Knockdown of METAP1, METAP2 and USP7 in HuH7-TA and HEP-3B-TA human hepatocellular carcinoma cell lines. Analysis of METAP1 (**A**), METAP2 (**B**) and USP7 (**C**) protein expression by Western blot in miR-E-transduced Huh7-TA and HEP-3B-TA cells cultured for 4 days or 8 days ± Dox treatment. For simplification miR-Es are indicated by sh. α-Tubulin as loading control; 25 µg or 50 µg (USP7) protein loaded. M: marker. shRen.: shRenilla-transduced control cells. Further experiments in HuH7-TA cells were performed with miR-E-METAP1-1, miR-E-METAP2-1 and miR-E-USP7-1 due to better knockdown potency. In HEP-3B-TA cells miR-E-METAP1-1, miR-E-METAP2-1 and miR-E-USP7-1 were chosen.

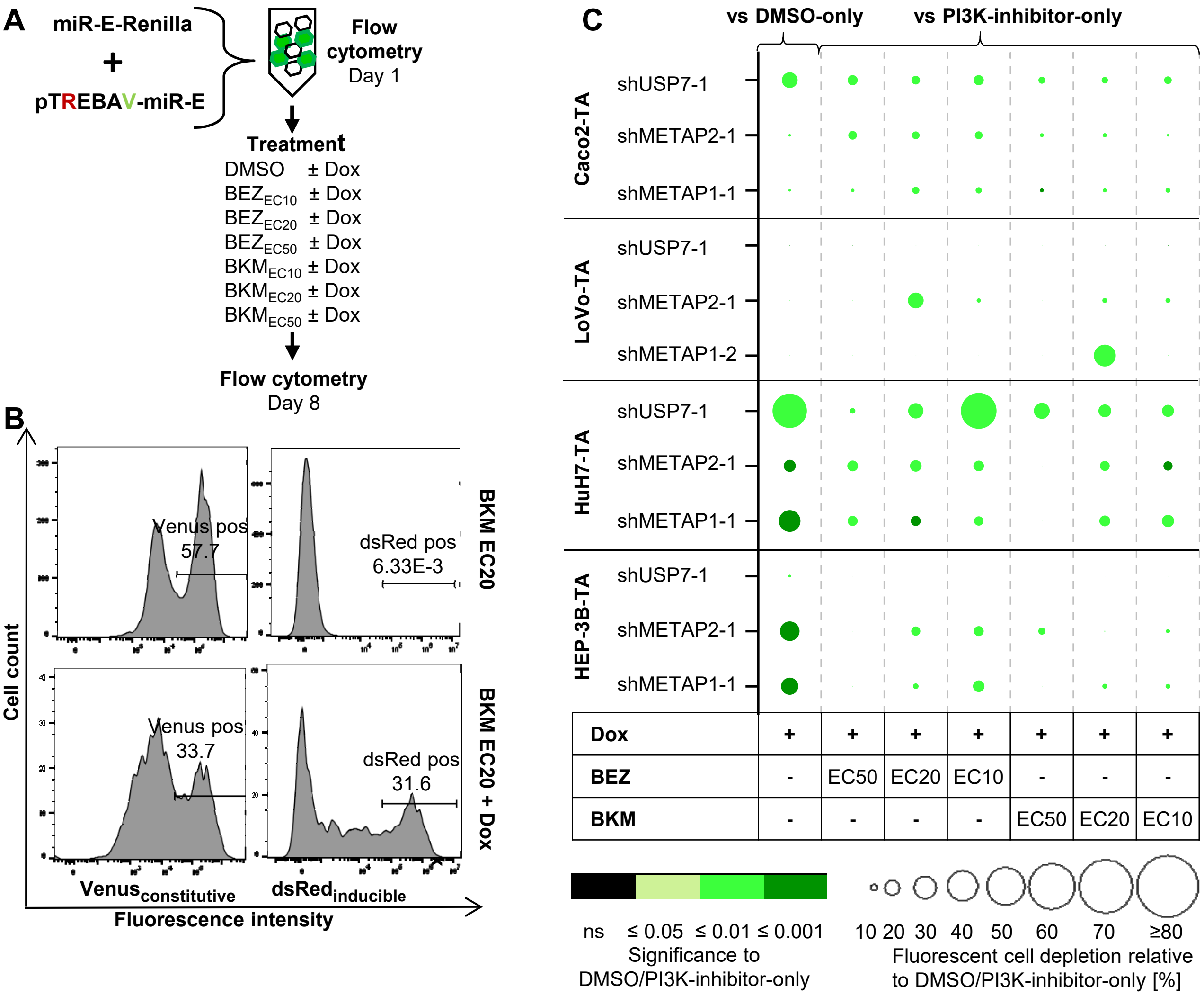

Figure S9: METAP1, METAP2 or USP7 knockdown effects sensitivity of human colorectal cancer and hepatocellular carcinoma cells to PI3K inhibition. **A-C**: Competitive growth flow cytometry assays. **A**: General setup. Flow cytometry-based competitive cell growth analysis upon simultaneous protease targeting and PI3K inhibition in miR-E-transduced Caco2-TA, LoVo-TA, HEP-3B-TA and HuH7-TA cancer cells mixed with miR-E-Renilla transduced controls cells. Cells cultured for 8 days treated as indicated. **B**: Representative flow cytometry histograms for miR-E-METAP1-2-transduced LoVo-TA cells treated with BKM EC20 ± Dox. Fluorescent cells from living, single cells. Gates in %. **C**: Effect of simultaneous protease targeting and PI3K inhibition on competitive cell growth. Circle radius: Mean fluorescent cells depletion relative to DMSO-only or PI3K-inhibitor-only treated cells in %. Circle color: significance of circle radius; one-sample t-test to 0 (n = 4-6). Table with all mean values ± SD in Supplementary Table S3. For simplification miR-Es are indicated by sh. Note that the EC values were determined in 48 h MTT assays.

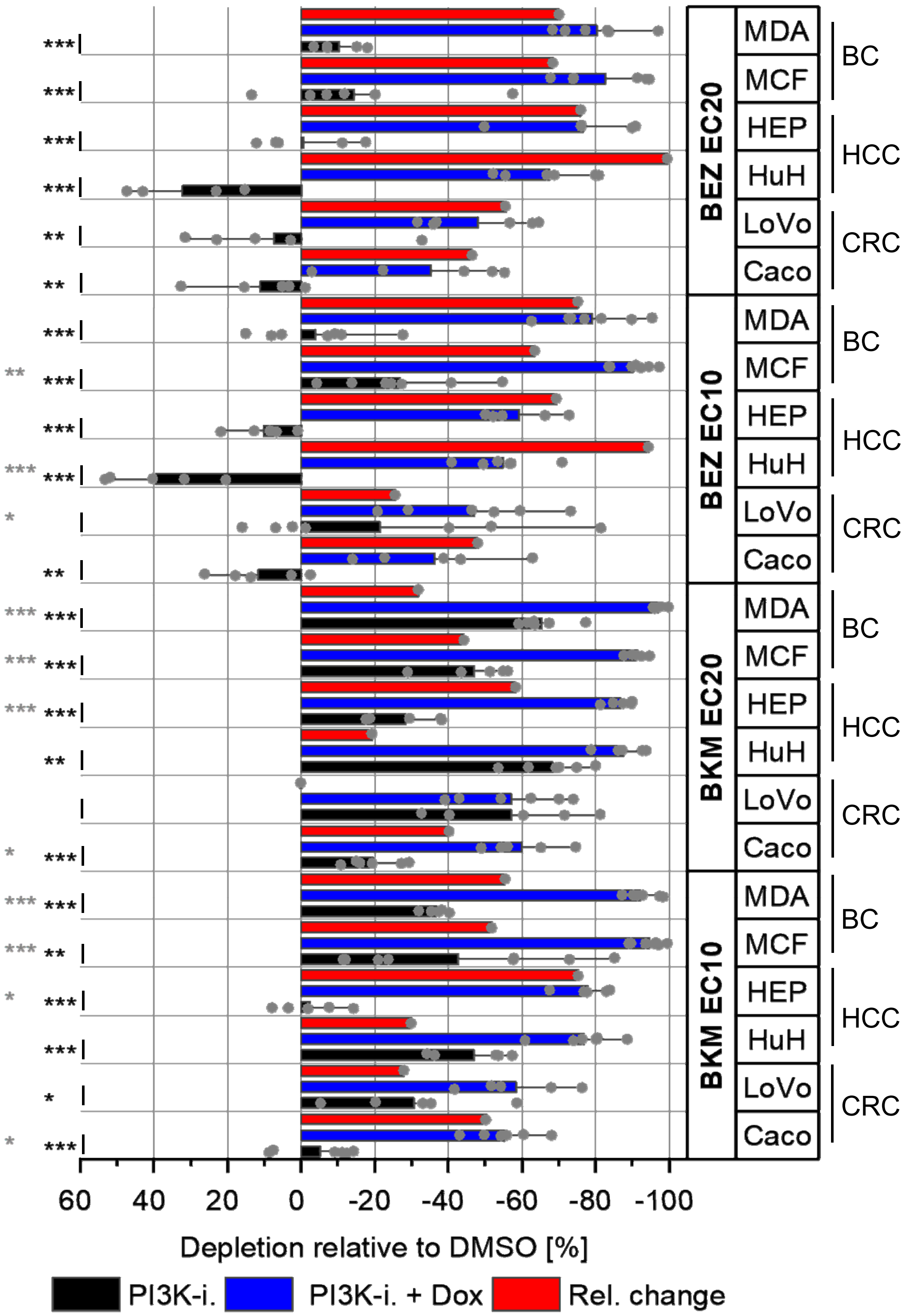

Figure S10: Effects of METAP1 knockdown and combined PI3K inhibition in human colorectal cancer (CRC) and hepatocellular carcinoma (HCC) cells compared to human breast cancer (BC) cells. Crystal violet plate colony formation assays. Mean colony growth reduction upon PI3K-inhibitor-only treatment (PI3K-i) or combined METAP1 knockdown and PI3K-inhibitor treatment (PI3K-i + Dox) relative to DMSO-only in % (n = 5-8). Relative (Rel.) change: Difference between PI3K-inhibitor-only and double treatment. Black *: Significance of growth reduction upon double treatment compared to PI3K-inhibitor-only relative to DMSO. Grey *: Significance in growth reduction between double treatment and knockdown only relative to DMSO; two-sample t-test; non-equal variances assumed. ***: p ≤ 0.001; **: p = 0.01–0.001; *: p = 0.05–0.01. Effects of the knockdown alone as well as a table with all mean values ± SD and p values in Supplementary Table S4. MiR-E transduced cell lines: Caco-2-TA (Caco); LoVo-TA (LoVo); HuH7-TA (HuH); HEP-3B (HEP); MCF7-TA (MCF); MDAMB-TA (MDA). Treatment as indicated for 10-12 days. Data for MCF7-TA and MDAMB-TA is also shown in figure 5. Note that the EC values were determined in 48 h MTT assays.

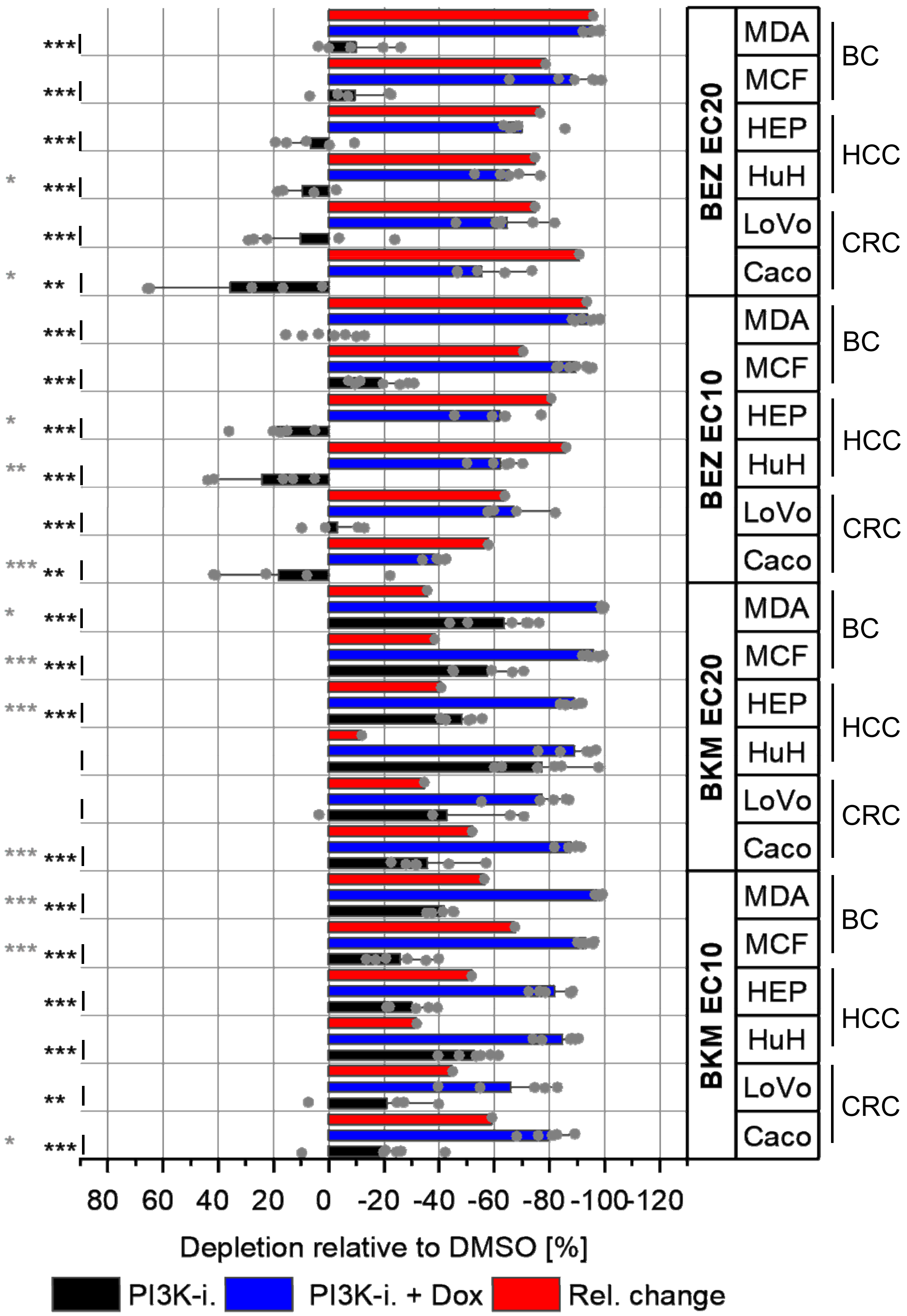

Figure S11: Effects of METAP2 knockdown and combined PI3K inhibition in human colorectal cancer (CRC) and hepatocellular carcinoma (HCC) cells compared to human breast cancer (BC) cells. Crystal violet plate colony formation assays. Mean colony growth reduction upon PI3K-inhibitor-only treatment (PI3K-i) or combined METAP2 knockdown and PI3K-inhibitor treatment (PI3K-i + Dox) relative to DMSO-only in % (n = 4-7). Relative (Rel.) change: Difference between PI3K-inhibitor-only and double treatment. Black *: Significance of growth reduction upon double treatment compared to PI3K-inhibitor-only relative to DMSO. Grey *: Significance in growth reduction between double treatment and knockdown only relative to DMSO; two-sample t-test; non-equal variances assumed. ***: p ≤ 0.001; **: p = 0.01–0.001; *: p = 0.05–0.01. Effects of the knockdown alone as well as a table with all mean values ± SD and p values in Supplementary Table S4. MiR-E transduced cell lines: Caco-2-TA (Caco); LoVo-TA (LoVo); HuH7-TA (HuH); HEP-3B (HEP); MCF7-TA (MCF); MDAMB-TA (MDA). Treatment as indicated for 10-12 days. Data for MCF7-TA and MDAMB-TA is also shown in figure 5. Note that the EC values were determined in 48 h MTT assays.

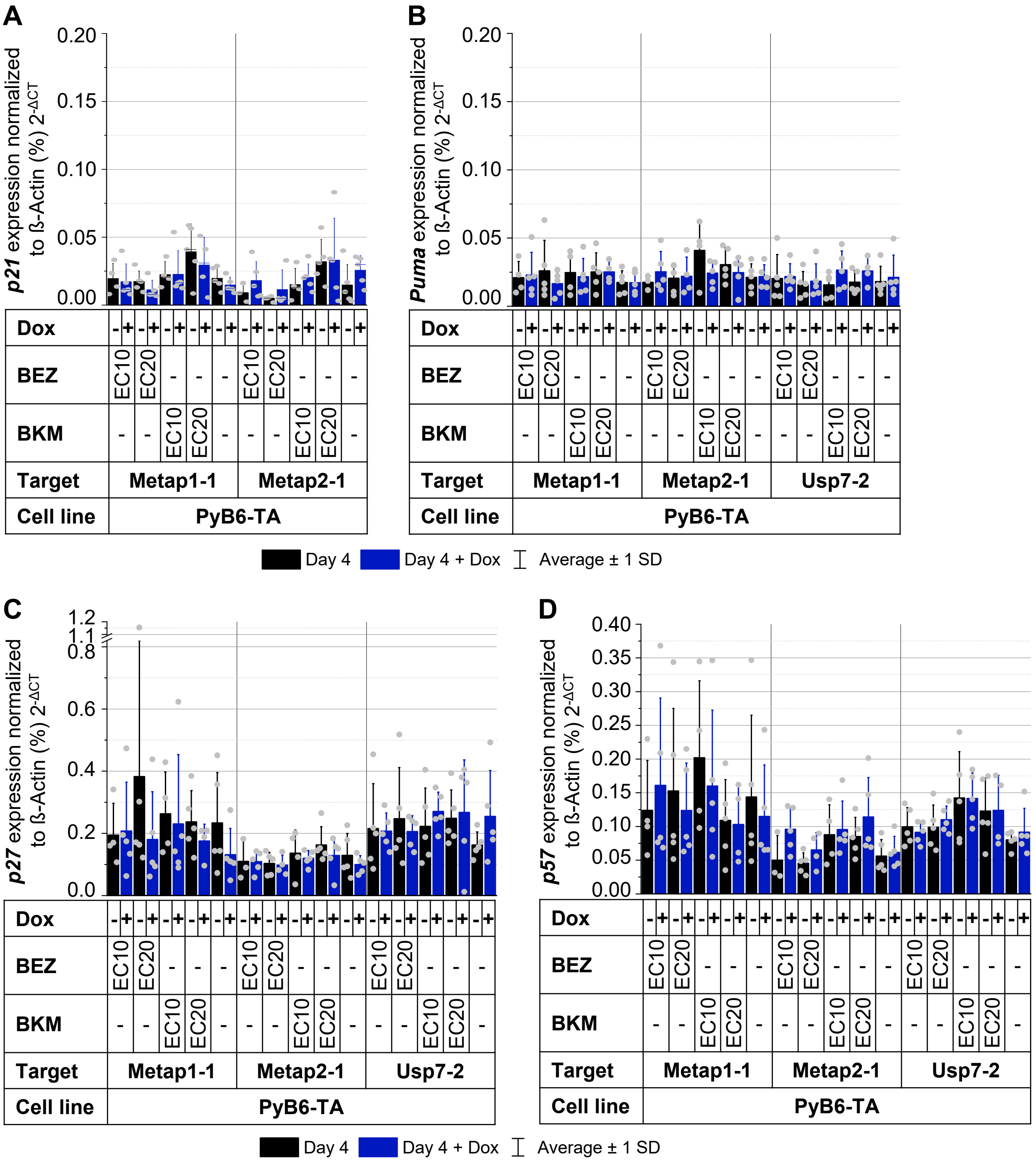

Figure S12: RT-PCR-based analysis of combined PI3K pathway inhibition and candidate protease targeting influence on cell cycle and apoptosis in PyB6-TA cells. Relative mRNA expression levels of p21 (**A**), Puma (**B**), p27 (**C**) and p57 (**D**) in miR-E-transduced PyB6-TA cells cultured for 4 days with PI3K pathway-inhibitors BEZ, BKM or DMSO (control) at EC10 or EC20 ± Dox. For simplification miR-Es are indicated by sh. MRNA expression as mean + SD percentage of ß-Actin (n = 3-5). Significance (__): two-sample t-test of PI3K-inhibitor-Dox treated to PI3K-inhibitor-only treated; non-equal variances assumed. Two-sample t-test of PI3K-inhibitor Dox-treated conditions to DMSO-Dox treated samples were not significant under all conditions. P21 mRNA expression of miR-E-Usp7 transduced cells see Figure 7.

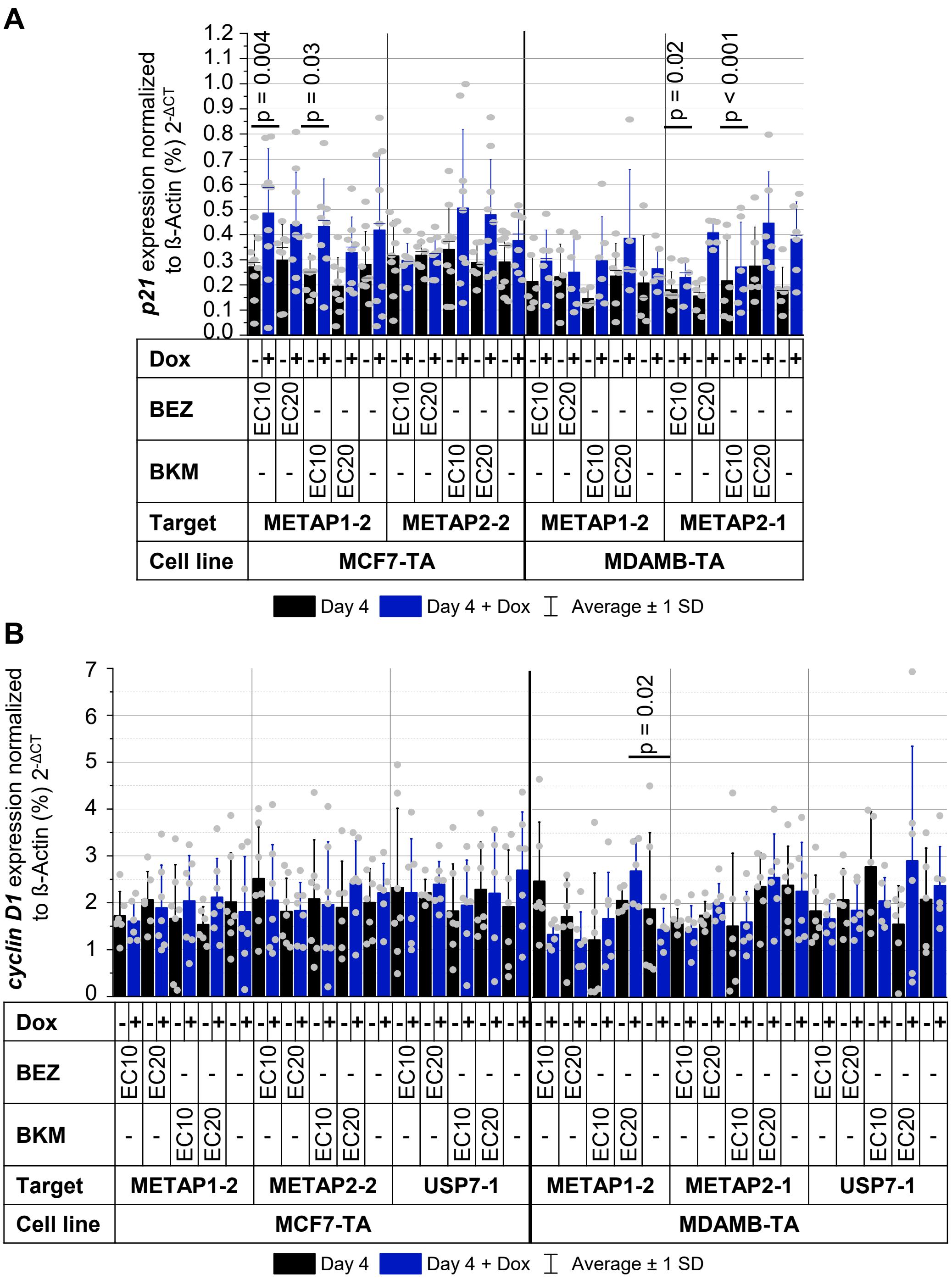

Figure S13: RT-PCR-based analysis of p21 and cyclin D1 expression upon combined PI3K pathway inhibition and candidate protease targeting in human cells. Relative mRNA expression levels of p21 (**A**) and cyclin D1 (**B**) in miR-E-transduced MCF7-TA or MDAMB-TA cells cultured for 4 days with PI3K pathway-inhibitors BEZ, BKM or DMSO (control) at EC10 or EC20 ± Dox. MRNA expression as mean + SD percentage of ß-Actin (n = 5-9 [p21]; n = 5-7 [cyclin D1]). Significance (__): two-sample t-test of PI3K-inhibitor-Dox treated to PI3K-inhibitor-only treated or PI3K-inhibitor-Dox-treated conditions to DMSO-Dox-treated samples; non-equal variances assumed. P21 mRNA expression of miR-E-Usp7 transduced cells see Figure 7.

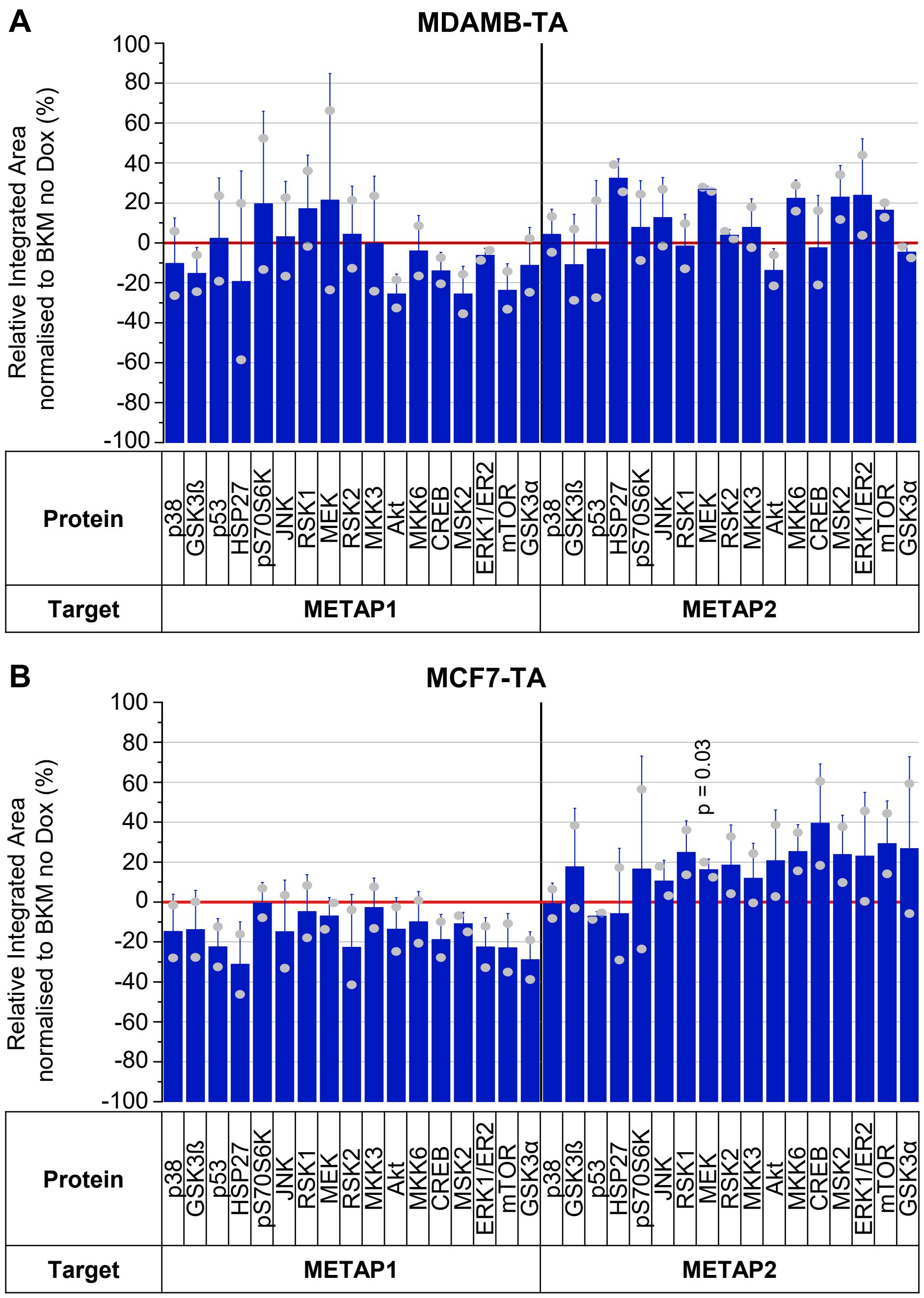

Figure S14: Changes in the relative phosphorylation level of 17 MAPK pathway related proteins upon combined knockdown of METAP1 or METAP2 and BKM treatment in MDAMB-TA (**A**) and MCF7-TA (**B**) cells. Human MAPK phosphorylation antibody array (ab211061; Abcam). Cells treated for 4 days with BKM EC20 ± Dox. Changes in relative phosphorylation level as average integrated area + SD of BKM-Dox treated cells relative to BKM-only treated cells normalized to average reference spots. Significance (__); two sample t-test (n = 2); rest not significant ($p > 0.05$). Phosphorylation sites: p38 (pT180/Y182), GSK3-β (pS9), p53 (pS15), HSP27 (pS82), pS70S6K (pT421/S424), JNK (pT183), RSK1 (pS380), MEK (pS217/221), RSK2 (pS386), MKK3 (pS189), Akt (pS473), MKK6 (pS207), CREB (pS133), MSK2 (pS360), ERK1 (pT202/Y204)/ERK2 (pT185/Y187), mTOR (pS2448), GSK3-α (pS21).

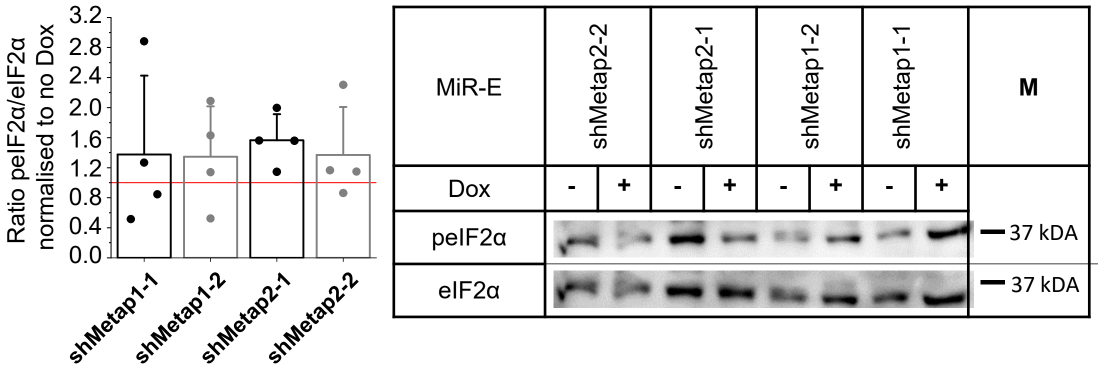

Figure S15: Increased relative phosphorylation of eIF2α upon knockdown of Metap1 or Metap2 in PyB6-TA cells. Relative phosphorylation of peIF2α (Ser51) to non-phosphorylated eIF2α protein expression analysed by Western blot in miR-E-transduced PyB6-TA cells cultured for 4 days ± Dox. Left: Quantification of Western blot data displaying protein level as mean ratio + SD between peIF2α and eIF2α normalized to uninduced cells. Significance (p): one-sample t-test to 1, all not significant ($p > 0.05$). Right: Representative Western blot. M: marker. Red line: no change in relative phosphorylation level between Dox-treated miR-E-induced and uninduced.

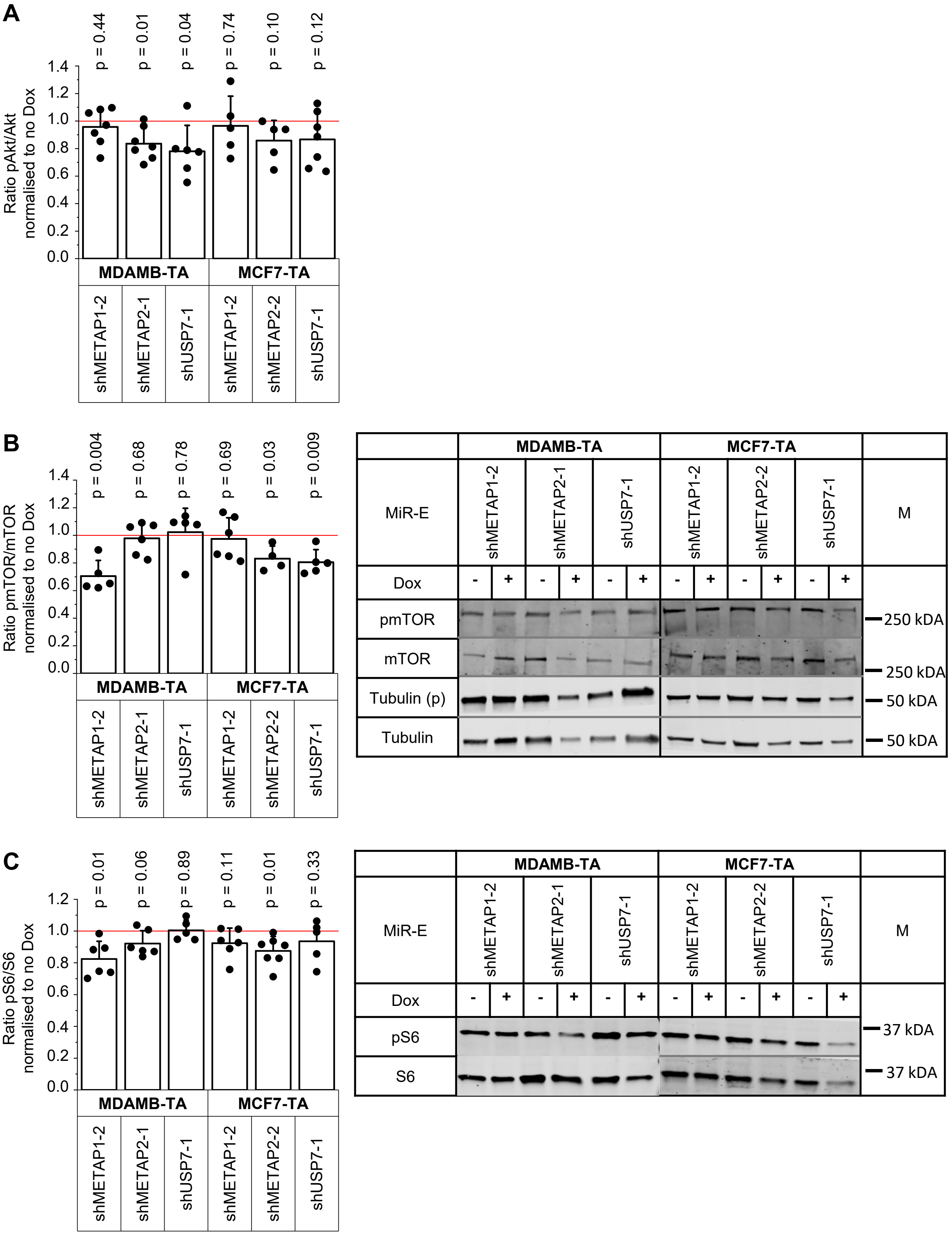

Figure S16: Changes in PI3K pathway activation upon knockdown of METAP1, METAP2 or USP7 in human breast cancer cells. **A**: Relative phosphorylation of AKT (Ser473) to non-phosphorylated Akt protein expression analyzed by ELISA in miR-E-transduced MCF7-TA and MDAMB-TA cells cultured for 4 days ± Dox. **B/C**: Relative phosphorylation of mTOR (**B**; Ser2448) or S6 (**C**; Ser235/236) to non-phosphorylated protein expression analyzed by Western blot in miR-E-transduced MCF7-TA and MDAMB-TA cells cultured for 4 days ± Dox. Left: Quantification of Western blot data, protein level as mean ratio + SD between phosphorylated and total normalized to uninduced cells. MTOR and pmTOR were first normalized on α-Tubulin. Significance (p): one-sample t-test to 1. Red line: no change in relative phosphorylation level between Dox-treated miR-E-induced and uninduced. Right: Representative Western blots. 25 µg protein loaded. pS6 and S6 analyzed on the same membrane. α-Tubulin as loading control for mTOR and pmTOR loaded onto different membranes. M: marker.

A

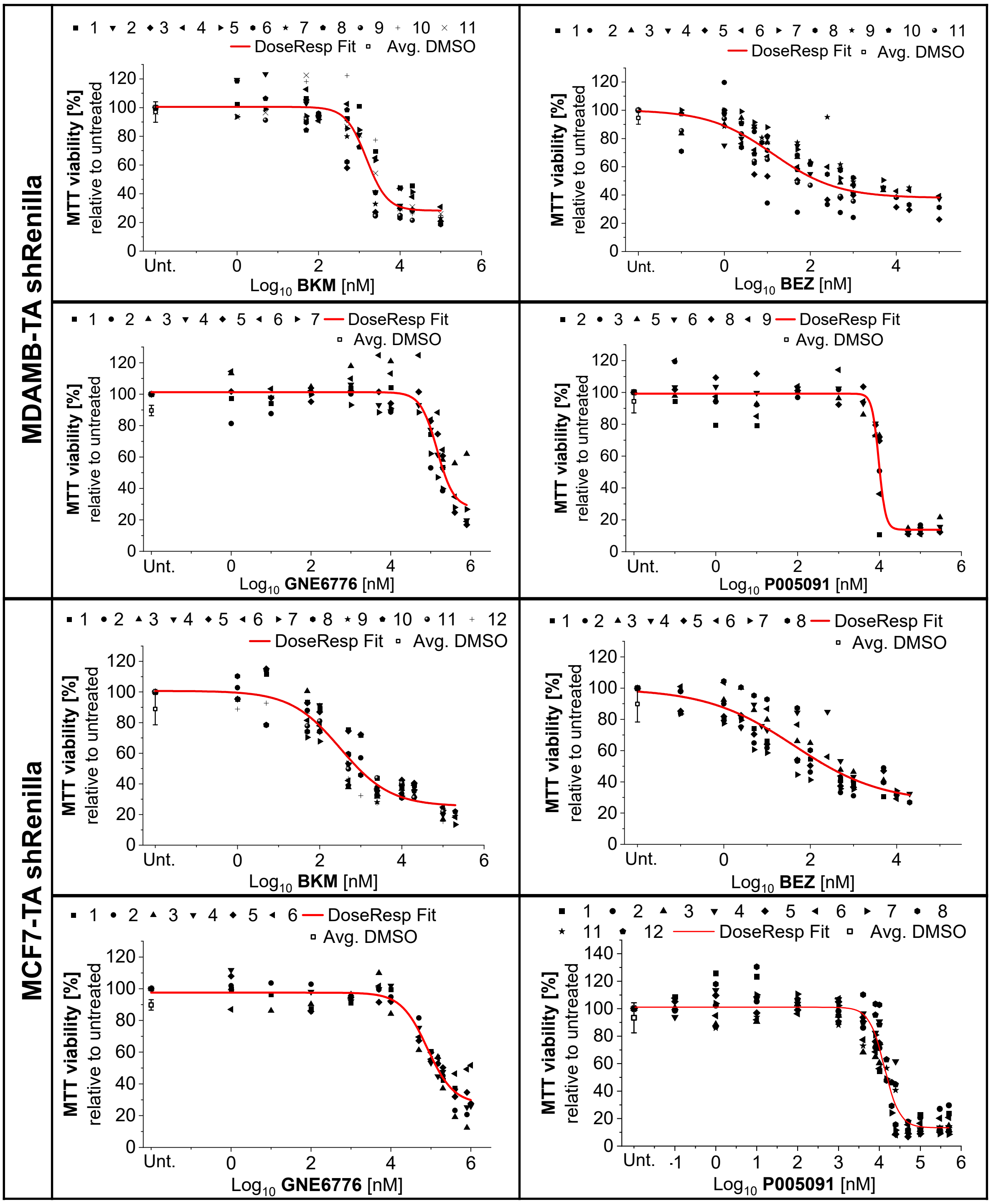

B

	MDAMB-TA shRenilla			MCF7-TA shRenilla		
	**EC20**	**EC50**	**EC80**	**EC20**	**EC50**	**EC80**
**BKM** [µM]	0.67	1.5	3.4	0.05	0.32	2.2
**BEZ** [nM]	1.2	13.5	148.2	1.7	42	1073.2
**GNE6776** [µM]	74.7	145.5	283.5	28.5	81.5	233.4
**P005091** [µM]	7.4	9.6	12.5	7.3	13.3	24.2

Figure S17: PI3K-pathway inhibitor and USP7-inhibitor EC values in human breast cancer cell lines**.** **A**: Dose-response curves in miR-E-Renilla-transduced MDAMB-TA and MCF7-TA cell lines generated by MTT viability assays with 48 h PI3K-pathway inhibitor (BEZ, BKM) or USP7-inhibitor (GNE6776, P005091) treatment (concentration series). Log10 inhibitor concentrations versus MTT viability relative to untreated cells averaged per triplicate. Each symbol represents an independent biological replicate. Dose-response curve calculated with a built-in function of OriginPro (non-lineal fit, category growth/sigmoidal, function: DoseResp) summarized fit for all biological replicates. Avg. DMSO: Average effect in DMSO-treated cells. DoseRespFit: dose-response-curve. Values outside mean ± 2 SD excluded. Unt.: no Inhibitor. **B**: EC20, EC50 and EC80 values determined by the dose-response curves in A. BEZ EC values in nM, other inhibitors in µM.

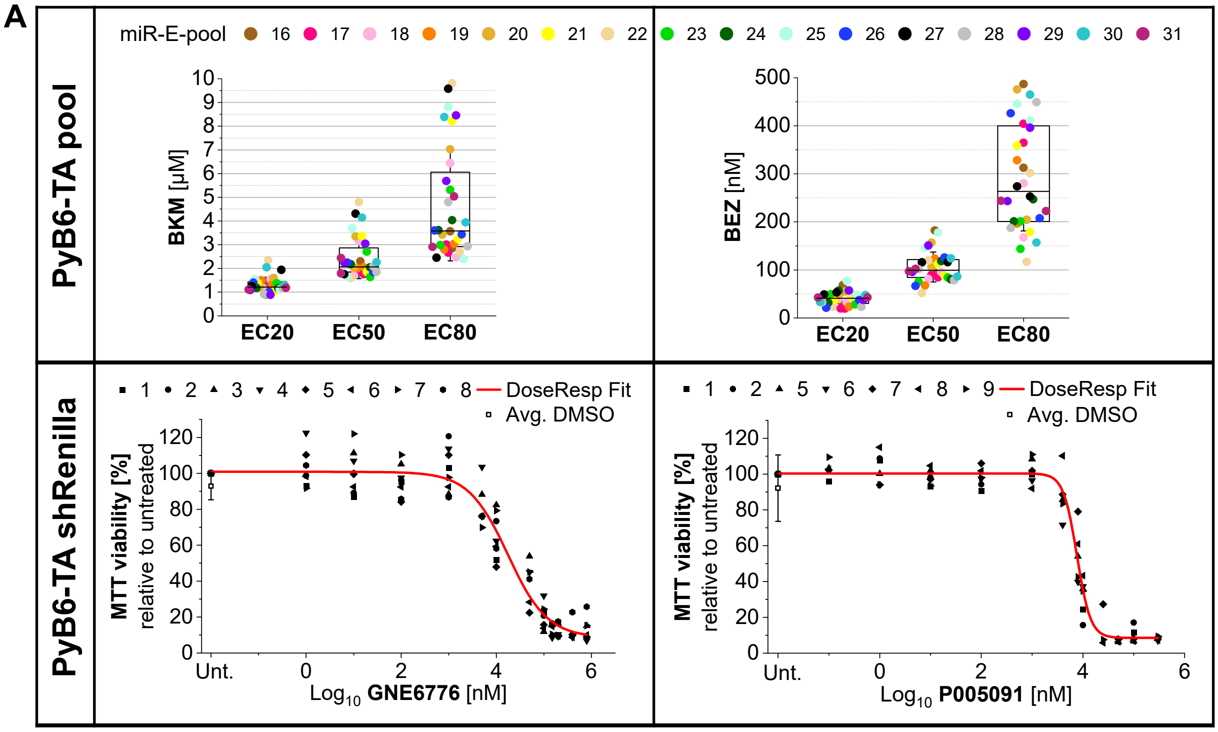

B

	PyB6-TA pool/shRenilla		
	**EC20**	**EC50**	**EC80**
**BKM** [µM]	1.2 (1)	2.1	3.6
**BEZ** [nM]	41	99.4	263.3
**GNE6776** [µM]	4.7	17.6	65.9
**P005091** [µM]	5	7.6	11.7

Figure S18: PI3K-pathway inhibitor and Usp7-inhibitor EC values in murine breast cancer cell lines**. A**: Dose-response curves in PyB6-TA cells generated by MTT viability assays with 48 h PI3K-pathway inhibitor (BEZ, BKM) or Usp7-inhibitor (GNE6776, P005091) treatment (concentration series). For BEZ and BKM the miR-E-pool transduced cell lines (PyB6-TA pool) were used to exclude miR-E pool transduction dependent differences in EC values complicating the degradome-wide screens. The USP7 inhibitors were tested in miR-E-Renilla-transduced cells. Log10 inhibitor concentrations versus MTT viability relative to untreated cells averaged per triplicate. Each symbol represents an independent biological replicate. Dose-response curve calculated with a built-in function of OriginPro (non-lineal fit, category growth/sigmoidal, function: DoseResp) summarized fit for all biological replicates. Avg. DMSO: Average effect in DMSO-treated cells. DoseRespFit: dose-response-curve. Values outside mean ± 2 SD excluded. Unt.: no Inhibitor. **B**: EC20, EC50 and EC80 values determined by the dose-response curves in A. BEZ EC values in nM, other inhibitors in µM. For experiments 1 µM was used as EC20 for BKM.

A

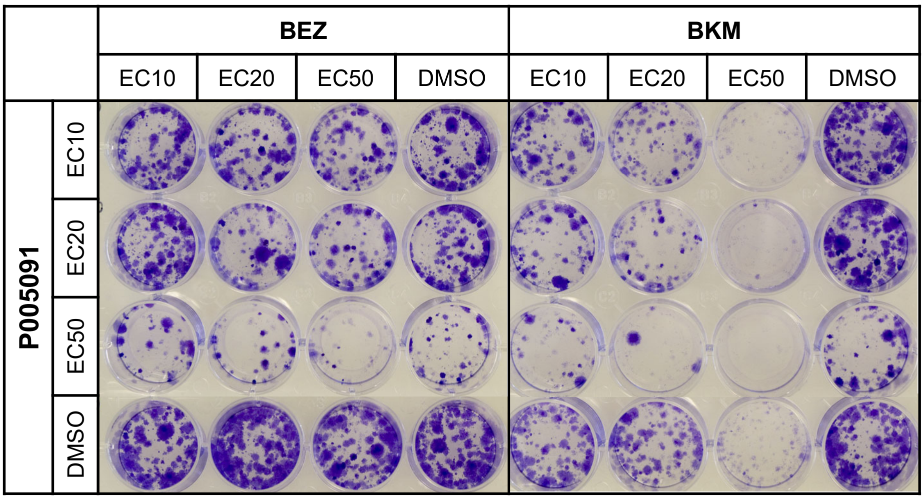

B

P005091	BEZ: DMSO	BEZ: EC10	BEZ: EC20	BEZ: EC50
DMSO		-6.5 ± 14.8	1.1 ± 12.4	-12.1 ± 15.7
EC10	-10.0 ± 17.0	-18.3 ± 8.4	-15.8 ± 15.0	-32.5 ± 15.7
EC20	-22.1 ± 21.1	-18.7 ± 9.1	-30.2 ± 13.4	-50.3 ± 15.3
EC50	-70.4 ± 15.8	-68.0 ± 9.4	-67.3 ± 14.8	-83.6 ± 11.6

P005091	BKM: DMSO	BKM: EC10	BKM: EC20	BKM: EC50
DMSO		-31.5 ± 19.4	-58.7 ± 6.8	-92.8 ± 2.3
EC10	11.7 ± 23.7	-28.8 ± 15.7	-52.4 ± 13.8	-95.0 ± 2.1
EC20	-6.1 ± 15.8	-40.9 ± 17	-72.0 ± 4.0	-96.9 ± 2.0
EC50	-61.2 ± 8.1	-81.7 ± 6.7	-94.2 ± 2.5	-98.3 ± 0.8

C

P005091	BEZ: EC10	BEZ: EC20	BEZ: EC50
EC10	0.17 / 0.37	0.08 / 0.58	0.09 / 0.07
EC20	0.15 / 0.75	0.003 / 0.49	0.003 / 0.04
EC50	<0.001 / 0.77	<0.001 / 0.75	<0.001 / 0.17

P005091	BKM: EC10	BKM: EC20	BKM: EC50
EC10	0.82 / 0.01	0.40 / <0.001	0.20 / <0.001
EC20	0.43 / 0.007	0.01 / <0.001	0.03 / <0.001
EC50	0.001 / 0.004	<0.001 / <0.001	0.006 / <0.001

Depletion relative to DMSO-only [%]

> -20	-20	-30	-40	-50	-60	-70	-80	-90	< -95

Significance to

PI3K-inhibitor-only / P005091-only

p ≤ 0.05

p ≤ 0.01

p ≤ 0.001

Figure S19: Effects of combined Usp7 and PI3K-pathway inhibition in murine breast cancer cells. Crystal violet plate colony formation assays in ShRenilla-transduced PyB6-TA cells treated with BEZ or BKM in combination with P005091 at EC10, EC20 or EC50 for 7-10 days. DMSO was used as control at respective EC50 inhibitor dilutions **A**: Representative pictures. **B**: Mean colony growth reduction relative to DMSO-only treated cells in % ± SD (n = 5-6). **C**: Significance of growth reduction upon double treatment compared to single PI3K-inhibitor treatment or P005091-only treatment to detect synergism; two-sample t-test; non-equal variances assumed. Note that the EC values were determined in 48 h MTT assays.

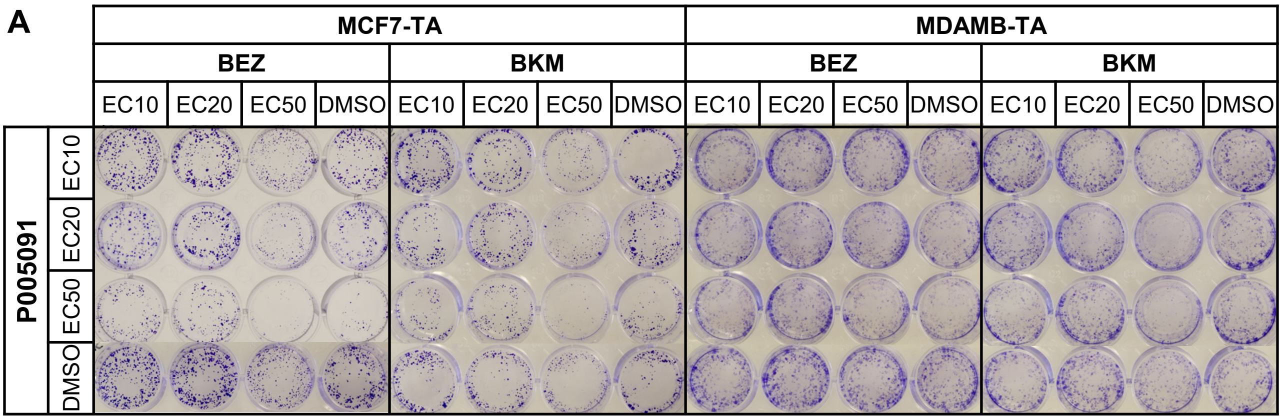

B MCF7-TA

	BEZ			
P005091	DMSO	EC10	EC20	EC50
DMSO		13.4 ± 13.3	17.8 ± 8.8	-51.0 ± 14.8
EC10	-7.2 ± 11.3	10.2 ± 16.4	-12.0 ± 9.3	-57.7 ± 12.7
EC20	-15.4 ± 19.2	13.0 ± 27.0	-3.0 ± 30.3	-69.8 ± 12.2
EC50	-70.2 ± 4.7	-63.3 ± 20.5	-73.0 ± 7.7	-90.4 ± 9.9

	BKM			
P005091	DMSO	EC10	EC20	EC50
DMSO		-2.8 ± 5.1	-7.9 ± 15.5	-55.0 ± 23.5
EC10	-7.6 ± 23.5	0.5 ± 13.6	-10.3 ± 23.7	-62.3 ± 11.0
EC20	1.0 ± 22.8	-21.4 ± 17.1	-16.9 ± 23.4	-72.1 ± 11.5
EC50	-56.0 ± 18.9	-66.9 ± 14.5	-68.7 ± 19.8	-91.0 ± 4.7

C

	BEZ		
P005091	EC10	EC20	EC50
EC10	0.77 / 0.12	0.007 / 0.56	0.51 / <0.001
EC20	0.98 / 0.13	0.25 / 0.51	0.09 / 0.002
EC50	<0.001 / 0.54	<0.001 / 0.61	0.003 / 0.01

	BKM		
P005091	EC10	EC20	EC50
EC10	0.77 / 0.65	0.89 / 0.89	0.65 / 0.02
EC20	0.16 / 0.23	0.60 / 0.38	0.21 / 0.006
EC50	0.002 / 0.46	0.005 / 0.45	0.07 / 0.05

D MDAMB-TA

	BEZ			
P005091	DMSO	EC10	EC20	EC50
DMSO		-30.1 ± 31.7	-7.5 ± 17.3	-11.4 ± 5.6
EC10	-42.4 ± 23.0	-43.7 ± 13.9	-33.1 ± 19.2	-32.4 ± 17.2
EC20	-54.3 ± 21.8	-57.4 ± 6.8	-47.1 ± 16.9	-46.1 ± 20.1
EC50	-68.1 ± 9.7	-63.7 ± 23	-62,4 ± 16.9	-63.6 ± 8.3

	BKM			
P005091	DMSO	EC10	EC20	EC50
DMSO		-19.0 ± 37.1	-9.9 ± 21.0	-16.8 ± 20.8
EC10	-13.3 ± 22.6	-23.5 ± 27.6	-26.8 ± 15.0	-57.6 ± 7.8
EC20	-27.9 ± 16.5	-42.3 ± 26.6	-49.8 ± 10.5	-68.3 ± 9.5
EC50	-54.1 ± 11.5	-57.0 ± 8.6	-57.2 ± 7.3	-79.6 ± 5.3

E

	BEZ		
P005091	EC10	EC20	EC50
EC10	<0.001 / 0.92	0.02 / 0.50	0.87 / 0.45
EC20	<0.001 / 0.78	0.02 / 0.57	0.60 / 0.55
EC50	0.001 / 0.70	<0.001 / 0.75	0.21 / 0.44

	BKM		
P005091	EC10	EC20	EC50
EC10	0.87 / 0.59	0.29 / 0.35	0.01 / 0.01
EC20	0.40 / 0.39	0.04 / 0.06	0.005 / 0.005
EC50	0.17 / 0.70	0.02 / 0.66	0.003 / 0.008

Depletion relative to DMSO-only [%]

> -20	-20	-30	-40	-50	-60	-70	-80	-90	< -95

Significance to

PI3K-inhibitor-only / P005091-only

p ≤ 0.05

p ≤ 0.01

p ≤ 0.001

Figure S20: Effects of combined USP7 and PI3K-pathway inhibition in human breast cancer cells. Crystal violet plate colony formation assays in shRenilla transduced MCF7-TA and MDAMB-TA cells treated with BEZ or BKM in combination with P005091 at EC10, EC20 or EC50 for 10-12 days. DMSO was used as control at respective EC50 inhibitor dilutions **A**: Representative pictures. **B**: Mean colony growth reduction relative to DMSO-only treated cells in % ± SD (MCF7-TA: n = 3-5; MDAMB-TA: 4-6). **C**: Significance of growth reduction upon double treatment compared to single PI3K-inhibitor treatment or P005091-only treatment to detect synergism; two-sample t-test; non-equal variances assumed. Note that the EC values were determined in 48 h MTT assays.

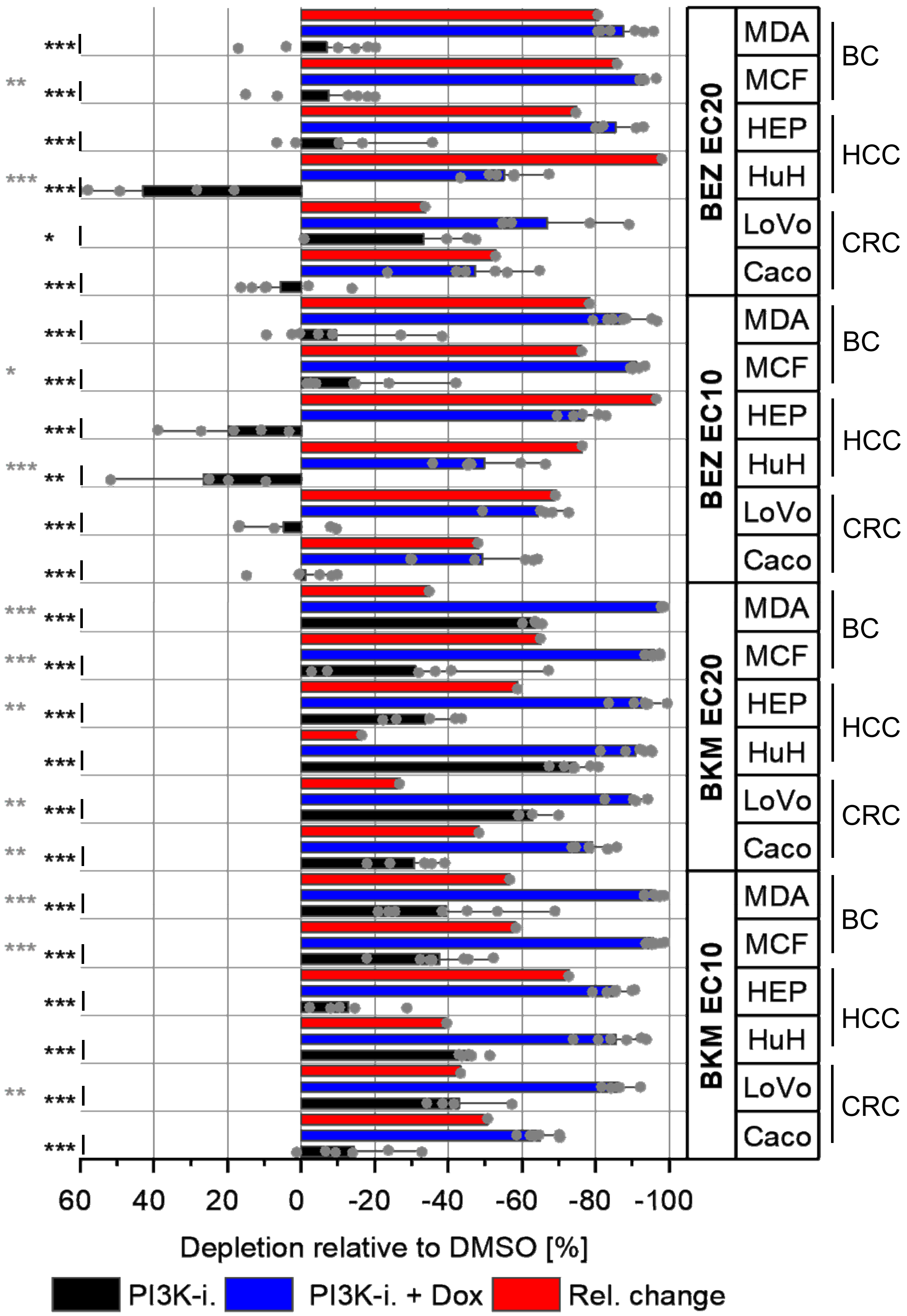

Figure S21: Effects of USP7 knockdown and combined PI3K inhibition in human colorectal cancer (CRC) and hepatocellular carcinoma (HCC) cells compared to human breast cancer (BC) cells. Crystal violet plate colony formation assays. Mean colony growth reduction upon PI3K-inhibitor-only treatment (PI3K-i) or combined USP7 knockdown and PI3K-inhibitor treatment (PI3K-i + Dox) relative to DMSO-only in % (n = 4-7). Relative (Rel.) change: Difference between PI3K-inhibitor-only and double treatment. Black *: Significance of growth reduction upon double treatment compared to PI3K-inhibitor-only relative to DMSO. Grey *: Significance in growth reduction between double treatment and knockdown only relative to DMSO; two-sample t-test; non-equal variances assumed. ***: p ≤ 0.001; **: p = 0.01–0.001; *: p = 0.05–0.01. Effects of the knockdown alone as well as a table with all mean values ± SD and p values in Supplementary Table S4. MiR-E transduced cell lines: Caco-2-TA (Caco); LoVo-TA (LoVo); HuH7-TA (HuH); HEP-3B (HEP); MCF7-TA (MCF); MDAMB-TA (MDA). Treatment as indicated for 10-12 days. Data for MCF7-TA and MDAMB-TA is also shown in figure 5. Note that the EC values were determined in 48 h MTT assays.

.

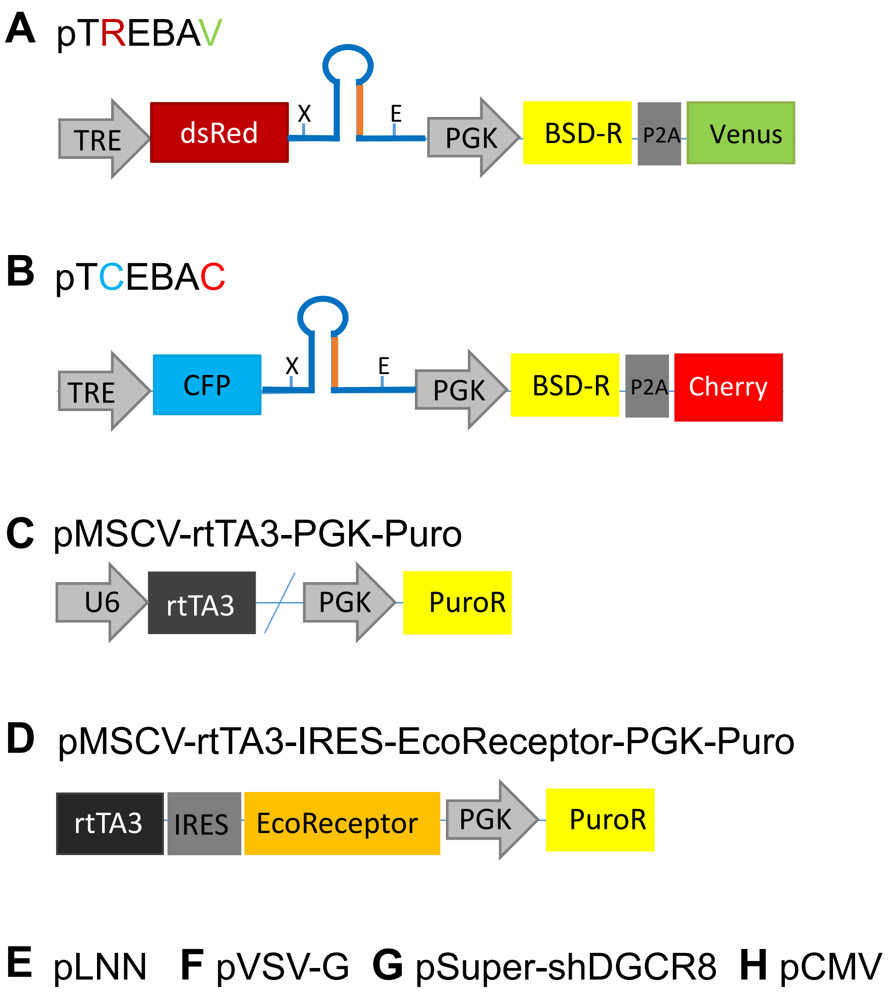

Figure S22: Schematic vector maps with most important features. **A/B**: Dox-inducible double fluorescent retroviral pTREBAV (TRE-dsRed-miR-E-PGK-BSDr-2A-Venus) and pTCEBAC (TRE-Cyan-miR-E-PGK-BSDr-2A-Cherry) vectors were designed based on the pTRMPV vector (Zuber et al.) kindly provided by Dr. Scott W. Lowe (Sloan Kettering Institute, Memorial Sloan Kettering Cancer Center, New York, USA). MiR-E backbone in dark blue, guide sequence in orange. Inducible Tetracycline response element (TRE)-driven Discosoma sp. Red fluorescent protein (dsRed)/Cyan fluorescent protein (Cyan) coupled to the miR-E expression. Constitutive phosphoglycerate kinase promotor (PGK)-driven Blasticidin resistance (BSD-R) and yellow fluorescent protein (Venus)/Cherry fluorescent protein (Cherry) expression separated by co-translational cleavage using the porcine teschovirus-1 2A sequence (P2A). X/E: *XhoI/EcoRI*: restriction enzyme cleavage sites. **C**: Retroviral pMSCV-rtTA3-PGK-Puro plasmid, previously published (Zuber et al.) and kindly provided by Dr. Scott W. Lowe (Sloan Kettering Institute, Memorial Sloan Kettering Cancer Center, New York, USA). Constitutive U6 promoter-driven rtTA3 expression and phosphoglycerate kinase promotor (PGK)-driven Puromycin resistance (PuroR) cassette. **D**: Retroviral pMSCV-rtTA3-IRES-EcoReceptor-PGK-Puror plasmid previously published (Fellmann et al.). RtTA3 coupled to the expression of the Eco Receptor (ecotropic receptor) by an internal ribosomal entry site (IRES). The Eco Receptor allowed for infection of human cells with ecotropic pseudotyped viruses. Constitutive phosphoglycerate kinase promotor (PGK)-driven Puromycin resistance (PuroR) cassette. **E**: Lentiviral plasmid encoding Luciferase (LNN) and a Neomycin resistance cassette for selection. Kindly provided by Prof. Dr. Robert Zeiser (Department of Internal Medicine I, University Clinic, Freiburg, Germany). **F**: Envelope plasmid encoding the VSV-G (vesicular stomatitis virus glycoprotein) envelope protein for pantropic (VSV-G pseudotyped) virus tropism to infect cells of any species. Used to infect human cells. Commercially available (138479; Addgene). **G**: Retroviral plasmid, previously published (Fellmann et al.) encoding a shRNA directed against the microprocessor complex subunit DGCR8 important for miRNA biogenesis to suppresses miR-E maturation in packaging cells preventing the loss of miR-Es from the miR-E library due to targeting effects during retroviral production. **H**: Lentiviral packaging vector encoding Gag for matrix and core proteins and Pol for reverse transcriptase, integrase and RnaseH. Neomycin resistance cassette for selection. Kindly provided by Prof. Dr. Robert Zeiser (Department of Internal Medicine I, University Clinic, Freiburg, Germany).

Resources

Fellmann, C. *et al.* An optimized microRNA backbone for effective single-copy RNAi. Cell Rep. 2013; 5: 1704–13.

Zuber, J. *et al.* Toolkit for evaluating genes required for proliferation and survival using tetracycline-regulated RNAi. Nat Biotechnol. 2011; 29: 79–83.

A

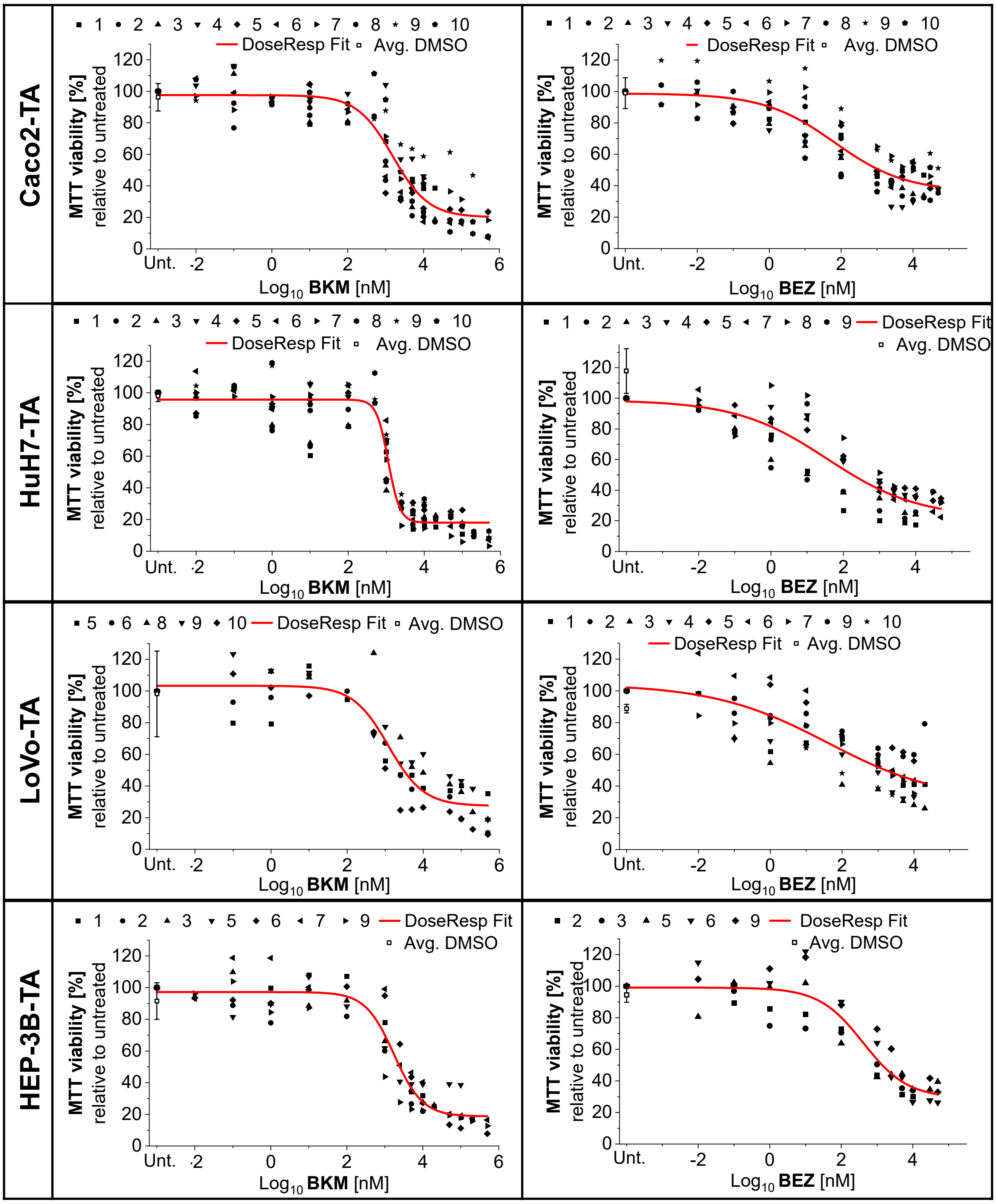

B

	CRC						HCC					
	Caco2-TA			LoVo-TA			HuH7-TA			HEP-3B-TA		
	EC20	EC50	EC80	EC20	EC50	EC80	EC20	EC50	EC80	EC20	EC50	EC80
**BKM** [µM]	0.34	1.5	6.9	0.28	1.2	5.3	0.69	1.1	1.8	0.52	1.7	5.8
**BEZ** [nM]	2.9	65.8	1507.4	0.3	39.4	6335.3	0.7	36.0	2005.3	56.7	409.9	2963.6

Figure S23: PI3K-pathway inhibitor EC values in human colorectal cancer (CRC) and hepatocellular carcinoma (HCC) cell lines. **A**: BEZ and BKM dose-response curves in Caco2-TA and LoVo-TA CRC cell lines as well as in HuH7-TA and HEP-3B-TA HCC cell lines generated by MTT viability assays with 48 h inhibitor treatment (concentration series). Log10 inhibitor concentrations versus MTT viability relative to untreated cells averaged per triplicate. Each symbol represents an independent biological replicate. Dose-response curve calculated with a built-in function of OriginPro (non-lineal fit, category growth/sigmoidal, function: DoseResp) summarized fit for all biological replicates. Avg. DMSO: Average effect in DMSO-treated cells. DoseRespFit: dose-response-curve. Values outside mean ± 2 SD excluded. Unt.: no Inhibitor. **B**: EC20, EC50 and EC80 values determined by the dose-response curves in A. BEZ EC values in nM, BKM in µM.
