# Supplementary material for: RNA interference screens discover proteases as synthetic lethal partners of PI3K inhibition in breast cancer cells
Source: Theranostics. 2022 May 16;12(9):4348–73. doi: 10.7150/thno.68299 (PMC9169373; doi:10.7150/thno.68299)
Supplement: Supplementary file 3 — Supplementary methods. [file thnov12p4348s3.pdf]

Supplementary Methods

Cell line origin and establishment

PyMG-816 stem cell like murine breast cancer cells were generated in-house [Suppl.Ref 1]. PyMG-816-rtTA3-LNN (PyMG-TA) cells were generated by double infection of PyMG-816 cells with undiluted ectopically packed pMSCV-rtTA3-PGK-Puro and pantopically packed p-LNN virus supplemented with Polybrene [4 µg/ml] 24 h after seeding. Infection was repeated after 24 h followed by Puromycin (4 µg/ml) and Neomycin (500 µg/ml) selection until freezing. PyB6-313 murine breast cancer cells were generated in-house as previously described [termed Ctsd +/+ in Suppl.Ref 2]. PyB6-313-rtTA3-LNN (PyB6-TA) cells were generated as described above for PyMG-TA cells. Normal murine mammary gland epithelial cells subclone E9 (NMUMG/E9 [Suppl.Ref 3]) were kindly provided by Prof. Dr. Gerhard Christofori (Department of Biomedicine, University of Basel, Switzerland). NMuMG/E9-rtTA3 (NMuMG/E9-TA) cells were generated by transduction of NMuMG/E9 with undiluted ectopically packed pMSCV-rtTA3-PGK-Puro virus supplemented with Polybrene [8 µg/ml] 6 h after seeding followed by Puromycin (2.25 µg/ml) selection until freezing.

Human breast cancer cell lines, MCF7 (luminal-A, ATCC®; HTB-22™) and MDA-MB-231 (triple-negative, ATCC®; HTB-26™), were purchased from ATCC. MCF7-rtTA3-EcoReceptor (MCF7-TA) and MDA-MB-231-rtTA3-EcoReceptor (MDAMB-TA) cells were generated by transduction of MCF7 or MDA-MB-231 cells with 1:1 diluted pantopically packed pMSCV-rtTA3-IRES-EcoReceptor-PGK-Puro virus supplemented with Polybrene [8 µg/ml] 24 h after seeding. Spin-infection (15 min, 449 rcf) was performed 3 times every 12 h, followed by Puromycin selection (MDAMB-TA: 0.6 µg/ml; MCF7-TA: 1.1 µg/ml) until cells were frozen.

Human colorectal adenocarcinoma cell lines Caco2 (ATCC®; HTB-37) and LoVo (CLS®; 300266) as well as hepatocellular carcinoma cell lines HuH7 (CLS®; 300156) and HEP-3B (DSMZ®; ACC93) are commercially available. Introduction of the pMSCV-rtTA3-IRES-EcoReceptor-PGK-Puro vector was done as described above for MDAMB-TA and MCF7-TA cells, generating the Caco2-rtTA3-EcoReceptor (Caco2-TA), LoVo-rtTA3-EcoReceptor (LoVo-TA), HuH7-rtTA3-EcoReceptor (HuH7-TA) and HEP-3B-

rtTA3-EcoReceptor (HEP-3B-TA) cell lines. Puromycin selection was performed with 2 µg/ml increased to 4 µg/ml after 5 days.

Platinum-E (Plat-E [Cell Biolabs Inc.; RV-101]), HEK-293T (ATCC®; CRL-3216™) and Phoenix-GP (ATCC®; CRL-3215™) packaging cells were purchased.

All cell lines were tested for Mycoplasma contaminations prior freezing and transduction in-house.

Sequence processing and effect calculation

Sequence processing was performed using customized Python 2.7 scripts. Conversion of Fastq files into FASTA files, sequence de-multiplexing according to their barcode (one mismatch per barcode allowed), reverse-complementing, trimming to sense-loop-guide region, collapsing (FASTX Toolkit) and alignment of collapsed sequences against the library and the 4 internal controls (BLAST-like alignment tool algorithm [Suppl.Ref 4]; one missmatch in seed region and Ns in loop allowed) generated the raw number of reads of each miR-E in the library per sample. Data processing of the raw reeds was done using experiment-specific R-scripts. R-scripts can be provided upon reasonable request to the first author. In brief, reads that didn't belong to the used miR-E-pool or were higher than 20000 were excluded (cleaning). Furthermore, samples with more than 100 NA values (not analyzed; miR E-not detected) in one miR-E-pool and samples for which the sum of all miR-E´s in the pool was below 15000 were excluded. Pool-based normalization was performed to correct for variability within the samples of one barcode (number of reads for each miR-E / total number of reads per miR-E-pool x 10^6) and data was Log2 transformed. Further processing included deletion (trimming) of reads to minimize random variation whereby DMSOBEZ, DMSOBEZ + Dox, BEZ and BEZ + Dox reads were deleted if the corresponding DMSOBEZ reads were < 20. BKM samples were trimmed due to the same schema. If DMSOBEZ/DMSOBKM samples used for trimming were deleted due to bad quality of the whole sample (NA > 100 or pool sum < 15000 [see above]) the corresponding samples were not trimmed. Un-trimmed samples and removed miR-E-pools are indicated in the respective R-scripts with reason for exclusion. For effect calculation the robust strictly standardized median difference

(AvSSMD*) calculated after a modified version of the method-of-moment estimate of the paired SSMD* by Zhang X.D [Suppl.Ref 5] was chosen being suitable for screens with biological duplicates only.

I = Average (NOR_DMSO + NOR_Inhibitor) – 20%

FGR = NOR_InhibitorDox/I

BGR = NOR_DMSO/NOR_Inhibitor

Foreground (FGR): difference of a specific miR-E between treated and untreated conditions; background (BGR): difference between two untreated conditions. NOR_: Number of reads of a specific miR-E in the respective sample. I = inhibitor effect calculated from normalized, not log2 transformed reads independently for BKM and BEZ. I was used instead of the respective NOR_Inhibitor sample because PI3K-inhibitor treatment affected all uninduced miR-E-transduced cells equally. Hence, the effect of the PI3K-inhibitor treatment alone was not measurable when comparing the relative miR-E distribution between PI3K-inhibitor-only and DMSO-only samples. InhibitorDox = BEZ + DOX or BKM + DOX sample; DMSO = corresponding DMSOBEZ or DMSOBKM samples; Inhibitor = BEZ or BKM treated samples. If the DMSO sample of one PI3K-inhibitor was excluded due to bad quality the corresponding DMSO sample with the DMSO concentration of the other PI3K-inhibitor was used to calculate I.

SSMDR1* = (FGR_1-medianBGR)/MAD * √2

SSMDR2* = (FGR_2-medianBGR)/MAD * √2

AvSSMD* = Average (SSMDR1* + SSMDR2*)

AvSSMD*/Protease = Average (AvSSMD*of all miR-Es targeting the same transcript)

The SSMD was calculated for each miR-E in both biological replicates independently (_1/ _2 [Replicate 1 and 2]). MedianBGR: Median of BGR from all miR-Es in both replicates. Median Absolute Deviation (MAD): 1.4826 * Median from the absolute values of BGRReplicate1-MedianBGR and BGRReplicate2-MedianBGR. AvSSMD* = Average of the SSMD* from both replicates thereby allowing to keep the value of one replicate if the other is empty (use of only one is highlighting for score calculation.*

Supplementary Methods References

Suppl. Ref 1. Hillebrand LE, Wickberg SM, Gomez-Auli A, et al. MMP14 empowers tumor-initiating breast cancer cells under hypoxic nutrient-depleted conditions. FASEB J. 2019; 33: 4124–40.

Suppl. Ref 2. Ketterer S, Mitschke J, Ketscher A, et al. Cathepsin D deficiency in mammary epithelium transiently stalls breast cancer by interference with mTORC1 signaling. Nat Commun. 2020; 11: 5133.

Suppl. Ref 3. Maeda M. Cadherin switching: essential for behavioral but not morphological changes during an epithelium-to-mesenchyme transition. J Cell Sci. 2005; 118: 873–87.

Suppl. Ref 4. Kent WJ. BLAT---The BLAST-Like Alignment Tool. Genome Res. 2002; 12: 656–64.

Suppl. Ref 5. Zhang XD. Illustration of SSMD, z score, SSMD*, z* score, and t statistic for hit selection in RNAi high-throughput screens. J Biomol Screen. 2011; 16: 775–85.
